# Dental integration and modularity in pinnipeds

**DOI:** 10.1038/s41598-019-40956-1

**Published:** 2019-03-12

**Authors:** Mieczyslaw Wolsan, Satoshi Suzuki, Masakazu Asahara, Masaharu Motokawa

**Affiliations:** 1 Museum and Institute of Zoology, Polish Academy of Sciences, Wilcza 64, 00-679 Warszawa, Poland; 2 Kanagawa Prefectural Museum of Natural History, 499 Iryuda, Odawara, Kanagawa 250-0031 Japan; 3 Division of Liberal Arts and Sciences, Aichi Gakuin University, Iwasaki-cho-Araike 12, Nisshin, Aichi 470-0195 Japan; 4 The Kyoto University Museum, Kyoto University, Kyoto, 606-8501 Japan

## Abstract

Morphological integration and modularity are important for understanding phenotypic evolution because they constrain variation subjected to selection and enable independent evolution of functional and developmental units. We report dental integration and modularity in representative otariid (*Eumetopias jubatus*, *Callorhinus ursinus*) and phocid (*Phoca largha*, *Histriophoca fasciata*) species of Pinnipedia. This is the first study of integration and modularity in a secondarily simplified dentition with simple occlusion. Integration was stronger in both otariid species than in either phocid species and related positively to dental occlusion and negatively to both modularity and tooth-size variability across all the species. The canines and third upper incisor were most strongly integrated, comprising a module that likely serves as occlusal guides for the postcanines. There was no or weak modularity among tooth classes. The reported integration is stronger than or similar to that in mammals with complex dentition and refined occlusion. We hypothesise that this strong integration is driven by dental occlusion, and that it is enabled by reduction of modularity that constrains overall integration in complex dentitions. We propose that modularity was reduced in pinnipeds during the transition to aquatic life in association with the origin of pierce-feeding and loss of mastication caused by underwater feeding.

## Introduction

Organisms are organised into multiple identifiable parts on multiple levels. These parts are distinct from each other because of structure, function or developmental origins. The fact that parts of an organism are distinguishable reflects their individuality and a degree of independence from each other. Nevertheless, these different parts must be coordinated in their size and shape and integrated throughout the entire organism to make up a functional whole. Tension between the relative independence and the coordination of organismal parts is expressed in concepts of morphological integration^1^ and modularity^2,3^. Both concepts are closely related and concern the degree of covariation or correlation between different parts of an organism or other biological entity. Integration deals with the overall pattern of intercorrelation, and modularity involves the partitioning of integration into quasi-independent partitions. Integration exists if parts vary jointly, in a coordinated fashion, throughout a biological entity. Modularity exists if integration is concentrated within certain parts that are tightly integrated internally but is weaker between those parts. Parts that are integrated within themselves and relatively independent of other such internally integrated parts are called modules^4–6^. Integration and modularity are seen at various levels of biological organisation, from genes to colonies, not only in a morphological context but also in other contexts (e.g. molecular^7^, metabolic^8^, ecological^9^), and are viewed as a general property of many different webs of interactions beyond biology^4^.

Morphological integration and modularity have received increased attention among modern evolutionary biologists because the integrated and modular organisation of biological entities has important implications for understanding phenotypic evolution. Integration constrains the variability of individual traits, and modularity enables modules to vary and evolve independently of each other whilst still maintaining the integrity of the functional or developmental unit^4,10,11^. An integrated and modular organisation has therefore potential to affect evolutionary paths in multiple ways that include circumventing the effects of genetic pleiotropy and developmental canalisation as well as facilitating and channelling evolutionary transformations of functional and developmental units^5,12,13^.

Studies of mammalian evolution often rely on information from the dentition. Teeth are highly informative of a mammal’s taxonomic identity, phylogenetic relationships and ecological adaptation; and still constitute the most common and best-preserved mammal remains in the fossil record, adding a historical perspective to the study^14,15^. The dentition as a whole appears to be a module of the dermal exoskeleton^16^. Potential different modules within the mammalian dentition include tooth generations (milk vs permanent teeth) and tooth classes (incisors vs canines vs premolars vs molars) and can also include other groups of teeth (e.g. carnivore carnassials vs other premolars and molars)^16^. At lower levels of dental organisation, individual teeth^17^ or tooth cusps^16^ can be separate modules.

Many studies of integration and/or modularity have been conducted on complex mammalian dentitions where tooth classes are distinguishable, and teeth differ in form depending on their location in the dental arcade. These studies chiefly involved dentitions of primates^1,17–24^, carnivores^25–36^, rodents^22,37–42^ and lagomorphs^43,44^. Much less attention has been directed to simple or simplified dentitions where tooth classes are absent or not distinguishable, and teeth are similar to each other regardless of their location in the dental arcade. Notably, there has been, to our knowledge, only one study of integration and no study of modularity on a secondarily simplified dentition. This study^45^ investigated morphological integration among mandibular premolars and molars of harp seals (*Pagophilus groenlandicus*).

Pinnipeds (earless seals, Phocidae; sea lions and fur seals, Otariidae; walruses, Odobenidae) are a clade of secondarily aquatic carnivores that evolved from terrestrial ancestors with complex dentition^46–49^. Unlike their ancestors, pinnipeds forage under water where they capture, handle and swallow their prey. Prey are swallowed whole or, if too large, first torn (usually extraorally) into swallowable chunks. Pinnipeds do not masticate food but instead employ their dentition in most cases solely to catch and hold prey using a foraging style called pierce feeding^50,51^. As a likely consequence, ancestral differentiation between premolars and molars has been lost in pinnipeds. Both tooth classes are similar in size and shape (both within and between the arcades) and therefore often collectively called postcanines. Pinniped postcanines are simple or relatively simple in form, effectively two-dimensional because of the lack of a lingual cusp, and lack the refined occlusion characteristic of morphologically complex and differentiated premolars and molars in most non-pinniped (fissiped) carnivores and most mammals in general^15,52^.

The demands of functional occlusion and the process of natural selection constrain phenotypic variation and impose morphological integration in complex dentitions^53^. The simplified pinniped dentition with simple occlusion is expected to be more variable and less integrated because of relaxed functional and selective constraint. In accordance with this expectation, large intraspecific variations in tooth number have been reported from multiple pinniped species^54–64^. Furthermore, large variations in tooth size have been recorded, as expected, in ribbon seals (*Histriophoca fasciata*)^65^ and ringed seals (*Pusa hispida*)^45,65^ but, unexpectedly, not in spotted seals (*Phoca largha*), northern fur seals (*Callorhinus ursinus*) or Steller sea lions (*Eumetopias jubatus*), in all of which variations in tooth size were found to be smaller and similar to those seen in fissipeds with complex dentition and exact dental occlusion^65^. Moreover, size correlations among mandibular postcanines of *Pagophilus groenlandicus* were reported as similar to or stronger than those in fissipeds and other mammals with precisely occluding teeth^45^, suggesting an unexpectedly strong dental integration in this pinniped species. Limited size variability and strong integration are surprising in the pinniped dentition and merit further study.

In a previous paper^65^, we presented results on dental size variability in two otariid (*Eumetopias jubatus*, *Callorhinus ursinus*) and two phocid (*Phoca largha*, *Histriophoca fasciata*) species. Here, we report results on dental integration and modularity in the same species. All of these species are pierce feeders^50,66^ that feed mainly on fish (*Phoca largha*), fish and squid (*Eumetopias jubatus*, *Callorhinus ursinus*) or fish and benthic invertebrates (*Histriophoca fasciata*)^67^. Whilst these species are broadly representative of both their families and pinnipeds as a whole, which contributes to the generality of our findings, general similarities in their diets and foraging style rather do not let expect large differences in dental integration and modularity. We first measured teeth of the four species using serially homologous measurements, next calculated correlation matrices based on the collected measurement data, and then analysed correlation data in these matrices to assess the strength and structure of integration and modularity in the dentition of each species. We investigated integration at three hierarchical levels: whole dentition, among teeth and within teeth. The level of among-tooth integration included testing two classic hypotheses related to integration, the rule of neighbourhood^68,69^ and the rule of proximal parts^70^. The former states that adjacent parts of an organ are more strongly intercorrelated with respect to size than more distant parts; the latter states that proximal parts of an organ are more strongly correlated with respect to size than distal parts. We also comparatively evaluated the degree of dental occlusion among the four species to examine how integration and modularity relate to occlusion, and referred to our earlier assessment of tooth-size variability in these species^65^ to test the hypothesis that integration is negatively related to variability.

## Material and Methods

### Measurement data collection

**Figure 1:**
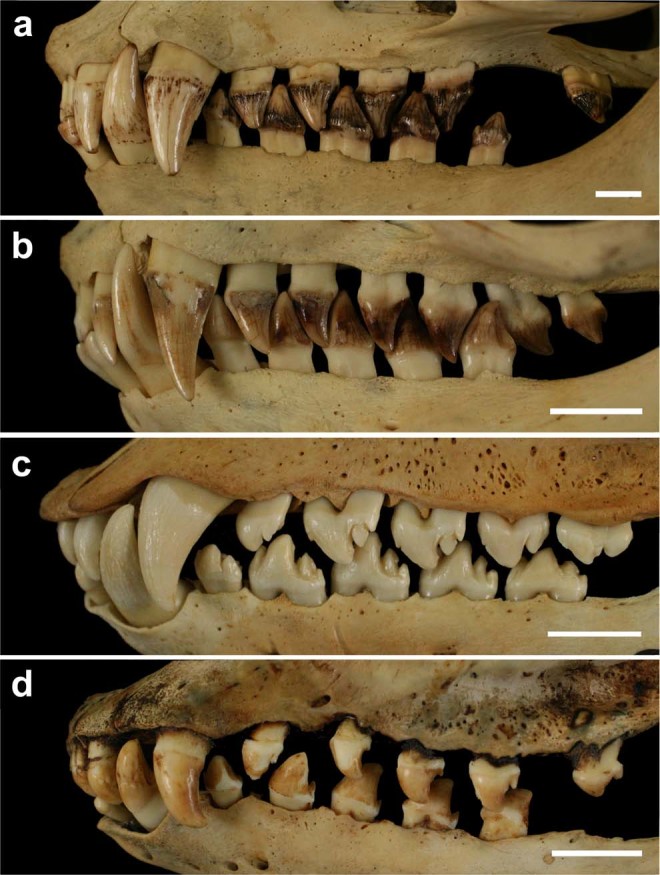
Vestibular profiles of pinniped permanent dentitions at occlusion. (**a**) *Eumetopias jubatus*, KUZ (Kyoto University Museum) M9290. (**b**) *Callorhinus ursinus*, KUZ M10142. (**c**) *Phoca largha*, KUZ M9465, reversed mirror image. (**d**) *Histriophoca fasciata*, KUZ M9575. Scale bars equal 1 cm.

Length (L; maximum linear mesiodistal distance) and width (W; maximum linear vestibulolingual distance perpendicular to the length) were measured on permanent tooth crowns in skeletonised specimens of *Eumetopias jubatus* (31 males, 30 females), *Callorhinus ursinus* (43 males, 59 females), *Phoca largha* (80 males, 60 females, 52 of undetermined gender) and *Histriophoca fasciata* (62 males, 86 females, 39 of undetermined gender). These specimens derived from wild animals on and around the Japanese Islands according to institutional collection records (Supplementary Tables S1–S4). All measurements were taken with digital calipers to the nearest 0.01 mm on one body side (left or right, depending on the state of preservation) of each specimen. Specimens with an incomplete dentition or a supernumerary tooth on both sides of the upper or lower arcade were not measured. The dental formulae of these species were I^1–3^/I_2,3_ C^1^/C_1_ P^1–4^/P_1–4_ M^1^/M_1_ for *Eumetopias jubatus*, *Phoca largha* and *Histriophoca fasciata* and I^1–3^/I_2,3_ C^1^/C_1_ P^1–4^/P_1–4_ M^1,2^/M_1_ for *Callorhinus ursinus*, where I, C, P and M denote permanent incisors, canines, premolars and molars in either half of upper and lower arcades, respectively, and superscript and subscript numbers indicate positions of upper and lower teeth, respectively (Fig. 1). Because of a difference in the number of upper molars, a total of 34 measurements were applied to *Eumetopias jubatus*, *Phoca largha* and *Histriophoca fasciata* and a total of 36 to *Callorhinus ursinus*.

### Correlation matrix calculation

Correlations were calculated using Pearson’s product-moment correlation coefficient (*r*). Measurement data were first pairwise correlated for males and females separately. Because no significant differences were observed between *r* values for males and females of each species (*P* < 0.05, Student’s *t*-tests with Holm–Bonferroni correction), specimens of both genders and those of undetermined gender were combined, and all pairwise correlations were recalculated. The *r* values resulted from these calculations were assembled into matrices, one for each species. These and all other statistical analyses were performed in r version 3.2.4 Revised^71^.

### Integration assessment

Integration was assessed using *r* entries in the correlation matrix as well as other indices directly or indirectly based on these entries and designed for a particular level of integration. High *r* values were interpreted as indicating strong integration; lower *r* values were interpreted as indicating weaker integration.

#### Whole-dentition integration

The relative standard deviation of the correlation-matrix eigenvalues, *SD*_rel_(λ)^72^, and the average of the absolute pairwise *r* values, *I*_*r*_^73^, were used to estimate the strength of overall integration. These indices were calculated with equations (1) and (2), respectively:

1 $$S{D}_{{\rm{rel}}}({\rm{\lambda }})=\sqrt{\frac{{\sum }_{i=1}^{p}{({{\rm{\lambda }}}_{i}-1)}^{2}}{p(p-1)}},$$

where λ_*i*_ denotes an eigenvalue of the correlation matrix, and *p* denotes the number of intercorrelated measurements;

2 $${I}_{r}=\frac{{\sum }_{i=1}^{k}(|{r}_{i}|)}{k},$$

where |*r*_*i*_| denotes an absolute off-diagonal *r* value in the correlation matrix, and *k* denotes the number of these values. Both indices are independent of the sample size or the number of intercorrelated measurements and vary between zero (no integration) and one (perfect integration), with the *I*_*r*_ index tending to yield lower values than those of the *SD*_rel_(λ) index^72,74^.

#### Among-tooth integration

Correlation matrix *r* values were used to test the rules of neighbourhood and proximal parts and to assess the strength of integration between teeth. The relative strength and the structure of integration among teeth were analysed with hierarchical unweighted pair-group average (UPGMA) clustering using the average of absolute pairwise *r* values between measurements of two different teeth (*r*_M_) subtracted from one as a dissimilarity measure. The *r*_M_ metric was calculated, using a pair of upper and lower canines as an example, as the sum of *r* values between LC^1^ and LC_1_, between LC^1^ and WC_1_, between WC^1^ and LC_1_, and between WC^1^ and WC_1_ divided by four. Clustered teeth were interpreted as more strongly integrated than non-clustered ones.

#### Within-tooth integration

The strength of integration within teeth was estimated using the absolute *r* value between measurements of the same tooth. Species-specific patterns of within-tooth integration were identified by plotting these *r* values along the arcade.

### Modularity assessment

The potential modular structure of the dentition was analysed by hierarchical UPGMA clustering of teeth using a dissimilarity measure of 1 − *r*_M_. Potential modules were expected to be identified by clusters. We additionally assumed that tooth classes could be modules as expected for a mammal’s dentition^75,76^. All hypothesised modules (whether identified or assumed) were next tested using the covariance ratio (*CR*)^77^ and Escoufier’s^78^ *RV* coefficient^79^. Statistical significance of these coefficients was assessed using 9999 iterations of the permutation procedure as described in ref.^77^ (*CR*) and ref.^79^ (*RV*). Both coefficients were also used to estimate the strength of modularity. The *RV* coefficient ranges from zero (perfect modularity) to one (no modularity)^79^. The *CR* coefficient ranges from zero to positive values: the *CR* values between zero and one imply a modular structure, with low values corresponding to relatively more modularity, and higher values corresponding to relatively less modularity; the *CR* values higher than one imply no modularity^77^. The *CR* coefficient is unaffected by the sample size or the number of intercorrelated measurements^77^, whereas the *RV* coefficient has been shown to be sensitive to both^77,80,81^. Despite this bias, we used the *RV* coefficient because it has commonly been applied to quantify morphological modularity, and to check whether both coefficients converge on similar results. Assessment of modularity was supplemented by observations of the shape of adjacent teeth within and between hypothesised modules, assuming that teeth are similar in form within a module and different between modules.

### Occlusion evaluation

The relative degree of dental occlusion among species was qualitatively evaluated using four criteria: the number of teeth lacking occlusal contact with opposing teeth, the number of wear facets on the crowns, the size of these facets relative to the size of the crown, and the size of spaces between adjacent teeth of the same arcade. These criteria were interpreted such that fewer non-contacting teeth, more and larger wear facets and smaller interdental spaces indicated relatively more occlusion, whereas more non-contacting teeth, fewer and smaller wear facets and larger interdental spaces indicated relatively less occlusion.

## Results

### Integration

**Figure 2:**
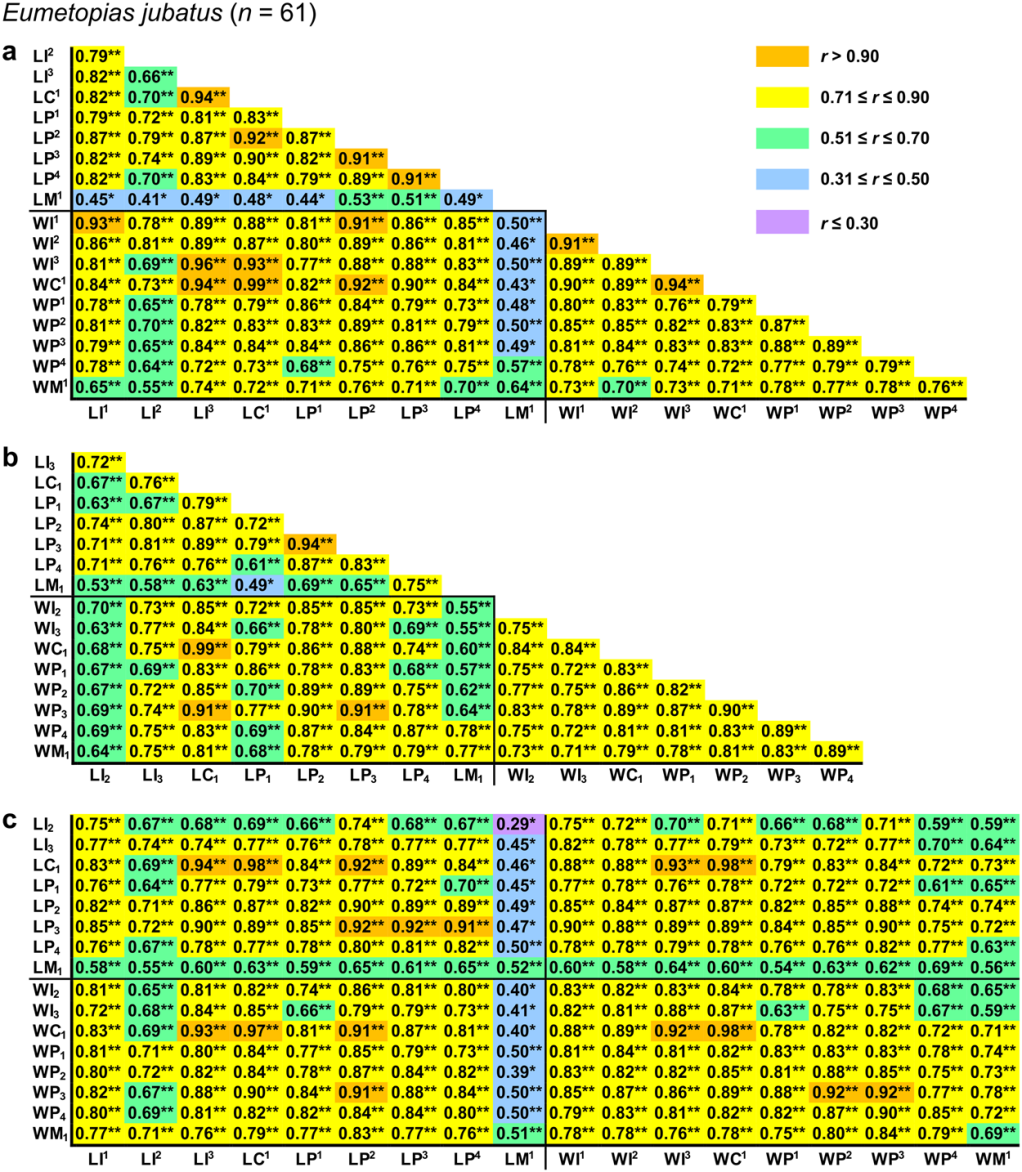
Correlation matrix for measurements in *Eumetopias jubatus*. The matrix is visualised in three parts that contain correlations among upper (**a**) or lower (**b**) teeth and between upper and lower teeth (**c**). Symbols and abbreviations: *n*, number of individuals; *r*, Pearson’s product-moment correlation coefficient; L, mesiodistal length of the tooth crown; W, vestibulolingual width of the tooth crown; I, incisor; C, canine; P, premolar; M, molar; superscript and subscript numbers denote positions of upper and lower teeth, respectively. Asterisks indicate *r* values that are statistically significant (*P ≤ 0.05, **P ≤ 0.001; Student’s *t*-test with Holm–Bonferroni correction). Descriptive statistics for the measurements are in Supplementary Table S5.

**Figure 3:**
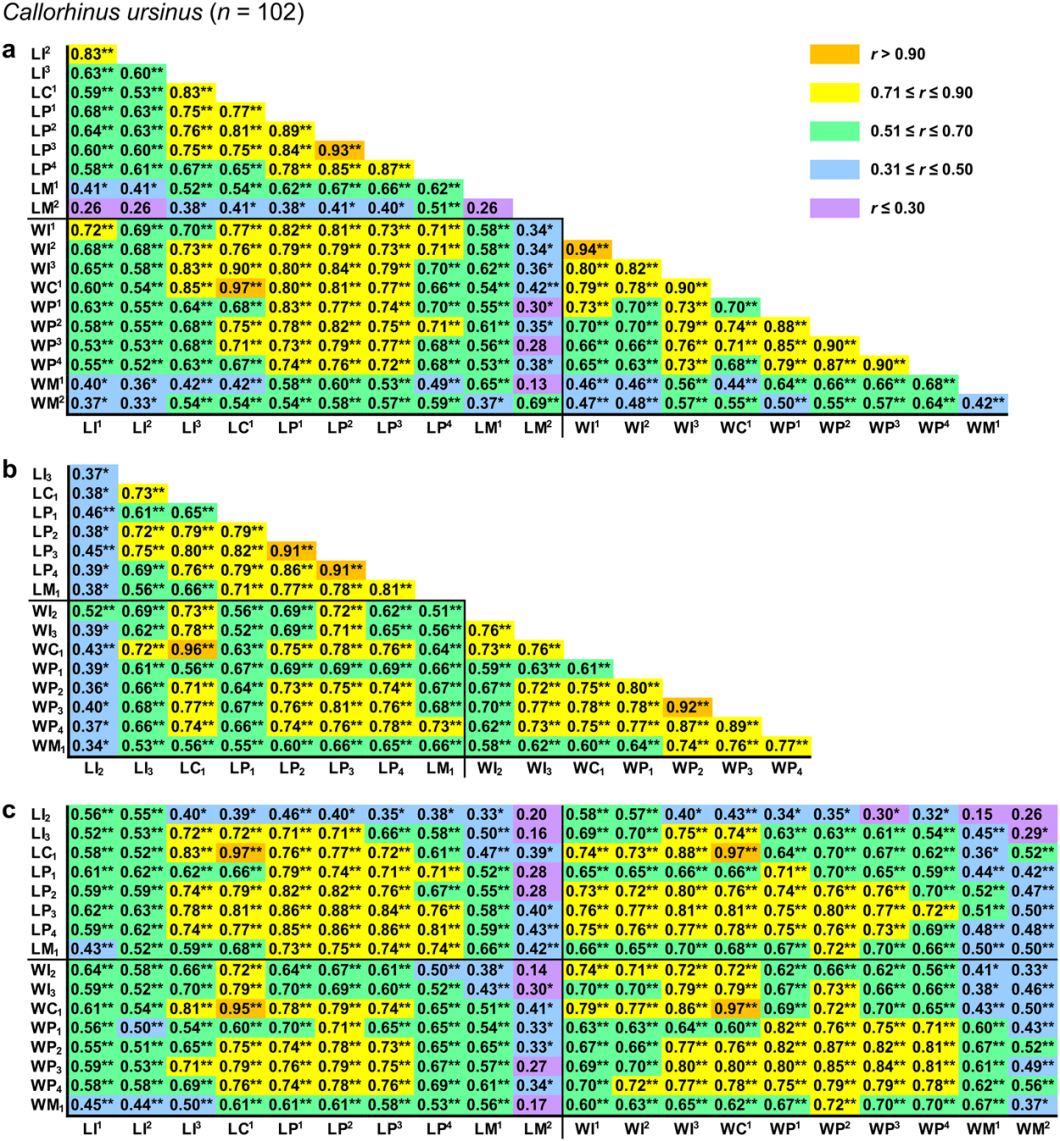
Correlation matrix for measurements in *Callorhinus ursinus*. The matrix is visualised in three parts that contain correlations among upper (**a**) or lower (**b**) teeth and between upper and lower teeth (**c**). Symbols and abbreviations: *n*, number of individuals; *r*, Pearson’s product-moment correlation coefficient; L, mesiodistal length of the tooth crown; W, vestibulolingual width of the tooth crown; I, incisor; C, canine; P, premolar; M, molar; superscript and subscript numbers denote positions of upper and lower teeth, respectively. Asterisks indicate *r* values that are statistically significant (*P ≤ 0.05, **P ≤ 0.001; Student’s *t*-test with Holm–Bonferroni correction). Descriptive statistics for the measurements are in Supplementary Table S6.

**Figure 4:**
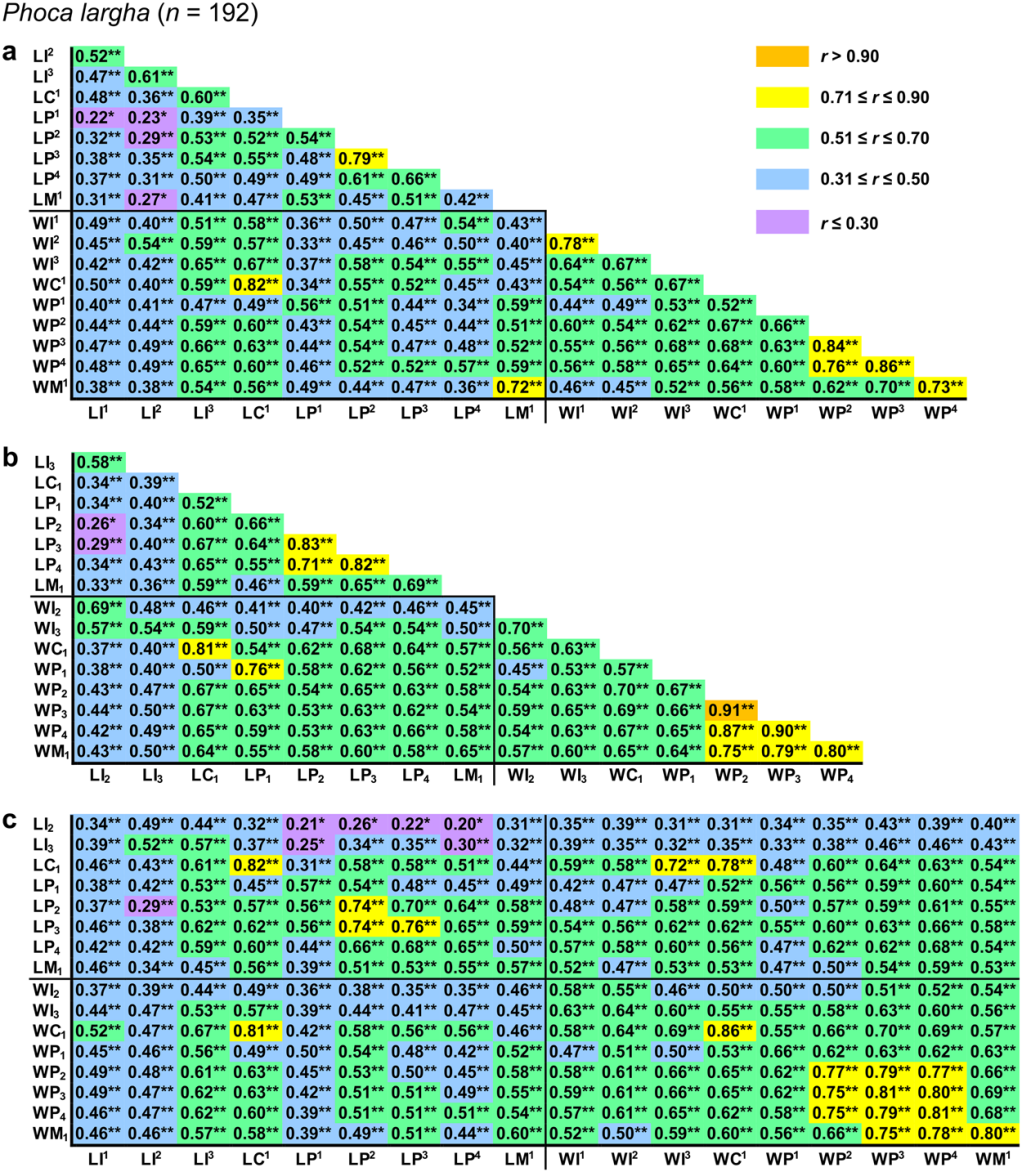
Correlation matrix for measurements in *Phoca largha*. The matrix is visualised in three parts that contain correlations among upper (**a**) or lower (**b**) teeth and between upper and lower teeth (**c**). Symbols and abbreviations: *n*, number of individuals; *r*, Pearson’s product-moment correlation coefficient; L, mesiodistal length of the tooth crown; W, vestibulolingual width of the tooth crown; I, incisor; C, canine; P, premolar; M, molar; superscript and subscript numbers denote positions of upper and lower teeth, respectively. Asterisks indicate *r* values that are statistically significant (*P ≤ 0.05, **P ≤ 0.001; Student’s *t*-test with Holm–Bonferroni correction). Descriptive statistics for the measurements are in Supplementary Table S7.

**Figure 5:**
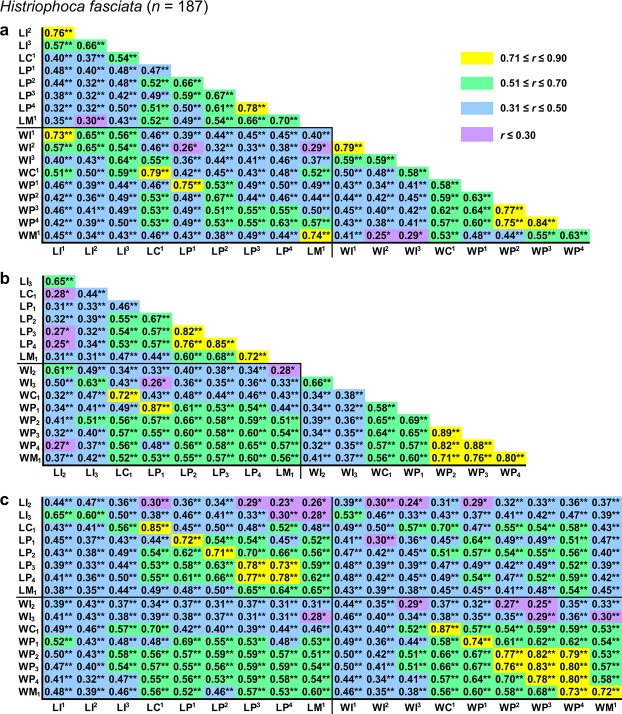
Correlation matrix for measurements in *Histriophoca fasciata*. The matrix is visualised in three parts that contain correlations among upper (**a**) or lower (**b**) teeth and between upper and lower teeth (**c**). Symbols and abbreviations: *n*, number of individuals; *r*, Pearson’s product-moment correlation coefficient; L, mesiodistal length of the tooth crown; W, vestibulolingual width of the tooth crown; I, incisor; C, canine; P, premolar; M, molar; superscript and subscript numbers denote positions of upper and lower teeth, respectively. Asterisks indicate *r* values that are statistically significant (*P ≤ 0.05, **P ≤ 0.001; Student’s *t*-test with Holm–Bonferroni correction). Descriptive statistics for the measurements are in Supplementary Table S8.

All pairwise *r* values among measurements were positive and statistically significant except 14 (2.2%) values that were insignificant in *Callorhinus ursinus* (Figs 2–5).

#### Whole-dentition integration

Pairwise *r* values among measurements were in most cases higher in both otariid species than in either phocid species, with *Eumetopias jubatus* generally showing the highest values and *Histriophoca fasciata* the lowest (Figs 2–5). Consistent with this observation, as expected, were values of integration indices, *SD*_rel_(λ) and *I*_*r*_, which were, respectively, 0.776 and 0.767 for *Eumetopias jubatus*, 0.660 and 0.643 for *Callorhinus ursinus*, 0.549 and 0.535 for *Phoca largha*, and 0.510 and 0.500 for *Histriophoca fasciata*. These results indicated the strongest overall integration in *Eumetopias jubatus*, followed in descending order by those in *Callorhinus ursinus*, *Phoca largha* and *Histriophoca fasciata*.

#### Among-tooth integration

Measurements of teeth that occluded with each other tended to be more strongly intercorrelated than those of upper vs lower teeth that did not occlude in each of the four species evaluated (Figs 2c, 3c, 4c and 5c; *P* < 0.018, Mann–Whitney *U*-tests), indicating stronger integration between occluding teeth compared to that between non-occluding ones. Furthermore, as predicted by the rule of neighbourhood, measurements of adjacent teeth of an arcade tended to be more strongly intercorrelated than those of more distant teeth of that arcade in each of the four species (Figs 2a,b, 3a,b, 4a,b and 5a,b; *P* < 0.033, Mann–Whitney *U*-tests), which indicated a tendency for stronger integration between adjacent teeth of the same arcade compared to that between non-adjacent ones. However, contrary to the rule of proximal parts, measurements of more mesial teeth of both arcades tended not to be more strongly intercorrelated than those of more distal teeth of both arcades in each of the four species (Figs 2–5; *P* = 0.13–0.71, tests for the significance of correlation between the *r* coefficient and the position of the tooth pair using Student’s *t*-distribution), which indicated that integration did not tend to be stronger between more mesial teeth compared to that between more distal teeth.

**Figure 6:**
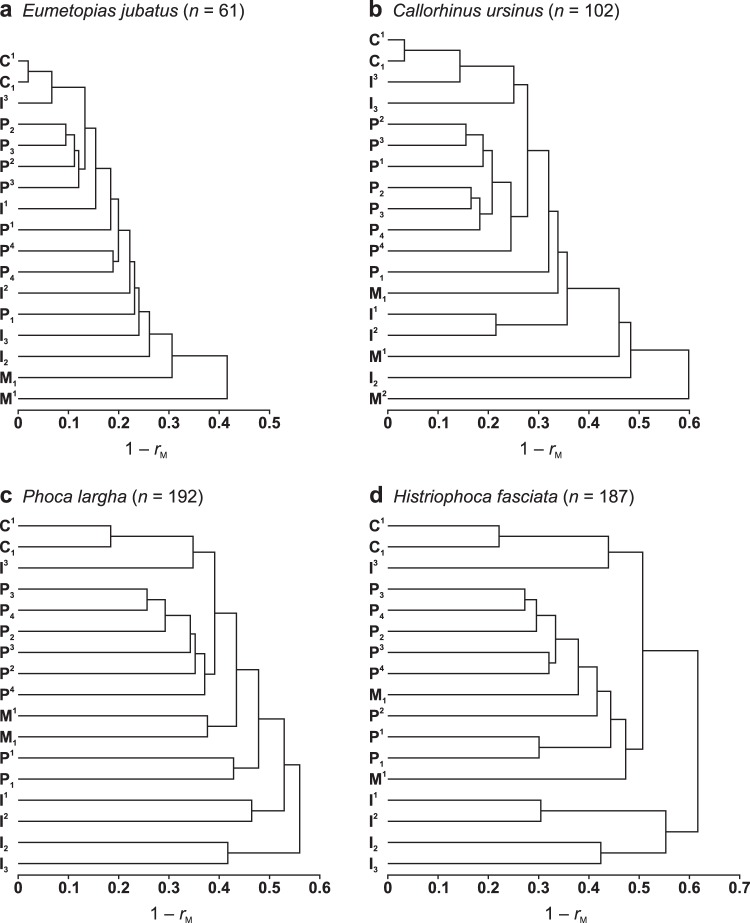
Hierarchical UPGMA clustering of teeth based on the average correlation between their measurements in *Eumetopias jubatus* (**a**), *Callorhinus ursinus* (**b**), *Phoca largha* (**c**) and *Histriophoca fasciata* (**d**). Symbols and abbreviations: *n*, number of individuals; *r*_M_, arithmetic mean of pairwise Pearson’s product-moment correlation coefficients between measurements of two different teeth; I, incisor; C, canine; P, premolar; M, molar; superscript and subscript numbers indicate positions of upper and lower teeth, respectively.

Measurements of C^1^ and C_1_ were more strongly intercorrelated than those of any other teeth in all four species evaluated and especially in both otariid species (Figs 2–6), which indicated the strongest integration between the canines. Canine measurements were most strongly correlated with those of I^3^ in all of the four species and especially in both otariid species (Figs 2–6), indicating strong integration among C^1^, C_1_ and I^3^. Measurements of postcanines that corresponded in position to the carnassials in fissipeds (P^4^ and M_1_) were relatively weakly intercorrelated in all the four species (Figs 2c, 3c, 4c, 5c and 6), indicating a relatively weak integration between these teeth. The most distal upper postcanines of both otariid species (M^1^ of *Eumetopias jubatus* and M^2^ of *Callorhinus ursinus*) were positioned separately from all other teeth in the respective dendrograms resulted from cluster analysis (Fig. 6a,b), and their measurements tended to be most weakly correlated with those of other teeth (Figs 2a,c and 3a,c; *P* < 0.0001, Mann–Whitney *U*-tests), indicating the weakest integration with other teeth of the dentition. In contrast, the most distal upper postcanine of either phocid species (M^1^) was not positioned separately from all other teeth in the respective dendrograms resulted from cluster analysis (Fig. 6c,d), and its measurements were relatively strongly correlated with those of other teeth (Figs 4a,c and 5a,c; Mann–Whitney *U*-tests did not reject the null hypothesis of M^1^ measurements being not most weakly correlated with those of other teeth, with *P* = 0.89 for *Phoca largha* and *P* = 0.93 for *Histriophoca fasciata*), indicating a relatively strong integration of M^1^ with other teeth of the dentition.

#### Within-tooth integration

**Figure 7:**
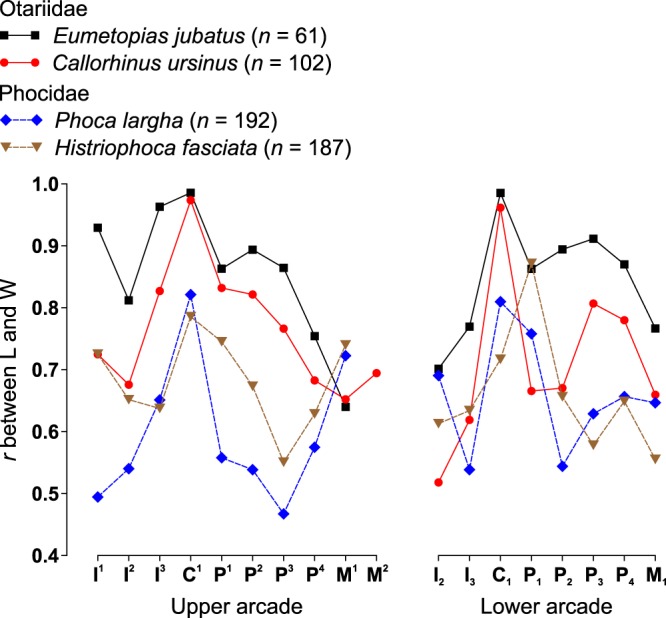
Correlation between measurements of the same tooth along the arcades in four pinniped species. Symbols and abbreviations: *n*, number of individuals; *r*, Pearson’s product-moment correlation coefficient; L, mesiodistal length of the tooth crown; W, vestibulolingual width of the tooth crown; I, incisor; C, canine; P, premolar; M, molar; superscript and subscript numbers indicate positions of upper and lower teeth, respectively.

A comparison of *r* values between measurements of the same tooth along the upper and lower arcades of each evaluated species revealed patterns of within-tooth integration. These patterns were more similar between both otariid species than between both phocid species and differed between the otariid and phocid species (Fig. 7). The canines were the most strongly internally integrated teeth of their arcades in all of the four species evaluated except for the *Histriophoca fasciata* lower arcade where P_1_ was more strongly integrated internally than C_1_ (Fig. 7). The internal integration of C^1^ was stronger than that of C_1_ in all of the four species, and both were very strong in both otariid species and weaker in both phocid species (Fig. 7). The P^4^ and M_1_ of all the four species as well as M^1^ of *Eumetopias jubatus* and M^2^ of *Callorhinus ursinus* were relatively weakly integrated internally, whereas M^1^ in both phocid species was relatively strongly integrated internally (Fig. 7).

### Modularity

Cluster analyses identified a potential module composed of C^1^, C_1_ and I^3^ in all four species evaluated but did not reveal a distinct modular structure in the whole dentition of any of these species (Fig. 6). In turn, analyses of the *CR* and *RV* coefficients (both coefficients mostly provided congruent results) supported a modular nature of the canine–I^3^ complex in both phocid species and, to a lesser extent, in *Callorhinus ursinus* but not in *Eumetopias jubatus* (Table 1). In addition, contrary to the cluster analyses, results of the *CR* and *RV* analyses generally implied a modular structure with tooth classes as modules in both phocid species and, to a lesser extent, in both otariid species, although all *CR* and most *RV* values were high (closer to one than to zero), which indicated that the modular structure was weak (Table 1). All *CR* and most *RV* values for comparisons of the molars with either the premolars only or the premolars combined with the canines and the incisors were higher for both phocid species than for either otariid species, indicating the lesser distinctiveness of molars from the rest of the dentition in these phocid species (Table 1). The *CR* and *RV* values for other comparisons between groups of teeth were in most cases lowest in *Histriophoca fasciata*, followed in ascending order by those in *Phoca largha*, *Callorhinus ursinus* and *Eumetopias jubatus* (Table 1). This order of species was exactly opposite to that according to increasing *SD*_rel_(λ) and *I*_*r*_ values for whole-dentition integration, indicating a negative relationship between the degrees of modularity and integration.

**Table 1:** Degree of modularity between groups of teeth in four pinniped species.

Groups of teeth	Otariidae								Phocidae							
	*Eumetopias jubatus* (*n* = 61)				*Callorhinus ursinus* (*n* = 102)				*Phoca largha* (*n* = 192)				*Histriophoca fasciata* (*n* = 187)			
	*CR*	*P*_*CR*_	*RV*	*P*_*RV*_	*CR*	*P*_*CR*_	*RV*	*P*_*RV*_	*CR*	*P*_*CR*_	*RV*	*P*_*RV*_	*CR*	*P*_*CR*_	*RV*	*P*_*RV*_
Canines and I^3^ vs all other teeth	1.016	0.006	0.865	0.182	**0.957**	0.012	0.730	0.188	**0.905**	<0.001	**0.629**	0.007	**0.832**	<0.001	**0.527**	<0.001
Canines vs all other teeth	1.060	0.040	0.904	0.465	0.999	0.054	0.779	0.477	**0.907**	<0.001	**0.611**	0.047	**0.836**	0.002	**0.513**	0.006
Incisors vs all other teeth	1.378	0.800	0.976	0.318	1.574	0.915	0.961	0.612	1.077	0.694	0.824	0.345	1.047	0.286	0.793	0.179
Premolars vs all other teeth	1.019	0.004	**0.866**	0.007	**0.940**	<0.001	**0.719**	0.005	**0.948**	<0.001	**0.711**	<0.001	**0.933**	0.002	**0.676**	<0.001
Molars vs all other teeth	**0.938**	<0.001	**0.525**	<0.001	**0.907**	<0.001	**0.429**	<0.001	**0.987**	0.013	**0.604**	0.041	**0.999**	0.050	0.618	0.076
Canines vs incisors	1.143	0.056	0.910	0.162	1.100	0.044	0.831	0.186	**0.968**	0.029	0.599	0.117	**0.828**	0.008	**0.444**	0.019
Canines vs premolars	1.029	0.001	0.854	0.248	**0.937**	0.005	0.687	0.168	**0.886**	<0.001	**0.569**	<0.001	**0.804**	<0.001	**0.468**	<0.001
Canines vs molars	**0.940**	0.027	**0.497**	0.013	**0.894**	0.020	0.393	0.052	**0.896**	0.028	**0.428**	0.031	**0.841**	0.031	**0.372**	0.028
Incisors vs premolars	1.055	0.084	**0.871**	<0.001	1.006	<0.001	**0.795**	<0.001	**0.914**	0.002	**0.600**	<0.001	**0.736**	<0.001	**0.404**	<0.001
Incisors vs molars	**0.988**	0.001	**0.533**	0.012	**0.928**	<0.001	**0.426**	0.011	**0.905**	0.003	**0.434**	0.009	**0.732**	0.002	**0.291**	0.001
Premolars vs molars	**0.996**	<0.001	**0.626**	<0.001	**0.989**	0.003	**0.550**	0.002	0.999	0.054	**0.600**	0.009	1.010	0.065	**0.620**	0.013
Canines vs incisors vs premolars vs molars	1.025	<0.001	**0.715**	<0.001	**0.976**	<0.001	**0.614**	0.007	**0.928**	<0.001	**0.538**	<0.001	**0.825**	<0.001	**0.433**	<0.001

Symbols and abbreviations: *n*, number of individuals; *CR*, covariance ratio; *RV*, Escoufier’s *RV* coefficient; *P*_*CR*_, permutation *P* value for the *CR* coefficient; *P*_*RV*_, permutation *P* value for the *RV* coefficient; I^3^, third upper incisor. Bold face values of the *CR* (*P*_*CR*_ ≤ 0.05) and *RV* (*P*_*RV*_ < 0.05) coefficients imply a valid modular structure.

These results were congruent with and extended by observations that I^3^ closely resembled C^1^ in form in all four species evaluated, and that teeth were serially similar except relative discontinuities between C_1_ and P_1_ in all of the species, between C^1^ and P^1^ in both phocid species, and between P^4^ and M^1^ in both otariid species (Fig. 1). These observations indicated that the molars are more distinctive from the premolars in the upper arcade than in the lower one in both otariid species.

### Occlusion

A comparison of the degree of dental occlusion showed that overall occlusion was more extensive in both otariid species than in either phocid species, and that it was least pronounced in *Histriophoca fasciata* in which spaces between adjacent postcanines of the same arcade were largest relative to postcanine size, and the opposing upper and lower postcanines often did not come into occlusal contact with each other (Fig. 1). Wear facets on postcanine crowns were larger relative to the size of the crown and occurred more often in *Eumetopias jubatus* than in *Callorhinus ursinus*, indicating a more extensive occlusion in the former species. These observations indicated the highest degree of dental occlusion in *Eumetopias jubatus*, followed by those in *Callorhinus ursinus*, *Phoca largha* and *Histriophoca fasciata*, in this descending order, thus matching the order of these species according to weakening whole-dentition integration and increasing modularity.

Regarding the most distal upper postcanines, M^1^ of *Eumetopias jubatus* and M^2^ of *Callorhinus ursinus* lacked occlusal contact with teeth of the lower arcade (Fig. 1a,b), M^1^ of *Phoca largha* occluded with M_1_ (Fig. 1c), and M^1^ of *Histriophoca fasciata* was variable. It occluded with M_1_ in some specimens but was deprived of any contact in others (Fig. 1d).

## Discussion

This study found that dental integration was positively related to dental occlusion across four representative pinniped species, and that integration was stronger between occluding teeth than between non-occluding ones in each of these species. A comparison with our previous findings on tooth-size variation in the same species^65^ shows that dental integration and occlusion are roughly negatively related to dental size variability, with the most integrated and occluding dentition being the least variable (*Eumetopias jubatus*) and the least integrated and occluding dentition the most variable (*Histriophoca fasciata*). This concurs with the expectation that the degree of integration is related positively to the degree of occlusion and negatively to the degree of variability, providing a functional rationale for many differences in dental integration and dental size variability among the four species. This also indicates that functional requirements of occlusion significantly contribute to integration in the pinniped dentition despite the fact that both the postcanines and occlusion are considerably simplified in this dentition compared to those in the complex dentition of most other mammals. This conclusion is further supported by our observations from the canines, I^3^, P^4^, M_1_ and the most distal upper postcanines.

The primary role of the canines in mammals is to serve as occlusal guides for the postcanines^82^, a function that is a plausible candidate to account for the strong integration observed between and within the canines in the four pinniped species. The strong integration among the canines and I^3^ and the likely modular nature of the canine–I^3^ complex found in this study suggest that I^3^ may also be involved in this function in all of the four species. A positive relationship between the degrees of canine–I^3^ integration and dental occlusion (both were highest in *Eumetopias jubatus* and decreased, in descending order, in *Callorhinus ursinus*, *Phoca largha* and *Histriophoca fasciata*) supports this functional interpretation. The strong internal integration of P_1_ relative to that of C_1_ observed in *Histriophoca fasciata* and *Phoca largha* suggests that P_1_ might be an additional element of this functional complex in these phocid species, but the outcomes of cluster analysis contradicted this hypothesis by showing that the measurements of P_1_ were most strongly correlated with those of P^1^ and that the P_1_–P^1^ cluster was far from the canine–I^3^ cluster in both phocid species.

Another potential influence on integration of the canines derives from the fact that males of many pinniped species use their canines in combat over territory and females. However, whilst this behaviour holds true for *Eumetopias jubatus* and *Callorhinus ursinus*, which mate on land, it does not hold for *Phoca largha* and *Histriophoca fasciata*, which mate in the water where there is no need for the male to defend territory or compete for females by trying to dominate other males^83^. Moreover, we observed no significant differences between canine *r* values of males and females for each of the four species, which suggests that male-to-male combat behaviour does not importantly affect the canine integration. Interestingly, the canines were considerably sexually dimorphic in both otariid species and larger relative to other teeth than those in both phocid species^65^, which is apparently because of the difference in mating systems^84^.

Unlike fissiped carnassials, which are rather strongly integrated relative to other teeth of the dentition^25,27–31^, their positional counterparts in the four pinniped species (P^4^ and M_1_) were relatively weakly integrated both with each other and within themselves, which is expected from a functional standpoint because these teeth lost their carnassial function early in pinniped evolution^51,85^. Furthermore, the most distal upper postcanines of both otariid species exhibited the weakest integration with other teeth of the dentition and a relatively weak internal integration as well as a considerable size variation^65^; which was in contrast to the most distal upper postcanines of both phocid species, which exhibited a relatively strong integration with other teeth of the dentition and a strong internal integration as well as a size variation comparable to that of other teeth of the dentition^65^. This is also expected from a functional standpoint because the most distal upper postcanines of both otariid species lacked occlusal contact with teeth of the lower arcade, whereas the most distal upper postcanines of both phocid species invariably or variably occluded with a tooth of the lower arcade. The situation in these otariids is comparable to that in fissipeds where the most distal teeth that show no or little occlusion are less integrated and more variable than other teeth^25–27,29,31,86,87^.

Our study also revealed evidence showing that developmental factors play an important role in shaping integration in the pinniped dentition. Specifically, a modular structure with tooth classes as modules, albeit weak, was identified. Moreover, our results generally concurred with previous findings regarding the validity of the rules of neighbourhood and proximal parts in the case of both a whole dentition and a series of teeth representing more than one tooth class^19,25–27,29,31,34,45^, indicating that not only complex mammalian dentitions but also secondarily simplified pinniped dentitions generally hold to the rule of neighbourhood but not to the rule of proximal parts. Adherence to a modular structure among tooth classes and to the rule of neighbourhood are expected in a mammal’s dentition from a developmental point of view given that developmental histories can be common within but different between tooth classes (e.g. premolars vs molars, the former having two generations and the latter only having one), and that teeth are considered developmentally interrelated metameric members of a serially homologous meristic series^75,88–90^, and adjacent tooth buds or teeth physically contact each other along the dental lamina or arcade during ontogeny and can also otherwise influence each other (e.g. the first molar to develop can determine the size of the successive ones^91,92^).

A comparison of our results from four pinniped species (*I*_*r*_ = 0.500–0.767) with values of this index calculated from previously reported dental correlation matrices for mammal species with complex dentition^20,21,23,25,27–29,31,34,35,37,43,44^ (*I*_*r*_ = 0.291–0.683) shows that dental integration in these pinniped species with simple dental occlusion is stronger than or similar to that in mammal species with refined occlusion. This is surprising when viewed from a solely functional perspective. We propose that both functional factors related to dental occlusion and developmental factors related to modularity have contributed to the strong integration in the pinnipeds in this study. Specifically, modularity was found in this study to be weak and negatively related to integration. Both are not surprising given reduced heterodonty in the pinnipeds examined, and the fact that modules require no or weak intermodular integration to exist, which constrains overall integration of a structure composed of modules. We hypothesise that high levels of modularity in complex mammalian dentitions^17,22–24,36,40,42^ effectively constrain overall integration to moderate levels, whilst the lower levels of modularity revealed in the simplified pinniped dentitions in this study enable the higher levels of overall integration. We further hypothesise that the potential high levels of integration enabled by reduced modularity have effectively been achieved in these pinnipeds in response to selective pressure driven by functional requirements of dental occlusion, which, albeit weak in these pinnipeds, positively influences dental integration.

It has been suggested that evolutionarily conserved developmental programmes for the mammalian dentition underlie integration in the pinniped dentition^45^. Whilst the weak tooth-class modules identified in our study are apparently the remnant from a conserved ancestral mammalian pattern, we propose that the decisive developmental programme is an evolutionary novelty that arose in pinnipeds during the transition from terrestrial to aquatic life in association with the origin of pierce feeding and loss of mastication driven by functional requirements of underwater feeding. The simplification of tooth form and increased mutual similarity of teeth representing different classes are apparently associated with reduced dental modularity, and together with increased tooth spacing that is associated with decreased postcanine size^51,66^, they are likely manifestations of adaptation to underwater feeding. Developmental processes that lie behind these changes in early pinnipeds likely converge to some extent with those hypothesised for cetaceans^93^.

The greater disparity in patterns of within-tooth integration between phocid species than between otariid species found in our study suggests a greater diversification of integration patterns in Phocidae than in Otariidae. A comparison of our results from four representative pierce feeding species with correlation data from mandibular postcanines of another pierce feeding species, *Pagophilus groenlandicus*^45^ (*I*_*r*_ = 0.587), suggests that high levels of dental integration are common among pierce feeders, and we expect other pinnipeds (both suction feeders and filter feeders^50^) to show similarly high levels provided that there is a functional factor that drives integration in their dentition. If there is no functional factor, we expect a rather weak integration. Our findings indicate that this factor is dental occlusion in pierce feeders. Exploration of suction and filter feeding pinnipeds is needed to determine whether their dental integration is weak or strong and, in the latter case, to identify the functional factor that drives the integration.

## Supplementary information

Supplementary Information

## Data Availability

Measurement data analysed in this study are available in Supplementary Tables S1–S4.
