# Supplementary material for: Dental integration and modularity in pinnipeds
Source: Sci Rep. 2019 Mar 12;9:4184. doi: 10.1038/s41598-019-40956-1 (PMC6414606; doi:10.1038/s41598-019-40956-1)
Supplement: Supplementary file 1 — Supplementary Information [file 41598_2019_40956_MOESM1_ESM.pdf]

Dental integration and modularity in pinnipeds

Mieczysław Wolsan, Satoshi Suzuki, Masakazu Asahara, Masaharu Motokawa

SUPPLEMENTARY INFORMATION
(Tables S1–S8)

Table S1. Measurement values for *Eumetopias jubatus*. All values are in millimeters. Measurement abbreviations: L, length of the tooth crown (maximum linear mesiodistal distance across the tooth crown); W, width of the tooth crown (maximum linear vestibulolingual distance across the tooth crown perpendicular to the length); I, incisor; C, canine; P, premolar; M, molar; superscript and subscript numbers indicate positions of upper and lower teeth, respectively. Institutional abbreviations: HUM, Hokkaido University Museum, Hokkaido University, Sapporo, Japan; HUNHM, Botanic Garden, Hokkaido University, Sapporo, Japan; KUZ, Kyoto University Museum, Kyoto University, Kyoto, Japan; NSMT, National Museum of Nature and Science, Tokyo, Japan.

Specimen reference No.	Measurements																																	
	LI^1	WI^1	LI^2	WI^2	LI^3	WI^3	LC^1	WC^1	LP^1	WP^1	LP^2	WP^2	LP^3	WP^3	LP^4	WP^4	LM^1	WM^1	LI_2	WI_2	LI_3	WI_3	LC_1	WC_1	LP_1	WP_1	LP_2	WP_2	LP_3	WP_3	LP_4	WP_4	LM_1	WM_1
**Males**																																		
HUM 1	3.88	6.34	4.96	8.35	12.57	16.26	21.65	18.90	10.75	8.84	13.06	10.38	13.33	10.34	13.17	9.32	13.55	7.21	5.01	6.02	9.11	6.90	23.10	15.06	10.33	7.63	12.48	8.57	14.42	9.43	13.80	8.46	9.96	6.68
HUM 3	3.87	6.42	4.89	8.05	12.34	14.67	21.75	18.71	11.41	8.93	13.69	10.31	13.48	10.41	12.97	8.66	11.20	6.55	5.02	5.91	9.40	6.77	22.04	15.20	10.45	7.68	12.48	8.58	14.60	9.08	14.02	8.92	11.12	6.98
HUM 4	3.72	6.10	5.18	7.12	10.86	13.60	20.68	19.20	11.39	8.14	13.64	10.03	12.98	9.60	13.43	8.33	11.76	6.81	4.83	6.22	9.72	6.70	23.90	16.70	10.40	6.98	12.61	8.32	14.08	9.13	13.34	8.89	11.32	7.14
HUM 6	3.52	5.93	4.72	7.41	11.87	14.86	20.83	17.77	10.72	8.49	13.12	9.39	12.64	9.92	12.47	8.50	10.62	7.24	5.67	5.82	9.16	6.17	22.39	15.41	10.04	7.46	12.16	7.79	14.02	9.24	13.09	8.56	10.89	6.49
HUM 13	3.79	6.23	5.50	7.27	10.84	12.95	21.55	18.72	11.00	8.21	13.13	9.69	12.33	9.38	12.39	8.52	10.62	6.40	6.04	5.62	9.73	7.04	21.68	15.00	10.12	7.65	12.03	8.42	13.58	8.79	13.22	8.25	10.40	7.07
HUM 16	4.02	6.16	4.63	7.47	11.60	14.94	22.10	19.86	11.17	8.82	13.42	10.88	13.30	10.86	12.81	9.27	11.29	6.81	5.98	6.81	9.26	7.11	23.84	15.14	10.08	7.47	12.72	7.99	13.83	9.74	14.02	9.63	10.73	6.76
HUM 21	3.46	5.86	4.65	7.40	11.30	15.45	21.92	19.85	9.26	7.18	12.80	9.38	12.53	9.23	11.82	8.27	10.56	6.38	4.91	5.43	9.08	8.76	23.09	16.38	8.92	7.02	11.52	7.91	13.26	8.56	11.58	8.05	10.16	6.62
HUM 22	3.52	5.72	4.18	7.04	12.17	14.19	20.69	17.01	10.82	8.21	11.98	9.44	12.43	9.64	12.30	8.16	9.63	6.04	5.13	5.43	9.28	6.63	20.06	13.76	10.30	7.05	11.79	7.13	13.74	8.78	12.38	8.01	10.02	6.01
HUM 31	3.55	5.74	4.58	6.92	11.46	14.49	25.00	20.24	11.68	9.06	13.29	9.77	12.89	9.83	12.05	8.05	12.03	6.68	5.03	5.87	8.78	6.40	24.74	16.50	10.22	7.63	12.02	7.80	13.70	9.06	12.97	8.54	10.92	6.55
KUZ M9286	4.19	6.77	5.45	8.27	12.00	15.20	27.26	22.89	12.66	9.55	15.16	11.10	14.49	11.49	14.20	10.17	12.82	7.67	5.67	6.11	10.76	7.15	28.98	18.73	11.57	8.70	12.80	8.74	15.53	10.30	14.60	10.10	11.50	7.95
KUZ M9436	3.60	5.80	4.31	7.84	11.75	14.83	21.46	18.10	10.67	8.58	13.04	10.36	12.24	10.04	12.52	8.38	10.78	6.79	5.09	5.62	8.36	6.60	24.11	17.46	10.95	7.90	11.90	8.35	14.21	9.52	12.42	8.59	10.42	6.87
KUZ M9438	3.43	5.40	4.30	6.85	11.67	12.75	19.42	16.86	10.50	8.07	12.23	9.55	12.55	9.91	11.72	8.69	10.83	6.69	4.66	5.27	8.33	6.38	22.71	14.83	9.14	6.85	11.66	8.10	12.98	9.00	12.12	8.54	10.01	6.85
KUZ M9581	4.02	6.27	4.70	7.84	12.17	15.48	22.96	19.60	12.20	9.41	13.87	10.95	12.93	10.93	12.94	9.36	11.24	7.40	5.26	6.30	10.16	7.23	27.53	18.10	11.29	8.70	13.03	9.30	15.08	10.31	12.57	9.78	11.44	7.33
KUZ M9586	4.03	6.51	4.89	7.90	11.48	14.57	21.24	18.84	10.56	8.90	13.29	10.29	12.93	9.07	12.49	10.48	11.27	6.99	4.95	5.74	8.85	6.12	22.97	17.24	10.15	8.09	11.73	8.18	13.66	9.22	12.38	9.07	11.15	6.65
KUZ M9588	4.10	6.70	4.84	7.57	12.88	14.91	22.57	19.68	11.68	9.54	13.53	10.30	12.80	10.38	12.33	9.09	11.66	7.10	5.31	5.90	9.51	7.02	24.94	16.79	10.22	7.79	12.00	8.77	14.43	9.72	13.42	8.99	10.85	7.40
KUZ M9590	3.78	6.17	4.78	7.58	12.07	13.84	20.90	19.39	11.25	8.93	13.08	10.13	13.16	10.44	12.06	8.56	10.31	7.09	5.17	5.65	9.38	6.38	23.07	16.52	10.18	7.78	11.87	8.56	13.78	9.54	12.28	8.71	8.84	6.58
KUZ M9594	3.57	5.49	4.22	6.42	9.91	12.48	18.83	16.06	10.33	8.11	12.30	8.42	12.65	9.73	11.63	7.91	11.13	6.78	4.68	5.86	8.57	5.85	20.94	13.53	9.37	6.75	11.67	7.60	13.22	8.56	11.77	7.44	9.13	5.98
KUZ M9985	3.80	6.09	4.74	7.88	13.14	14.64	25.87	20.10	11.64	9.07	14.37	10.97	14.16	10.95	13.40	9.24	11.73	7.30	5.38	6.03	8.61	6.65	25.89	17.45	10.32	8.31	12.77	9.10	14.77	10.51	13.94	9.74	11.55	7.56
KUZ M9987	3.88	6.09	4.82	7.44	11.88	14.08	23.26	19.65	10.63	9.18	13.90	10.43	14.25	10.65	13.89	8.80	11.42	7.07	5.25	6.32	8.83	6.74	24.80	16.50	10.52	7.81	12.98	9.15	15.35	10.32	12.10	9.09	10.61	7.02
KUZ M9988	3.43	5.56	3.85	6.34	11.68	13.60	21.28	17.05	10.59	8.17	12.46	9.75	12.26	9.96	12.17	8.54	12.12	7.02	4.22	5.51	8.35	6.25	24.13	15.12	9.16	7.22	11.57	7.46	13.62	9.50	12.12	8.41	10.40	6.87
KUZ M9989	3.80	6.08	5.15	8.54	11.73	14.30	22.50	20.61	11.90	9.68	14.12	10.94	13.11	10.95	12.39	8.51	9.14	6.38	5.25	6.60	9.25	7.13	26.30	18.93	10.04	8.02	12.77	9.00	14.43	10.60	13.68	9.57	9.72	7.36
KUZ M9990	3.90	6.46	4.68	7.88	12.26	14.71	22.23	19.53	10.91	8.86	14.04	10.66	13.36	10.69	13.38	9.15	11.65	6.90	5.15	6.42	9.33	7.31	24.09	16.53	10.43	7.72	12.93	8.38	15.20	9.82	14.33	9.36	10.50	6.54
KUZ M9991	3.80	6.13	4.71	7.21	10.85	13.75	22.25	18.83	10.23	8.42	13.26	9.82	13.13	9.95	12.28	8.32	10.37	6.31	5.05	6.26	9.43	6.83	22.81	16.20	10.20	8.14	12.18	8.38	14.36	9.30	11.79	7.77	9.46	6.38
KUZ M9993	4.00	6.53	4.66	7.53	11.90	14.28	20.56	18.10	11.20	8.44	13.71	10.43	12.52	9.92	13.09	7.79	10.75	6.81	5.26	6.24	8.90	6.21	23.73	14.95	9.72	7.45	12.26	8.33	14.15	9.66	12.72	8.66	10.39	6.84
KUZ M9994	3.70	5.73	4.30	7.03	11.79	13.89	22.60	18.53	10.79	8.40	13.46	9.71	12.78	10.10	13.14	9.00	11.05	6.91	5.00	6.40	8.65	6.79	24.38	16.40	10.81	8.03	12.48	8.47	14.30	9.80	13.31	8.50	11.16	6.99
KUZ M10001	3.90	6.18	4.73	7.86	12.03	15.07	20.48	17.68	10.50	8.31	13.24	9.15	13.08	10.47	12.68	8.83	10.88	6.41	5.72	6.44	9.00	6.27	22.76	16.64	10.50	8.08	12.42	8.19	14.53	8.98	13.34	9.13	10.66	7.43
KUZ M10004	3.86	6.21	5.21	8.35	12.31	15.09	22.74	20.16	11.81	9.49	14.23	10.39	14.13	10.50	13.51	9.13	11.31	6.73	5.14	6.28	9.61	6.74	25.52	16.12	10.85	8.06	12.82	8.77	15.33	9.78	13.85	9.44	11.06	7.28
KUZ M10005	3.85	5.68	4.81	7.15	10.55	12.77	20.99	17.75	10.56	7.92	13.06	9.38	12.67	9.46	12.67	8.21	11.40	6.25	5.04	5.32	8.58	5.82	22.15	15.08	9.52	7.32	11.41	8.08	13.34	8.86	11.86	8.00	9.62	6.25
KUZ M10006	4.09	6.39	4.90	7.94	11.51	13.11	21.50	18.16	11.71	9.48	13.61	10.53	12.69	10.68	12.43	9.33	12.48	7.18	5.19	5.68	8.76	6.35	22.57	16.54	10.60	8.32	12.68	8.28	14.16	9.85	13.66	9.31	10.45	7.09
NSMT KK42	3.74	5.45	4.18	6.52	11.28	14.33	20.32	17.36	10.88	8.79	13.17	10.03	12.28	9.98	12.03	8.62	11.40	6.56	5.07	5.64	8.55	6.44	22.53	14.63	9.92	7.77	12.01	7.99	13.61	9.01	12.90	8.61	10.26	6.63
NSMT KK122	3.65	5.52	4.14	6.46	10.76	13.00	19.76	18.35	10.63	7.91	12.43	9.27	12.49	9.46	12.57	7.80	7.30	6.27	5.00	5.52	8.25	5.99	21.22	15.88	9.29	6.76	11.66	8.19	13.44	8.41	12.44	7.88	9.26	6.10

continued

Table S1. *Continued*

Specimen reference No.	Measurements																																			
	LI^1	WI^1	LI^2	WI^2	LI^3	WI^3	LC^1	WC^1	LP^1	WP^1	LP^2	WP^2	LP^3	WP^3	LP^4	WP^4	LM^1	WM^1	LI_2	WI_2	LI_3	WI_3	LC_1	WC_1	LP_1	WP_1	LP_2	WP_2	LP_3	WP_3	LP_4	WP_4	LM_1	WM_1
**Females**																																				
HUNHM 13313	3.33	4.95	4.16	6.03	8.26	10.94	12.56	10.95	9.84	7.66	11.57	8.61	11.72	8.63	11.38	7.78	9.57	6.03	4.63	5.22	7.34	5.28	13.10	8.46	8.82	6.88	10.45	6.87	12.28	7.75	11.19	7.29	8.85	5.76
HUNHM 13351	3.34	5.07	4.07	6.52	9.15	10.59	13.70	11.64	9.58	7.98	11.34	8.22	11.23	8.63	11.17	7.32	10.23	5.80	4.36	5.01	8.64	5.68	12.85	9.05	8.57	6.24	10.68	6.50	12.59	7.81	11.37	6.82	8.28	5.40
KUZ M9290	3.28	5.34	3.93	6.01	9.86	12.01	14.72	11.88	10.09	8.14	11.96	9.40	11.91	9.10	11.64	7.75	10.90	6.28	4.59	5.34	8.61	5.62	13.82	9.63	9.11	6.57	11.34	7.44	13.30	8.30	11.28	7.45	8.28	5.95
KUZ M9291	3.62	5.53	4.51	6.31	9.02	12.01	13.22	11.10	9.16	8.04	11.83	9.22	11.23	9.24	11.32	8.31	11.01	6.63	5.08	5.27	8.74	6.07	13.08	8.97	8.93	7.16	11.76	7.65	12.90	8.28	12.62	8.03	10.53	6.34
KUZ M9295	3.55	5.44	4.25	6.45	8.70	10.47	13.32	11.52	9.77	7.79	11.63	8.81	11.77	9.09	12.18	8.12	9.64	6.40	4.90	5.21	8.60	5.66	13.27	9.09	9.19	6.69	11.24	7.21	12.81	8.30	11.61	7.69	9.23	6.28
KUZ M9297	3.33	5.16	4.06	6.00	8.22	10.68	12.93	11.03	10.10	7.98	12.01	9.34	11.95	9.53	12.21	8.78	10.10	6.17	4.88	4.95	8.30	5.39	12.33	8.52	6.03	5.76	11.67	7.70	12.92	8.23	12.90	8.30	10.96	6.17
KUZ M9309	3.47	5.42	4.37	6.51	9.92	11.94	14.47	11.51	10.58	8.00	12.20	9.20	12.64	9.35	12.44	8.14	12.05	6.56	4.91	5.06	8.47	6.34	14.69	9.39	9.39	7.40	11.43	7.34	13.21	8.86	12.10	8.00	9.75	6.00
KUZ M9311	3.28	5.20	3.96	5.91	9.47	11.09	13.61	10.90	10.02	7.23	11.58	8.82	11.36	9.00	11.30	7.68	11.42	6.51	4.70	5.11	8.04	5.38	13.04	8.71	8.77	6.26	11.11	7.27	12.40	7.69	11.26	7.55	10.05	6.22
KUZ M9437	3.43	5.17	4.26	5.96	8.34	10.96	13.30	11.15	10.12	7.43	11.92	8.95	11.53	9.26	12.12	7.98	10.20	5.50	4.50	5.14	8.54	5.55	13.30	8.98	8.65	6.88	10.87	7.42	12.85	7.94	12.00	7.60	9.82	6.49
KUZ M9440	3.55	5.10	4.41	6.19	8.88	10.99	13.13	10.27	9.58	7.60	11.73	8.91	11.26	9.09	11.35	8.01	10.27	6.18	4.81	5.07	7.89	5.33	12.87	8.49	9.12	6.42	10.68	7.18	12.72	8.05	12.26	7.73	10.54	6.36
KUZ M9441	3.29	4.94	4.46	6.74	8.90	11.26	12.90	11.60	9.85	7.99	11.83	9.30	11.50	9.28	11.41	8.16	11.10	6.94	4.68	5.21	8.19	5.46	12.92	9.07	9.31	7.14	10.87	7.33	11.98	8.03	11.24	7.61	9.40	5.85
KUZ M9442	3.11	4.58	3.83	5.63	7.65	9.58	12.87	10.48	9.20	7.50	10.82	8.55	10.61	8.51	10.83	7.55	9.84	6.00	4.21	4.40	7.59	4.95	12.85	8.74	8.74	6.79	10.58	7.06	11.67	7.93	11.10	7.46	9.58	5.83
KUZ M9968	3.05	4.85	3.88	6.08	8.31	10.24	12.69	11.01	9.31	7.59	10.92	8.57	11.21	8.89	11.07	7.43	10.37	6.25	4.59	4.59	7.91	5.26	12.08	8.46	8.73	6.70	10.29	6.76	11.73	7.90	11.07	7.56	9.09	5.86
NSMT KK55	3.46	4.88	3.98	5.78	8.60	10.34	14.22	11.20	9.68	7.40	11.34	8.43	11.17	8.58	11.27	7.42	9.54	5.70	4.81	4.73	7.91	5.25	12.85	9.03	9.18	6.74	11.03	7.00	12.03	7.86	11.09	7.31	7.81	5.48
NSMT KK62	3.33	5.08	4.24	6.07	8.20	10.29	13.38	11.17	10.50	7.82	11.68	8.71	11.17	9.04	11.57	7.89	10.70	6.37	5.03	4.71	8.56	5.18	13.24	9.15	9.42	6.57	11.36	7.06	12.91	8.12	12.08	7.91	10.23	6.14
NSMT KK67	3.42	4.87	4.02	6.07	7.85	9.85	12.75	10.58	9.47	7.66	11.17	8.77	11.04	9.09	10.15	7.52	9.97	5.59	4.81	5.30	8.22	5.32	12.76	8.78	9.24	6.57	10.09	6.45	12.08	7.99	10.79	7.85	9.78	6.30
NSMT KK125	3.44	5.14	4.31	6.13	7.99	10.54	14.36	11.97	9.65	8.01	11.69	9.42	11.23	9.08	11.25	8.23	10.65	6.35	4.70	4.98	8.74	5.33	13.31	9.14	8.41	6.83	10.93	7.52	12.03	8.38	11.73	7.82	9.62	6.55
NSMT KK131	3.24	4.74	3.82	5.92	7.35	9.52	10.94	10.09	9.43	7.30	10.66	8.17	10.20	7.74	10.42	6.64	9.63	5.39	4.53	5.07	7.54	4.80	12.15	8.30	8.41	5.85	10.53	6.81	11.66	7.46	10.65	6.50	9.23	5.43
NSMT KK135	3.14	4.94	3.59	5.59	7.74	10.21	13.48	11.40	9.96	8.41	11.62	9.26	11.53	9.28	11.21	8.00	10.41	6.45	4.77	5.05	8.27	5.37	13.45	9.20	9.02	6.77	11.61	7.90	13.13	8.46	12.32	7.59	9.58	5.92
NSMT KK139	3.10	4.54	4.17	6.02	7.95	10.33	13.44	10.40	9.17	7.34	10.73	9.13	10.79	9.14	10.49	7.49	9.75	5.78	4.46	4.58	7.48	5.22	12.32	8.54	8.95	6.79	10.95	7.62	12.32	8.05	10.92	7.83	9.75	5.59
NSMT KK146	3.18	4.72	4.00	5.85	7.78	9.86	12.53	10.12	9.59	7.63	10.99	8.21	10.78	8.50	10.85	7.98	10.10	6.05	3.99	4.69	7.42	5.25	11.71	8.18	8.72	6.64	10.43	6.86	11.74	7.53	11.24	7.75	9.34	6.25
NSMT KK154	3.28	5.24	4.20	6.06	7.76	9.57	12.50	10.24	9.83	7.90	12.62	9.47	10.62	8.52	10.60	7.11	10.31	6.63	4.78	5.07	7.17	5.10	12.63	9.16	9.18	6.78	10.28	6.94	11.98	7.91	9.14	6.25	8.15	5.57
NSMT KK156	3.43	5.42	3.93	6.11	8.51	10.16	12.94	10.98	9.82	7.56	10.97	9.17	10.78	9.16	11.15	8.30	9.87	6.13	4.58	4.76	8.01	5.86	13.66	8.69	9.64	6.81	10.38	7.44	12.45	8.06	10.95	7.65	8.99	6.29
NSMT KK158	3.52	5.28	4.45	6.15	8.25	10.30	12.74	10.81	9.46	7.78	11.61	9.09	11.60	9.12	11.65	8.20	10.19	5.72	4.54	5.46	7.89	5.73	12.00	9.26	9.35	6.75	10.76	7.40	12.54	8.23	11.09	7.45	9.39	5.95
NSMT M17123	3.41	5.00	4.71	6.28	9.41	12.18	14.64	12.01	10.37	8.42	12.11	9.65	11.98	9.77	11.16	8.39	10.77	6.70	4.90	5.03	8.62	5.62	13.87	9.13	9.21	7.39	11.51	8.00	12.90	8.88	12.10	8.37	10.11	6.66
NSMT M24719	3.23	4.72	3.80	5.55	7.58	9.81	12.80	10.30	8.47	7.26	11.27	8.36	10.67	8.54	11.13	7.51	10.36	6.16	4.60	4.89	8.21	4.74	12.64	8.54	8.87	6.11	10.88	7.17	11.73	7.61	11.16	7.15	9.49	5.91
NSMT M24723	3.34	4.78	4.70	6.05	7.68	10.00	13.58	10.66	10.40	7.65	11.50	8.66	11.11	8.97	11.16	7.86	9.80	6.00	4.18	5.23	8.15	5.47	13.46	8.70	8.51	6.51	10.93	6.89	12.31	7.50	11.08	7.36	9.43	6.11
NSMT M24724	3.12	4.69	3.60	5.61	7.67	9.87	12.47	10.26	8.68	7.23	11.18	8.47	10.83	8.56	10.81	7.90	11.23	5.73	4.26	5.09	7.66	5.33	11.92	8.40	8.50	6.57	10.29	6.61	11.14	7.83	11.17	7.56	9.69	6.12
NSMT N113	3.30	4.77	4.22	5.96	8.31	10.37	13.55	11.03	9.68	7.28	11.83	8.66	11.94	8.49	11.30	7.24	10.65	5.58	4.60	4.47	8.12	5.34	13.95	8.69	8.86	6.11	11.03	6.84	12.12	7.93	11.69	7.77	9.89	6.25
NSMT PO136	3.36	5.08	4.40	5.57	8.64	10.87	13.70	11.10	9.47	6.58	11.45	8.32	11.42	7.80	10.80	7.00	10.06	5.32	4.22	4.20	7.25	5.42	13.18	8.80	8.95	6.54	10.78	7.15	12.23	7.91	11.16	7.13	9.74	5.56

Table S2. Measurement values for *Callorhinus ursinus*. All values are in millimeters. Measurement abbreviations: L, length of the tooth crown (maximum linear mesiodistal distance across the tooth crown); W, width of the tooth crown (maximum linear vestibulolingual distance across the tooth crown perpendicular to the length); I, incisor; C, canine; P, premolar; M, molar; superscript and subscript numbers indicate positions of upper and lower teeth, respectively. Institutional abbreviations: HUNHM, Botanic Garden, Hokkaido University, Sapporo, Japan; KUZ, Kyoto University Museum, Kyoto University, Kyoto, Japan; NSMT, National Museum of Nature and Science, Tokyo, Japan.

Specimen reference No.	Measurements																																			
	LI^1	WI^1	LI^2	WI^2	LI^3	WI^3	LC^1	WC^1	LP^1	WP^1	LP^2	WP^2	LP^3	WP^3	LP^4	WP^4	LM^1	WM^1	LM^2	WM^2	LI_2	WI_2	LI_3	WI_3	LC_1	WC_1	LP_1	WP_1	LP_2	WP_2	LP_3	WP_3	LP_4	WP_4	LM_1	WM_1
**Males**																																				
HUNHM 9893	2.29	4.32	2.58	4.50	4.85	6.86	12.56	9.45	6.19	4.21	6.33	4.20	6.32	4.32	6.20	4.28	6.94	3.32	4.87	3.03	2.63	2.81	5.82	3.14	12.32	7.20	5.33	3.46	6.12	3.81	6.63	4.08	6.85	4.41	7.50	4.06
HUNHM 11560	2.62	4.64	2.86	4.78	5.53	6.98	11.91	10.12	6.72	5.45	6.68	5.23	6.45	5.05	6.75	5.09	6.62	3.80	6.04	3.93	3.10	3.07	6.01	4.16	13.21	8.25	5.64	4.27	6.50	4.71	6.88	4.93	6.95	4.95	7.16	4.03
HUNHM 13349	2.53	4.14	2.84	4.36	4.26	6.55	10.83	7.84	5.89	4.52	6.08	4.73	6.25	4.50	6.01	4.77	6.56	3.41	5.37	3.31	3.20	3.49	5.52	4.23	10.10	5.68	5.28	3.81	6.30	4.25	6.87	4.44	6.79	4.62	7.33	4.17
KUZ M10017	2.43	4.34	2.89	4.65	3.94	6.45	10.65	8.80	6.10	4.36	6.39	4.28	6.53	4.39	6.36	4.35	6.60	3.28	5.48	3.04	3.19	3.17	5.78	3.14	11.49	7.39	5.62	3.96	6.18	4.27	6.56	4.35	7.06	4.71	7.37	4.11
KUZ M10019	2.48	4.42	2.75	4.57	4.26	6.66	12.26	8.71	6.04	4.73	6.35	4.77	6.26	4.82	5.73	4.78	6.88	3.59	2.89	2.42	3.11	3.65	6.05	3.57	12.46	8.28	5.29	3.96	6.51	4.38	6.98	4.73	6.67	4.51	6.98	4.06
KUZ M10023	2.37	4.09	2.56	4.64	4.62	5.83	12.19	9.26	6.37	4.96	6.23	4.35	6.25	4.46	6.63	4.28	6.42	3.37	5.34	3.06	3.10	3.09	5.39	3.55	11.93	7.45	5.65	4.24	6.26	3.94	6.88	4.47	7.09	4.46	7.61	4.10
KUZ M10027	2.71	5.19	3.06	5.40	5.51	7.95	15.51	11.38	7.11	5.37	6.83	5.29	7.07	5.07	6.67	5.00	7.04	3.85	5.93	3.66	3.51	3.84	6.25	4.53	15.54	8.99	5.91	4.43	6.71	4.67	7.39	4.95	7.62	5.43	7.88	4.71
KUZ M10031	2.65	4.84	2.94	5.26	4.92	7.69	12.52	10.01	6.72	5.25	6.80	5.12	6.81	5.01	7.08	4.94	6.77	3.84	6.15	3.86	3.42	3.68	5.54	3.80	12.55	8.43	5.56	4.06	5.97	4.71	7.15	5.00	7.12	5.15	7.43	4.43
KUZ M10033	2.58	5.00	2.82	5.48	4.67	7.54	11.70	9.99	6.40	5.30	6.29	5.08	6.37	4.89	6.83	4.98	6.69	3.68	5.84	3.61	3.45	3.32	5.65	3.96	12.36	8.37	5.57	4.15	5.87	4.55	6.93	4.81	6.92	5.04	7.70	4.71
KUZ M10034	2.39	4.48	2.81	4.95	4.62	6.69	12.44	10.06	6.05	4.71	6.30	4.78	6.17	4.78	6.47	4.67	6.70	3.77	5.76	3.46	3.32	3.28	6.05	3.31	12.17	8.09	5.36	4.14	6.18	4.45	6.89	4.80	6.88	4.94	7.30	4.10
KUZ M10043	2.49	4.36	2.71	4.66	4.30	6.35	11.57	8.50	5.99	4.69	5.95	4.44	5.88	4.70	6.12	4.56	6.55	3.33	4.89	3.39	3.43	3.20	5.52	3.66	11.17	6.19	5.40	3.87	6.02	4.22	6.57	4.63	6.30	4.97	6.86	3.82
KUZ M10051	2.25	4.12	2.49	4.56	4.04	6.87	10.88	7.92	5.97	4.05	5.92	4.06	5.69	4.04	5.99	4.23	6.17	3.18	5.71	3.60	3.17	2.81	5.50	3.47	12.07	6.38	5.30	3.76	6.07	3.75	6.47	4.08	6.37	4.48	6.77	3.63
KUZ M10067	2.49	4.73	2.78	5.05	4.65	6.76	12.35	9.19	6.17	4.61	6.23	4.46	6.22	4.30	6.14	4.37	6.96	3.65	6.27	3.68	3.19	3.17	5.82	3.50	12.04	7.60	5.10	3.82	6.14	4.34	6.69	4.52	6.66	4.74	6.97	4.11
KUZ M10068	2.43	4.81	2.73	5.01	4.73	6.88	13.04	9.73	6.61	5.21	6.57	4.81	6.60	4.59	6.99	4.40	6.90	3.35	6.13	3.06	3.29	3.42	6.34	3.74	13.23	8.35	5.86	4.43	6.47	4.53	7.45	4.82	7.32	4.56	7.31	4.02
KUZ M10069	2.31	3.99	2.59	4.45	4.47	6.10	10.63	7.57	5.57	4.27	5.87	4.40	5.84	4.23	5.82	4.10	6.49	3.33	4.30	2.82	3.03	3.33	5.28	3.69	11.09	6.19	5.20	3.74	5.98	4.12	6.35	4.63	6.24	4.61	6.91	4.22
KUZ M10092	2.56	4.34	2.89	4.79	5.28	6.83	11.88	9.78	6.33	5.08	6.44	4.78	6.70	4.85	6.40	4.59	7.56	3.74	4.97	3.06	3.21	3.25	6.93	3.38	11.93	7.16	5.63	4.05	6.06	4.70	7.11	4.75	7.17	5.19	6.96	4.33
KUZ M10093	2.50	4.84	2.78	5.10	4.48	6.39	10.78	8.77	6.20	4.84	6.33	4.67	6.37	4.56	6.71	4.76	6.71	3.54	5.32	3.28	3.33	3.33	5.45	3.59	11.10	7.11	5.26	4.03	6.09	4.58	6.60	4.86	6.89	4.87	7.33	4.19
KUZ M10094	2.38	4.39	2.69	4.70	4.33	6.16	10.77	7.84	5.85	4.49	5.94	3.95	6.22	4.24	6.41	3.93	6.04	2.88	5.78	3.06	3.38	2.99	5.51	2.78	11.19	6.83	5.52	3.64	6.12	3.55	6.57	4.03	6.90	4.23	6.95	3.49
KUZ M10095	2.38	3.72	2.50	4.44	4.83	7.09	11.91	8.50	5.46	4.49	5.62	4.75	5.82	4.62	6.02	4.44	6.75	3.29	6.45	3.54	2.97	2.89	4.92	3.29	11.77	6.70	5.38	3.53	6.19	4.08	6.74	4.28	6.63	4.51	7.27	4.00
KUZ M10098	2.48	4.07	2.72	4.35	4.54	6.80	13.48	10.36	6.13	4.74	6.23	4.66	6.32	4.84	6.38	4.61	6.61	3.39	5.30	3.27	3.18	3.06	4.95	3.81	13.67	8.28	5.54	3.93	6.00	4.27	6.64	4.52	6.80	4.54	6.97	4.20
KUZ M10099	2.30	4.09	2.47	4.22	3.97	6.37	11.61	8.40	5.66	4.83	5.85	4.69	5.80	4.43	5.25	4.16	6.97	3.30	4.56	3.07	3.14	3.10	4.71	3.23	11.83	6.59	5.44	3.72	5.85	4.47	6.29	4.43	6.37	4.36	7.06	3.90
KUZ M10101	2.54	4.00	2.63	4.22	4.28	6.08	11.73	8.86	5.88	4.19	6.23	4.20	6.42	4.19	6.40	4.38	5.70	3.26	5.60	3.09	3.05	3.19	5.41	2.68	12.75	7.46	5.36	3.61	5.77	4.02	6.67	4.47	6.67	4.38	6.20	3.60
KUZ M10102	2.35	4.22	2.83	4.64	4.44	6.09	11.78	8.71	6.26	4.95	6.38	4.91	6.50	5.06	6.71	4.71	7.00	3.88	5.25	3.31	2.97	2.98	5.96	3.18	12.83	7.40	6.17	4.08	6.79	4.67	7.46	5.30	7.54	5.28	7.92	4.48
KUZ M10103	2.67	4.57	2.93	4.70	4.44	6.25	11.83	9.30	5.94	4.64	5.88	4.31	5.70	4.26	6.09	4.25	6.88	3.58	4.89	2.86	3.49	3.14	5.28	3.08	12.15	8.68	5.29	3.68	5.73	4.13	6.22	4.24	6.25	4.36	6.42	4.07
KUZ M10106	2.25	4.10	2.49	4.51	4.10	6.67	11.93	9.15	5.88	4.66	5.68	4.78	5.35	4.55	5.96	4.59	6.72	3.68	4.67	3.08	3.10	3.54	6.18	3.84	11.64	7.36	4.86	3.83	5.98	4.35	6.27	4.47	6.28	4.38	6.80	4.47
KUZ M10107	2.54	4.60	2.69	4.94	4.86	7.00	13.07	10.23	6.82	5.07	6.43	4.80	6.42	4.72	6.66	4.81	6.58	3.56	5.64	3.61	3.50	2.85	5.93	3.24	12.66	7.64	5.86	3.76	6.58	4.25	7.33	4.70	7.19	4.64	7.38	4.21
KUZ M10108	2.33	4.48	2.81	4.65	4.86	7.70	13.53	10.02	6.40	5.24	6.82	5.43	6.73	5.41	6.94	5.06	7.02	4.18	5.44	3.43	3.13	3.32	6.24	3.37	14.26	8.53	6.10	4.26	6.86	4.61	7.42	4.80	7.10	5.02	8.11	4.81
KUZ M10109	2.29	4.15	2.63	4.56	4.95	6.71	11.50	8.89	5.72	4.11	5.85	4.16	5.87	4.12	6.10	4.40	6.34	3.58	5.81	3.40	3.09	3.29	5.61	3.43	11.37	7.13	5.03	3.59	5.91	4.17	6.57	4.45	6.35	4.42	6.79	4.16
KUZ M10110	2.44	4.48	2.72	4.80	4.52	7.40	12.00	9.22	6.55	4.89	6.31	4.52	6.26	4.51	6.02	4.46	6.73	3.75	2.91	2.58	3.00	3.23	6.88	3.86	13.00	7.32	5.58	3.94	6.41	4.26	6.98	4.72	6.86	4.68	7.20	4.08
KUZ M10113	2.45	4.34	2.73	4.76	5.62	6.98	12.54	9.54	6.14	4.78	6.41	4.70	6.36	4.59	6.52	4.45	6.66	3.38	5.08	3.25	3.15	2.85	5.74	3.67	11.66	7.71	5.56	3.58	6.21	4.26	6.67	4.61	7.32	4.60	6.89	4.19
KUZ M10116	2.34	4.29	2.54	4.37	4.18	6.17	12.56	8.04	5.81	4.77	6.12	4.71	5.78	4.45	6.28	4.57	6.31	3.49	4.57	2.90	2.96	3.10	5.64	3.23	12.15	6.72	5.35	4.08	6.34	4.20	6.68	4.38	7.03	4.67	7.10	4.35
KUZ M10120	2.63	4.85	2.83	4.99	4.97	7.72	12.36	9.05	6.36	5.22	6.29	4.78	6.25	4.93	6.57	4.80	6.78	3.80	3.89	3.47	3.25	3.68	6.11	3.52	12.76	7.06	6.12	4.20	6.58	4.42	6.64	4.85	6.89	4.64	7.30	4.17
KUZ M10121	2.37	4.25	2.62	4.76	4.71	6.89	11.00	9.13	6.13	4.60	6.14	5.05	6.26	4.87	6.27	4.59	6.41	3.58	4.81	3.24	3.34	3.41	6.05	3.17	11.72	7.26	5.64	3.76	6.05	4.40	6.80	4.82	6.93	4.56	7.08	4.05
KUZ M10124	2.37	4.67	2.62	4.89	5.35	7.31	13.20	9.74	6.56	5.11	6.78	5.05	6.70	4.97	6.89	5.01	7.40	3.82	5.95	3.50	3.16	3.21	6.58	3.24	12.82	7.87	5.75	4.15	6.63	4.99	7.02	4.79	7.23	4.89	7.44	3.93
KUZ M10126	2.21	4.37	2.65	4.90	5.15	7.21	12.60	9.48	6.77	5.22	6.81	5.14	7.04	4.83	7.14	4.77	7.46	3.82	6.07	3.54	3.06	3.15	6.23	3.40	12.62	7.19	5.66	4.15	6.45	4.52	7.32	4.64	7.46	4.71	8.33	4.15
KUZ M10141	2.44	4.20	2.80	4.45	4.76	7.08	13.00	9.53	5.68	4.84	6.19	4.81	5.94	4.95	6.07	4.58	6.10	3.51	5.41	3.65	3.09	3.22	6.15	3.50	13.04	7.62	5.39	3.93	5.85	4.21	6.25	4.44	6.31	4.71	6.99	3.73
KUZ M10279	2.18	3.64	2.33	4.04	3.86	6.56	11.72	8.70	6.15	4.77	6.27	4.57	6.37	4.59	6.71	4.46	6.91	3.56	6.01	3.56	2.83	2.89	5.28	3.31	12.05	7.08	5.26	3.86	6.04	4.20	6.26	4.46	6.74	4.65	7.33	3.61
KUZ M10281	2.48	4.57	2.58	4.77	5.14	6.91	12.05	10.07	6.35	4.70	6.39	4.65	6.48	4.80	6.50	4.65	7.12	3.86	5.79	3.36	3.28	3.30	5.55	3.37	12.81	8.18	5.17	4.06	6.03	4.19	6.68	4.52	6.97	4.60	7.48	4.17
KUZ M10372	2.26	4.37	2.55	4.62	4.26	5.51	12.39	9.47	5.90	4.63	5.79	4.64	5.90	4.71	6.03	4.72	6.82	3.51	4.90	3.18	3.31	2.92	5.60	3.14	11.87	7.90	5.29	3.98	5.97	4.21	6.32	4.73	6.33	5.09	7.52	4.13
NSMT KK70	2.09	3.95	2.40	4.12	4.45	6.18	12.46	9.72	5.92	4.38	6.07	4.49	6.19	4.56	6.23	4.66	6.38	3.58	5.20	3.67	2.99	2.91	5.02	2.90	12.19	7.28	5.47	3.59	6.06	4.23	6.59	4.44	6.66	4.58	7.37	4.29
NSMT KK162	2.34	4.88	2.64	4.97	4.12	6.40	11.15	9.15	6.30	4.93	6.11	4.32	6.11	4.37	6.75	4.16	6.59	3.37	5.36	3.15	3.07	3.27	5.55	3.13	11.26	7.38	6.02	3.76	5.97	3.92	6.67	4.16	7.06	4.52	7.72	4.04
NSMT M1994	2.14	3.74	2.43	3.94	4.01	5.87	10.15	7.78	5.35	4.12	5.38	4.05	5.54	4.06	5.77	4.14	6.21	3.39	5.99	3.27	2.90	2.78	4.86	3.34	11.24	6.89	4.49	3.37	5.42	3.93	5.87	4.07	6.03	4.20	6.48	3.73
NSMT M1996	2.62	4.47	2.94	5.01	4.40	7.03	12.53	8.61	6.33	4.63	6.44	5.08	6.09	4.54	6.86	4.65	7.25	3.62	5.85	3.23	3.34	3.01	5.76	3.56	11.78	6.78	6.35	4.35	6.25	4.44	7.08	4.67	6.98	5.05	7.97	4.39

continued

Table S2. *Continued*

Specimen reference No.	Measurements																																			
	LI^1	WI^1	LI^2	WI^2	LI^3	WI^3	LC^1	WC^1	LP^1	WP^1	LP^2	WP^2	LP^3	WP^3	LP^4	WP^4	LM^1	WM^1	LM^2	WM^2	LI_2	WI_2	LI_3	WI_3	LC_1	WC_1	LP_1	WP_1	LP_2	WP_2	LP_3	WP_3	LP_4	WP_4	LM_1	WM_1
**Females**																																				
KUZ M10018	2.36	3.49	2.69	3.77	3.46	4.90	7.90	5.71	5.91	4.48	5.69	4.43	5.67	4.48	6.03	4.39	5.43	2.99	4.83	2.88	2.83	2.66	4.49	2.98	7.13	4.77	5.14	3.60	5.67	3.88	6.02	4.12	6.16	4.04	6.60	3.48
KUZ M10020	2.10	3.48	2.34	3.72	3.06	4.74	7.62	5.49	5.25	4.20	5.47	4.02	5.32	4.13	5.55	3.99	6.46	3.49	4.96	2.98	2.70	2.77	4.84	2.79	6.63	4.62	5.09	3.78	5.23	3.96	5.79	3.99	5.87	4.22	6.72	3.93
KUZ M10021	2.35	3.85	2.70	3.90	3.34	4.44	7.03	5.85	5.77	4.23	5.52	4.08	5.46	3.99	5.57	3.97	6.15	3.16	5.10	3.08	3.09	2.81	4.66	2.79	6.44	4.40	5.28	3.24	5.56	3.56	6.17	3.73	6.22	3.86	6.60	3.62
KUZ M10024	2.24	3.93	2.41	4.19	3.45	5.53	8.13	5.54	5.26	4.38	5.68	4.46	5.89	4.49	6.28	4.50	6.43	3.27	5.43	3.38	2.69	2.99	5.05	3.15	6.65	4.59	5.08	3.70	5.36	4.04	6.30	4.34	6.02	4.00	6.40	3.83
KUZ M10029	2.28	3.73	2.49	4.19	3.09	5.03	7.77	5.98	5.70	4.49	5.64	4.23	5.81	4.22	5.99	4.47	5.85	3.48	5.03	3.28	3.27	2.70	5.38	2.69	6.82	5.19	5.19	3.84	5.36	4.17	5.78	4.17	6.21	4.36	6.75	4.14
KUZ M10035	2.20	3.87	2.55	4.21	3.42	5.64	9.09	6.56	5.87	4.68	5.87	4.61	5.85	4.49	6.07	4.55	6.88	3.84	5.39	3.54	3.16	2.71	5.14	2.42	7.81	5.32	5.21	3.89	5.82	4.17	6.28	4.11	6.77	4.34	7.23	4.21
KUZ M10038	2.23	3.91	2.52	4.43	3.55	5.18	7.85	5.90	5.53	4.12	5.74	4.16	5.74	4.11	6.02	3.68	6.84	3.29	2.77	2.67	3.81	3.01	5.13	2.80	7.32	5.05	5.41	3.64	5.52	3.84	6.01	4.02	6.04	4.15	6.34	3.85
KUZ M10040	2.14	3.70	2.29	3.91	2.92	4.94	7.48	5.65	5.23	4.15	5.42	3.95	5.53	3.87	5.66	3.71	5.70	3.13	4.40	2.65	2.88	2.62	5.06	2.83	6.25	4.62	4.72	3.43	5.40	3.67	5.87	3.91	6.11	4.20	6.32	3.82
KUZ M10041	2.30	3.96	2.70	3.98	3.76	5.56	8.89	6.65	5.78	4.96	5.99	4.64	6.04	4.67	6.28	4.50	6.27	3.46	4.78	2.97	3.12	3.15	5.04	3.17	7.35	5.52	5.14	3.94	5.77	4.26	6.51	4.69	6.54	4.67	7.25	4.23
KUZ M10044	2.18	3.66	2.64	4.00	3.54	5.31	7.57	5.51	4.86	3.90	5.55	4.03	5.43	4.07	5.84	4.15	6.16	3.36	4.92	3.06	3.01	2.68	4.60	3.00	7.06	4.44	4.84	3.51	5.44	3.67	5.82	3.99	6.20	4.14	6.68	3.78
KUZ M10045	2.27	3.87	2.70	4.09	3.96	5.16	8.49	6.10	5.56	4.18	5.50	4.24	5.50	4.08	5.84	4.10	6.68	3.46	5.33	3.06	3.20	2.85	4.60	2.94	7.76	5.00	5.10	3.57	5.65	3.91	5.98	3.91	6.03	4.21	6.92	3.57
KUZ M10046	2.26	3.74	2.54	3.92	3.67	5.12	7.78	5.58	5.50	4.44	5.54	4.13	5.66	4.30	5.83	4.36	6.35	3.46	3.53	2.90	3.40	2.94	4.99	3.02	6.78	4.36	5.12	3.56	5.66	3.81	6.17	4.24	5.92	4.14	7.01	4.03
KUZ M10048	1.98	3.50	2.18	3.90	3.52	4.95	7.55	6.10	5.28	4.13	5.24	4.08	5.18	4.00	5.08	3.89	5.64	3.19	4.33	2.74	2.64	2.67	4.77	2.68	7.00	4.53	4.84	3.57	5.38	3.73	6.06	4.03	5.92	4.00	5.89	3.85
KUZ M10049	2.21	4.09	2.61	4.52	4.04	5.38	8.84	6.47	5.96	4.76	6.37	4.72	6.25	4.61	6.40	4.42	7.02	3.69	5.45	3.36	3.00	2.95	4.99	2.97	7.95	5.67	5.37	4.07	5.85	3.91	6.63	4.25	6.73	4.45	7.13	4.07
KUZ M10050	2.01	3.65	2.49	3.98	3.78	4.29	7.04	5.39	5.52	3.71	5.48	3.70	5.45	3.67	5.47	3.71	6.47	3.06	4.84	2.91	3.16	2.37	4.96	2.40	6.25	4.13	5.07	2.90	5.28	3.33	6.08	3.58	6.08	3.70	6.51	3.54
KUZ M10053	2.13	3.88	2.63	4.05	3.74	5.11	8.39	5.99	5.55	4.13	5.66	4.06	5.52	4.20	5.78	4.06	5.60	3.13	4.90	2.66	3.00	2.79	4.65	2.78	7.84	4.81	5.03	3.49	5.82	3.58	6.13	4.01	6.33	3.99	6.86	3.61
KUZ M10055	2.50	4.36	2.78	4.59	3.66	5.55	7.84	6.07	5.96	4.35	5.93	4.38	5.76	4.17	6.08	4.49	6.72	3.36	5.46	3.05	4.20	2.96	4.84	3.02	6.77	5.02	5.49	3.87	5.79	3.83	6.41	4.12	6.45	4.08	6.98	3.81
KUZ M10056	2.33	3.70	2.74	4.09	3.67	4.76	8.02	5.83	5.42	4.52	5.06	4.11	5.21	4.03	5.52	4.20	5.44	2.97	4.69	2.84	3.04	2.90	5.00	2.73	7.32	4.77	5.16	3.47	5.37	3.73	5.81	3.85	5.98	4.04	6.26	3.66
KUZ M10058	2.44	4.06	2.67	4.34	3.78	5.45	7.24	4.86	5.67	4.09	5.55	3.86	5.59	4.07	6.02	4.01	6.69	3.49	4.98	3.44	2.96	2.53	4.81	2.63	6.48	4.17	4.82	3.47	5.51	3.63	5.85	4.08	6.20	4.40	6.72	3.98
KUZ M10059	2.11	3.74	2.33	3.98	3.54	4.81	7.42	5.55	5.40	4.08	5.20	4.01	5.13	3.90	5.57	3.87	5.78	3.18	3.98	2.28	2.94	2.78	5.13	2.90	6.40	4.51	5.05	3.35	5.21	3.45	5.72	3.75	5.84	3.84	6.10	3.59
KUZ M10060	2.25	3.78	2.45	3.96	3.48	5.79	7.91	6.29	5.54	4.18	5.41	4.10	5.16	4.33	5.50	4.16	6.47	3.63	5.00	3.10	3.09	2.83	5.06	2.66	6.71	4.57	4.90	3.65	5.54	3.70	5.91	4.07	5.74	3.85	6.62	3.78
KUZ M10061	2.23	3.93	2.54	4.44	3.38	5.39	7.44	5.69	5.55	4.46	5.56	4.35	5.42	4.53	5.41	4.34	5.92	3.34	3.46	2.30	2.96	3.10	5.23	3.15	6.56	4.59	4.81	3.50	5.40	3.96	6.13	4.35	6.19	4.44	6.38	4.38
KUZ M10062	2.16	3.92	2.42	4.30	3.60	5.56	9.08	6.78	5.42	4.46	5.60	4.26	5.62	4.18	5.66	4.27	6.66	3.46	5.22	3.03	3.03	2.42	4.36	2.83	6.94	5.39	4.89	3.82	5.13	4.12	5.48	4.29	5.89	4.34	6.36	3.92
KUZ M10064	2.12	3.95	2.51	4.51	3.77	5.28	8.07	6.13	5.75	4.35	6.00	4.32	6.10	4.44	5.90	4.30	5.97	3.36	4.81	3.51	3.05	3.29	4.95	3.17	7.09	4.62	5.44	3.63	6.09	3.70	6.55	3.85	6.36	4.24	6.78	3.81
KUZ M10065	2.21	4.24	2.67	4.68	3.85	5.93	9.15	6.73	5.64	4.44	5.98	4.37	5.81	4.61	6.18	4.64	6.92	3.65	5.32	3.14	3.10	2.64	5.34	3.04	7.35	5.20	4.80	3.89	5.67	3.98	6.21	4.16	6.48	4.57	6.89	4.09
KUZ M10081	2.16	3.73	2.52	4.23	3.47	5.06	7.33	5.48	5.23	4.10	5.46	4.02	5.44	4.32	5.51	4.07	5.58	3.52	3.01	2.66	2.73	2.79	5.42	2.77	6.82	4.74	4.79	3.55	5.39	3.67	5.71	4.02	5.95	4.13	6.09	3.71
KUZ M10082	2.29	3.53	2.46	4.04	3.79	6.08	8.55	6.28	5.44	4.31	5.61	4.34	5.66	4.17	5.56	4.12	6.44	3.57	2.73	2.51	2.93	2.88	4.95	2.72	7.68	4.94	5.20	3.84	5.96	4.14	6.23	4.36	6.27	4.46	7.15	3.96
KUZ M10085	2.06	3.75	2.44	4.12	3.69	4.68	8.28	5.48	5.26	3.93	5.41	4.05	5.27	4.08	5.46	4.08	6.28	3.16	4.48	2.64	2.79	2.64	4.50	2.91	6.94	4.54	4.68	3.45	5.32	3.79	5.69	3.89	5.92	3.95	6.41	3.45
KUZ M10087	2.08	3.25	2.48	3.75	3.31	4.32	6.93	5.48	5.16	3.99	5.31	3.97	5.29	3.93	5.82	4.00	5.82	2.82	5.85	2.89	2.90	2.28	4.54	2.71	6.08	4.29	5.08	3.74	5.11	3.79	5.72	3.93	5.85	4.15	6.53	3.52
KUZ M10089	2.43	3.89	2.50	4.06	4.30	5.76	8.30	6.05	6.19	4.91	6.08	4.55	6.01	4.80	6.14	4.81	6.55	3.84	5.29	3.32	3.17	2.69	5.00	3.09	7.41	4.90	5.53	4.33	6.00	4.25	6.63	4.53	6.22	4.60	6.78	4.14
KUZ M10090	2.39	3.98	2.76	4.22	3.94	5.93	9.29	6.89	6.02	4.76	6.47	4.75	6.29	4.55	6.73	4.52	7.12	4.01	5.38	3.12	2.84	2.85	5.57	2.94	7.86	5.61	5.57	3.89	5.97	4.32	6.69	4.35	6.77	4.41	7.09	3.93
KUZ M10114	2.08	3.74	2.27	4.17	3.36	5.42	8.10	5.74	5.69	4.40	5.56	4.04	5.28	4.23	5.44	4.21	6.25	3.55	5.07	3.08	2.61	2.63	4.82	3.09	7.44	5.11	4.98	3.74	5.78	3.67	6.16	3.96	6.45	4.12	6.63	3.82
KUZ M10115	2.18	3.68	2.46	4.11	3.66	5.28	8.83	6.19	5.45	4.38	5.74	4.31	5.76	4.38	6.12	4.37	6.36	3.34	5.38	3.26	3.07	2.77	4.55	3.02	8.56	5.24	5.08	3.63	5.61	3.85	6.04	4.35	6.38	4.48	7.20	4.21
KUZ M10119	1.97	3.52	2.35	3.95	3.27	5.23	7.09	5.15	5.59	4.53	5.55	4.41	5.49	4.42	5.52	4.49	5.83	3.60	4.14	3.29	2.41	2.23	4.35	2.71	6.39	4.51	5.08	3.51	5.67	3.87	5.96	3.96	5.94	4.17	6.18	3.80
KUZ M10123	2.15	3.40	2.33	3.84	3.80	5.00	7.70	5.59	5.59	4.33	5.56	4.32	5.84	4.39	5.52	4.21	6.03	3.38	5.01	2.99	2.93	2.66	4.87	2.67	6.79	4.70	4.90	3.60	5.60	3.91	6.10	4.13	6.28	4.15	6.84	3.85
KUZ M10125	2.26	3.62	2.58	4.24	4.31	5.56	7.80	6.14	5.57	4.21	5.65	4.08	5.79	4.29	5.97	4.31	6.28	3.62	4.48	2.98	3.14	2.84	5.02	2.73	6.64	5.15	5.29	3.72	5.57	3.67	6.24	4.07	6.37	4.30	6.35	3.83
KUZ M10127	2.22	3.86	2.57	4.01	3.67	5.52	7.81	5.90	5.44	4.48	5.58	4.25	5.47	4.29	5.53	4.22	5.87	3.23	4.90	3.15	2.95	2.54	4.88	2.69	7.01	5.01	4.69	3.77	5.33	3.79	5.73	3.89	5.78	4.07	6.89	3.42
KUZ M10128	2.20	3.59	2.48	3.99	3.78	4.28	7.55	5.54	5.09	3.99	5.16	3.98	5.39	4.11	5.56	4.20	5.36	2.93	4.92	3.03	2.80	2.57	4.62	2.47	6.82	4.61	4.86	3.33	5.29	3.20	5.83	3.64	6.03	4.02	6.37	3.65
KUZ M10129	2.20	3.59	2.36	3.77	3.88	4.88	6.87	5.55	5.47	4.11	5.69	3.97	5.68	3.99	5.98	4.09	6.16	3.16	5.32	3.20	2.99	2.76	5.39	2.85	6.49	4.50	4.79	3.86	5.26	3.83	5.86	4.02	6.12	4.56	6.52	3.53
KUZ M10131	2.30	3.95	2.62	4.06	4.09	5.25	7.72	5.98	5.92	4.73	5.88	4.51	6.18	4.46	6.84	4.51	6.82	3.68	4.41	3.29	2.93	3.09	5.28	2.80	7.23	5.12	5.29	4.06	5.77	4.42	6.33	4.40	6.60	4.65	7.00	4.08
KUZ M10132	1.88	3.28	2.30	3.50	3.16	4.42	6.88	4.91	5.19	3.89	5.02	3.69	5.13	3.84	5.23	4.08	6.04	2.93	4.78	2.89	2.72	2.30	4.68	2.37	6.64	4.04	4.87	3.03	5.00	3.38	5.39	3.59	5.32	3.71	6.36	3.53
KUZ M10135	2.26	3.68	2.64	4.17	3.81	5.61	7.57	5.89	5.64	4.24	5.68	4.07	6.22	4.38	6.17	4.22	7.38	3.65	4.14	2.77	2.78	2.52	5.03	2.68	6.34	5.08	5.27	3.63	5.60	3.85	5.98	4.01	6.21	4.43	7.16	3.94
KUZ M10137	2.15	3.61	2.22	3.77	3.00	4.32	7.21	5.31	5.38	4.18	5.16	3.97	5.07	3.78	5.10	3.77	6.03	3.01	4.15	2.62	2.99	2.65	4.75	2.69	6.72	4.95	4.81	3.56	5.18	3.57	5.47	3.74	5.66	3.80	6.06	3.54
KUZ M10138	2.08	3.59	2.33	3.94	3.82	4.94	7.06	5.41	5.18	3.91	5.40	3.93	5.58	3.93	5.91	3.78	6.31	3.12	3.65	2.80	2.85	2.49	5.47	2.45	6.77	4.27	4.83	3.19	5.32	3.35	5.55	3.62	5.52	3.89	6.40	3.55
KUZ M10140	2.09	3.72	2.37	3.98	3.11	4.68	6.99	5.04	5.11	4.11	5.41	3.82	5.56	4.13	5.67	3.90	6.29	2.95	5.24	2.89	2.83	2.71	4.97	2.28	6.49	4.40	4.84	3.52	5.69	3.55	5.97	3.68	5.94	3.73	6.53	3.32
KUZ M10142	2.34	3.40	2.76	3.75	4.08	5.40	7.61	5.75	5.82	4.38	5.54	4.15	5.77	3.91	6.22	4.06	6.46	3.46	4.82	2.75	2.86	2.60	5.05	2.72	6.99	4.59	5.39	3.56	5.79	3.78	6.22	4.06	6.57	4.27	6.57	3.58
KUZ M10144	2.39	3.47	2.64	3.74	3.86	4.61	6.96	4.69	5.23	3.80	5.29	3.68	5.06	3.64	5.43	3.71	5.55	3.21	4.97	2.78	3.19	2.87	5.22	2.25	6.62	4.42	5.02	3.32	5.14	3.08	5.95	3.39	5.46	3.39	6.25	3.40
KUZ M10147	2.33	3.96	2.67	4.50	4.64	5.52	7.53	5.66	5.79	4.66	5.72	4.39	5.74	4.51	6.01	4.40	6.34	3.63	4.73	3.16	3.11	3.09	5.17	2.80	6.80	4.52	5.15	3.68	6.11	3.90	6.30	4.00	6.15	4.12	6.48	3.79

continued

Table S2. *Continued*

Specimen reference No.	Measurements																																			
	LI^1	WI^1	LI^2	WI^2	LI^3	WI^3	LC^1	WC^1	LP^1	WP^1	LP^2	WP^2	LP^3	WP^3	LP^4	WP^4	LM^1	WM^1	LM^2	WM^2	LI_2	WI_2	LI_3	WI_3	LC_1	WC_1	LP_1	WP_1	LP_2	WP_2	LP_3	WP_3	LP_4	WP_4	LM_1	WM_1
**Females**																																				
KUZ M10148	2.18	3.60	2.38	3.89	3.66	5.42	7.93	6.46	5.57	4.77	5.33	4.48	5.69	4.62	5.90	4.66	6.70	3.89	5.44	3.02	2.98	2.58	5.08	2.95	7.06	5.27	5.35	4.16	5.53	4.35	6.14	4.54	6.52	4.55	6.95	4.38
KUZ M10149	2.12	4.12	2.42	4.33	3.29	5.40	7.51	5.93	5.80	4.43	5.54	4.32	5.56	4.13	5.81	4.28	6.71	3.54	4.32	2.53	3.04	2.69	5.03	2.70	6.38	4.67	4.95	3.49	5.44	3.74	6.00	3.96	6.22	4.09	7.06	3.89
KUZ M10151	2.27	3.79	2.70	4.25	3.27	5.39	8.17	5.98	5.70	4.50	5.69	4.09	5.60	4.20	5.76	4.19	6.56	3.70	4.62	3.07	3.11	2.74	5.39	2.58	6.49	4.47	5.32	3.91	5.78	4.06	6.00	4.30	6.08	4.40	6.75	4.25
KUZ M10154	2.52	4.48	2.76	4.79	3.97	6.05	8.78	6.36	5.97	5.06	6.01	4.86	5.95	4.55	6.49	4.35	6.67	3.60	5.66	2.78	3.02	2.89	5.89	3.07	7.73	5.48	5.54	4.30	6.08	4.03	6.48	4.21	6.65	4.29	7.02	3.98
KUZ M10155	2.27	3.66	2.64	4.18	3.75	5.00	7.76	5.73	5.40	4.04	5.33	3.82	5.67	3.89	5.99	3.84	6.00	2.92	4.81	2.77	2.87	2.48	4.70	2.37	7.18	4.60	4.80	3.44	5.13	3.24	5.65	3.51	6.02	3.75	6.45	3.67
KUZ M10157	2.46	3.54	2.70	3.99	3.82	4.91	7.48	5.57	5.42	4.58	5.06	4.02	5.33	4.30	5.26	4.05	5.91	3.31	3.64	2.83	2.90	2.76	4.85	2.70	7.31	4.81	5.18	3.77	5.66	3.71	5.76	4.03	6.12	4.16	6.35	3.69
NSMT KK8	2.25	3.59	2.49	3.88	3.13	5.29	7.39	5.74	5.59	4.18	5.70	4.43	5.65	4.21	6.01	4.44	6.51	3.40	4.68	2.97	2.66	2.41	4.56	2.62	6.67	4.33	4.97	3.35	5.35	3.61	5.89	4.09	6.03	4.11	6.49	3.70
NSMT KK10	2.42	3.81	2.46	3.98	2.86	4.58	7.14	5.29	5.63	4.38	5.55	4.11	5.26	3.87	5.34	4.01	6.10	4.01	4.76	2.86	2.71	2.87	4.46	2.74	6.34	4.42	5.09	3.47	5.56	3.88	5.62	3.72	5.81	4.24	6.34	3.85
NSMT KK22	2.19	3.81	2.55	4.39	3.49	5.70	8.17	5.87	5.31	4.19	5.64	4.09	5.85	4.18	6.08	4.06	6.48	3.52	5.02	2.80	2.95	2.56	4.89	2.37	6.74	4.91	4.76	3.42	5.47	3.82	5.99	3.97	6.20	4.22	7.01	3.81
NSMT KK151	2.46	4.17	2.58	4.42	3.77	5.93	8.91	6.27	5.87	4.62	6.28	4.43	6.54	4.47	6.67	4.57	6.95	3.77	4.49	3.37	2.77	2.53	4.36	2.58	6.21	4.83	4.99	3.47	5.26	3.62	5.84	3.99	5.92	4.09	6.39	3.67
NSMT M1995	2.45	4.46	2.80	4.48	3.52	5.48	7.77	5.89	5.91	4.40	5.94	4.30	5.99	4.52	6.58	4.53	6.59	3.32	5.03	3.11	3.28	2.73	4.90	2.77	7.48	4.85	5.54	3.80	6.34	3.95	6.58	3.96	7.13	4.39	7.68	3.96

Table S3. Measurement values for *Phoca largha*. All values are in millimeters. Measurement abbreviations: L, length of the tooth crown (maximum linear mesiodistal distance across the tooth crown); W, width of the tooth crown (maximum linear vestibulolingual distance across the tooth crown perpendicular to the length); I, incisor; C, canine; P, premolar; M, molar; superscript and subscript numbers indicate positions of upper and lower teeth, respectively. Institutional abbreviations: HUNHM, Botanic Garden, Hokkaido University, Sapporo, Japan; KUZ, Kyoto University Museum, Kyoto University, Kyoto, Japan; NSMT, National Museum of Nature and Science, Tokyo, Japan; TUA, Laboratory of Aquatic Management, Department of Aqua Bioscience and Industry, Faculty of Bioindustry, Tokyo University of Agriculture, Abashiri, Japan.

Specimen reference No.	Measurements																																	
	LI^1	WI^1	LI^2	WI^2	LI^3	WI^3	LC^1	WC^1	LP^1	WP^1	LP^2	WP^2	LP^3	WP^3	LP^4	WP^4	LM^1	WM^1	LI$_2$	WI$_2$	LI$_3$	WI$_3$	LC$_1$	WC$_1$	LP$_1$	WP$_1$	LP$_2$	WP$_2$	LP$_3$	WP$_3$	LP$_4$	WP$_4$	LM$_1$	WM$_1$
**Males**																																		
HUNHM 13325	2.03	2.85	2.45	3.55	3.62	5.13	7.73	6.56	6.23	3.83	8.36	4.28	8.86	4.51	8.48	4.52	7.90	4.48	1.82	2.34	2.27	2.62	7.17	6.17	5.82	3.84	8.13	4.77	9.18	4.91	9.19	4.79	9.70	4.42
HUNHM 13326	2.05	3.25	2.43	3.72	3.72	5.36	9.21	7.40	5.63	4.20	7.26	4.95	8.27	4.81	7.60	4.55	8.27	4.65	1.73	2.20	2.41	2.56	7.77	5.68	5.16	3.66	7.41	4.30	8.17	4.50	8.19	4.40	8.57	4.40
KUZ M9277	1.78	3.11	2.28	3.65	3.89	5.12	7.88	6.52	5.63	3.44	8.04	4.00	8.88	4.49	9.23	4.57	7.05	4.01	1.73	2.03	2.50	2.60	7.23	5.87	5.20	3.63	7.98	4.32	9.23	4.54	8.77	4.62	9.26	4.27
KUZ M9279	2.14	3.32	2.75	4.12	4.01	5.24	7.68	6.88	5.61	4.27	8.58	4.44	8.78	4.79	7.96	4.71	8.05	4.33	2.28	2.54	2.93	2.74	7.29	6.24	5.30	4.18	7.44	4.86	8.77	5.10	8.52	4.85	9.37	4.82
KUZ M9336	2.10	2.83	2.63	3.41	3.95	5.36	8.14	6.92	5.58	4.19	7.70	3.94	8.28	4.30	7.92	4.34	7.60	4.26	1.81	1.97	2.53	2.40	7.71	6.32	4.92	3.61	7.70	4.36	8.71	4.60	7.82	4.34	8.31	4.53
KUZ M9413	2.07	3.10	2.58	3.69	3.77	4.50	7.93	5.07	5.04	3.63	7.64	3.77	7.88	4.10	8.04	4.43	7.34	4.23	1.95	2.05	2.84	2.48	6.95	5.18	4.49	3.79	7.19	4.24	8.45	4.49	8.46	4.48	8.65	4.31
KUZ M9415	2.30	3.39	2.74	3.74	4.00	5.52	8.27	6.88	6.09	4.16	8.72	4.69	9.39	4.92	9.18	4.95	8.94	4.60	1.85	2.18	2.52	2.52	7.81	6.01	5.38	3.99	8.59	4.86	9.33	5.35	8.78	4.92	9.08	4.91
KUZ M9457	2.26	3.24	2.62	3.58	4.03	4.88	8.02	6.92	5.01	3.87	7.76	4.57	8.22	4.89	7.52	4.93	8.26	4.87	2.09	2.33	2.83	2.71	7.58	6.62	5.06	3.63	7.74	5.00	8.75	5.04	8.38	4.83	9.75	4.79
KUZ M9533	1.81	2.52	2.05	2.82	3.49	4.88	8.28	6.31	5.82	3.47	7.85	4.33	7.97	4.49	7.81	4.48	7.30	4.41	1.81	1.95	2.10	2.29	6.93	5.70	4.73	3.46	7.71	4.32	8.43	4.37	8.04	4.41	9.05	4.34
KUZ M9548	1.81	2.91	2.40	3.51	3.60	5.17	8.01	6.60	5.00	3.32	7.78	4.04	8.21	4.28	7.85	4.47	8.02	4.22	1.75	2.00	2.36	2.28	7.16	5.92	4.96	3.54	7.73	4.56	8.45	4.82	7.74	4.61	9.10	4.56
KUZ M9610	2.01	2.45	2.24	3.26	3.99	4.96	7.86	6.61	5.36	4.08	7.67	4.17	7.94	4.45	8.03	4.68	7.57	4.12	1.84	1.93	2.41	2.45	7.02	6.13	5.50	3.79	8.14	4.43	8.57	4.55	8.24	4.27	9.40	4.28
KUZ M9623	2.08	3.46	2.63	3.56	4.16	5.67	8.24	7.07	7.40	4.22	10.29	4.67	10.18	5.12	9.26	4.78	9.34	4.70	2.05	2.26	2.82	2.71	8.04	6.72	5.60	4.08	9.36	4.97	10.26	4.52	9.12	4.26	10.32	4.73
KUZ M9741	1.87	3.04	2.68	3.84	3.89	5.07	8.52	7.31	7.54	4.22	8.67	4.70	8.78	4.73	9.33	4.93	8.49	4.63	1.97	2.18	2.70	2.60	7.53	6.86	5.59	3.91	8.79	4.96	9.39	5.09	8.23	4.92	8.22	4.79
KUZ M9745	2.10	2.73	2.59	3.24	3.48	4.50	7.56	6.41	5.80	4.43	8.14	4.40	8.46	4.52	7.60	4.65	7.68	4.24	1.88	2.19	2.78	2.58	6.99	6.17	5.70	3.93	8.32	4.76	8.85	4.90	8.05	4.72	9.60	4.76
KUZ M9851	2.19	3.53	2.80	4.14	4.01	5.03	8.44	7.00	6.61	4.33	8.68	4.65	9.32	4.86	8.39	5.27	8.24	4.52	2.29	2.68	2.91	2.85	7.14	6.03	5.78	3.83	8.61	5.13	9.71	5.12	9.45	5.07	9.61	4.75
KUZ M9902	2.15	3.29	2.65	3.62	4.05	5.27	7.51	7.10	6.39	3.84	8.08	4.37	8.57	4.57	8.63	4.98	7.88	4.63	1.84	2.23	2.66	2.44	6.43	6.25	5.86	4.23	8.48	4.68	9.21	4.84	8.42	4.65	9.56	4.77
KUZ M10342	2.14	3.05	2.74	3.55	3.77	5.06	8.05	6.79	6.55	4.31	7.86	4.38	8.07	4.77	8.27	5.10	8.30	4.69	2.24	2.34	2.71	2.72	6.92	6.27	5.74	4.24	8.18	4.92	8.81	5.06	8.69	4.68	9.80	4.96
NSMT M24771	2.23	3.52	2.45	3.79	3.79	5.31	8.17	7.19	5.73	4.22	8.46	4.38	8.79	4.33	8.82	4.40	7.71	4.32	1.70	2.22	2.31	2.63	7.22	6.34	5.47	4.01	8.30	4.70	8.99	4.89	8.53	4.39	8.87	4.18
NSMT M29787	2.09	3.33	2.35	3.15	3.70	5.07	8.51	7.10	5.23	3.65	7.96	4.22	8.64	4.55	7.92	4.70	8.55	4.60	2.16	2.37	2.61	2.55	7.80	6.33	5.17	3.81	7.85	4.80	8.80	5.11	8.25	4.63	9.46	4.91
TUA AbG701	2.43	3.19	2.68	3.81	4.25	5.58	8.56	7.93	5.48	3.89	8.66	4.68	9.35	4.76	8.30	5.08	8.18	4.88	2.15	2.17	2.66	2.66	8.35	6.95	5.16	3.80	7.92	5.10	9.21	4.90	9.30	4.96	9.98	5.09
TUA BG902	2.12	2.92	2.47	3.41	4.06	5.20	8.26	6.83	5.40	3.94	7.81	4.41	8.34	4.51	7.87	4.58	7.68	4.58	2.10	2.29	2.56	2.46	7.74	6.00	5.23	4.01	7.95	4.88	8.90	5.16	8.57	4.82	8.45	4.86
TUA BG905	2.18	3.08	2.69	4.00	3.82	5.04	7.78	6.67	6.03	4.13	8.15	4.36	8.64	4.81	8.31	4.96	8.32	4.84	1.94	2.11	2.73	2.64	7.44	6.01	5.80	4.08	8.77	4.91	9.70	5.27	8.89	4.96	9.65	4.81
TUA EG5101	2.07	2.97	2.56	3.94	3.49	4.89	7.39	6.06	5.07	3.77	7.56	3.72	8.32	3.80	7.40	3.94	7.51	4.04	2.06	2.10	2.50	2.70	6.89	5.63	5.37	3.84	7.54	4.31	8.15	4.48	7.16	4.03	8.33	4.21
TUA G18	2.08	3.30	2.55	3.68	3.92	4.90	8.59	6.87	5.93	3.80	8.50	4.25	8.44	4.53	8.21	4.66	7.81	4.76	1.78	2.13	2.35	2.52	7.25	6.16	5.37	4.06	8.28	4.25	8.67	4.45	8.57	4.42	9.25	4.53
TUA G21	2.22	3.19	2.61	3.67	4.08	5.16	9.96	7.52	5.34	3.82	7.58	4.44	8.75	4.99	8.84	4.98	8.28	5.00	2.16	2.26	2.75	2.76	8.06	7.15	5.56	4.13	7.83	5.00	8.57	5.08	8.82	4.88	10.07	4.99
TUA G29	2.01	2.87	2.14	3.58	3.66	5.20	8.68	7.27	6.57	4.01	8.82	4.33	8.96	4.85	8.76	4.89	8.64	4.74	1.78	2.02	2.38	2.47	7.49	6.14	5.91	3.89	9.76	5.03	10.42	5.03	9.25	4.88	10.60	4.85
TUA HAG604	2.18	3.48	2.69	3.82	4.02	5.38	8.78	7.48	5.30	4.28	8.96	4.80	8.74	4.91	8.15	4.93	8.51	4.68	2.04	2.15	2.69	2.51	8.10	6.57	5.64	4.56	8.65	5.01	9.57	5.01	9.44	4.84	10.31	5.29
TUA HAG607	1.97	2.89	2.25	3.43	3.54	5.08	8.06	6.54	5.30	3.65	7.80	4.21	8.20	4.33	8.06	4.45	7.14	3.89	1.71	1.94	2.46	2.34	7.70	5.82	4.90	3.54	7.74	4.78	8.23	4.77	8.81	4.60	9.29	4.34
TUA HAG608	1.97	2.99	2.57	3.40	4.02	5.27	8.28	7.00	6.31	4.14	8.54	4.62	9.15	4.81	8.26	4.83	7.92	4.58	1.75	1.94	2.64	2.72	7.88	6.63	5.26	3.66	8.07	4.75	9.42	4.94	8.72	4.57	8.39	4.27
TUA HAG612	1.97	2.98	2.45	3.39	3.44	4.92	7.92	7.06	5.24	3.71	7.41	4.02	8.09	4.04	7.89	4.24	7.42	3.98	1.90	2.12	2.52	2.53	7.45	5.92	5.45	3.80	8.15	4.22	8.51	4.36	7.75	4.23	8.64	4.24
TUA NG301	2.28	3.77	2.73	4.59	4.50	5.78	10.09	7.99	6.02	4.40	9.49	5.32	9.74	5.07	8.75	5.37	8.16	4.47	2.18	2.49	2.73	2.95	9.19	7.60	5.89	4.25	9.22	5.55	9.88	5.61	10.02	5.46	8.94	4.99
TUA NG304	2.04	2.69	2.50	3.23	3.39	4.38	7.22	6.51	5.26	3.80	7.26	4.41	7.70	4.75	7.60	4.74	7.90	4.88	1.92	2.12	2.53	2.48	6.47	5.91	5.19	3.78	7.59	4.48	8.07	4.73	7.60	4.55	8.98	4.93
TUA NG305	1.99	2.64	2.16	2.82	3.47	5.25	7.48	6.52	5.57	3.88	7.49	3.85	8.26	4.42	7.44	4.40	7.63	4.27	1.70	2.10	2.24	2.58	6.95	5.34	5.21	3.66	7.46	4.56	9.06	4.90	8.13	4.39	8.63	4.58
TUA NG505	2.08	2.92	2.68	3.42	3.78	5.14	8.15	6.75	6.48	4.29	8.63	4.59	9.20	4.80	8.15	5.05	8.19	4.79	1.95	2.24	2.85	2.79	7.62	6.33	5.96	4.18	8.57	5.00	8.99	5.01	9.16	4.72	9.88	5.01
TUA NG506	1.77	2.94	2.35	3.30	3.55	4.91	7.56	6.47	6.34	4.31	7.60	4.23	8.02	4.38	8.03	4.44	7.81	4.28	1.88	2.13	2.46	2.62	7.00	5.56	5.79	4.11	7.73	4.50	8.17	4.64	8.21	4.53	8.73	4.59
TUA NG508	2.05	3.06	2.49	3.93	4.00	5.60	8.06	7.06	6.06	4.02	8.59	4.19	8.49	4.60	8.69	4.85	7.91	4.72	1.94	2.19	2.51	2.58	7.41	6.10	5.45	3.97	8.22	4.82	9.35	4.92	8.96	4.70	9.74	4.71
TUA NG509	2.14	2.61	2.46	3.22	3.27	4.66	7.32	6.69	5.26	3.70	8.13	3.92	9.05	4.43	7.72	4.28	6.58	3.77	1.88	1.91	2.45	2.29	7.11	5.77	5.35	3.73	8.07	4.11	8.73	4.31	8.24	4.06	8.79	3.96
TUA NG510	2.02	3.05	2.36	3.87	3.84	5.40	7.95	6.86	6.28	4.23	8.69	4.62	8.41	4.72	8.44	4.74	8.53	4.68	1.94	2.16	2.32	2.77	6.94	6.19	5.46	4.07	8.09	4.79	9.17	4.89	8.49	4.64	8.98	4.59

continued

Table S3. *Continued*

Specimen reference No.	Measurements																																	
	LI^1	WI^1	LI^2	WI^2	LI^3	WI^3	LC^1	WC^1	LP^1	WP^1	LP^2	WP^2	LP^3	WP^3	LP^4	WP^4	LM^1	WM^1	LI_2	WI_2	LI_3	WI_3	LC_1	WC_1	LP_1	WP_1	LP_2	WP_2	LP_3	WP_3	LP_4	WP_4	LM_1	WM_1
**Males**																																		
TUA NoG911	2.05	3.59	2.25	4.15	3.65	4.78	8.36	6.60	6.34	4.16	8.36	4.43	8.91	4.72	8.33	4.85	8.33	4.40	1.96	2.32	2.65	2.48	7.12	6.19	5.55	4.14	7.98	5.20	9.71	5.34	9.13	4.91	10.07	4.77
TUA ReG909	2.12	2.92	2.58	3.60	3.90	5.62	8.27	6.96	5.99	3.98	8.27	4.57	7.68	4.81	8.86	4.87	8.05	4.39	1.87	1.92	2.18	2.97	7.80	6.39	5.66	4.11	8.40	4.80	9.40	4.91	8.94	4.89	9.88	4.51
TUA ReG911	2.11	3.07	2.35	3.27	3.52	4.97	8.20	6.81	5.53	3.71	8.69	4.59	7.83	4.47	8.08	4.11	6.58	3.96	1.84	2.10	2.73	2.63	7.07	6.19	5.13	3.64	7.90	4.51	8.65	4.58	8.22	4.26	9.25	4.35
TUA ReG1004	2.17	3.09	2.66	3.77	3.88	5.21	7.79	6.64	5.93	3.85	8.12	4.58	8.79	4.97	7.96	4.67	8.15	4.84	2.07	2.35	2.73	2.86	7.16	6.18	5.38	4.06	7.80	5.12	9.33	5.23	9.06	5.09	9.17	4.89
TUA ReG1007	2.19	3.28	2.72	3.47	3.88	5.04	7.64	6.64	5.71	3.65	8.15	4.34	8.44	4.47	8.89	4.67	7.13	3.87	1.92	1.96	2.52	2.53	7.44	5.89	5.48	3.95	8.64	4.74	9.24	4.74	8.28	4.75	9.96	4.82
TUA ReG1008	2.10	2.91	2.68	3.70	3.99	5.22	8.41	7.09	5.46	4.10	7.59	4.30	8.57	4.72	7.47	4.70	7.87	4.40	1.95	2.18	2.86	2.80	7.22	6.13	5.00	4.21	7.73	4.63	8.48	4.82	8.72	4.43	9.28	4.64
TUA ReG1010	2.18	3.64	2.56	3.83	3.92	5.35	9.04	7.43	6.28	4.30	8.11	4.86	8.13	5.07	8.41	4.87	7.69	4.80	1.88	2.27	2.52	2.88	7.73	6.75	5.47	3.91	8.23	5.25	9.45	5.38	9.08	5.02	9.29	4.81
TUA RG502	1.92	2.84	2.55	3.11	3.98	5.05	8.94	7.40	5.51	3.91	8.38	4.34	8.29	4.61	8.40	4.73	7.66	4.65	1.91	2.07	2.65	2.27	7.70	6.43	5.35	3.76	8.29	4.68	9.12	4.72	8.88	4.35	9.51	4.67
TUA RG503	1.99	3.30	2.70	3.87	4.08	5.49	8.28	6.71	6.65	3.95	8.10	4.50	8.96	4.76	8.68	4.85	8.50	5.22	1.94	2.14	2.79	2.62	7.37	6.17	5.46	4.13	8.25	4.91	8.73	5.03	8.79	4.95	9.89	4.90
TUA RG504	1.88	2.71	2.26	3.35	3.72	4.94	7.71	6.60	5.72	3.67	8.52	4.21	8.43	4.52	7.82	4.21	7.02	4.35	1.86	2.00	2.51	2.53	7.41	6.21	5.77	4.21	8.15	4.84	9.28	4.74	8.23	4.58	8.53	4.48
TUA RG505	2.20	3.01	2.61	3.90	4.08	5.29	8.60	7.30	6.48	4.31	8.38	4.61	9.35	4.73	9.06	5.03	8.97	4.77	2.07	2.19	2.59	2.75	8.20	6.70	6.19	4.35	9.10	5.17	10.33	5.37	9.10	4.89	9.45	4.82
TUA RG506	2.12	3.53	2.46	4.15	4.04	5.27	9.06	7.55	6.27	4.05	8.53	4.47	9.15	4.95	9.16	5.05	8.20	4.92	2.06	2.34	2.87	2.82	7.91	6.57	5.85	4.31	8.57	5.05	9.61	5.21	9.47	5.10	10.27	5.06
TUA RG508	2.11	3.11	2.63	3.35	3.93	5.28	10.66	7.92	5.11	4.08	7.39	4.67	8.23	4.72	8.01	4.75	7.83	4.60	2.06	2.40	2.70	2.80	8.64	6.68	5.18	4.09	7.81	4.82	8.63	4.82	8.00	4.56	9.74	4.90
TUA RG509	1.91	2.39	2.41	2.90	3.39	3.58	6.99	6.39	5.16	3.88	7.08	3.74	7.50	4.03	7.15	4.18	7.12	3.81	1.68	1.79	2.21	2.22	6.69	5.45	5.36	3.63	7.07	4.09	8.37	4.10	8.02	4.03	8.11	3.75
TUA RG510	2.02	3.11	2.67	4.07	4.28	5.29	9.26	8.10	6.32	3.96	8.63	4.62	8.77	4.70	8.21	4.88	8.40	4.78	1.81	2.26	2.58	2.50	8.67	7.02	6.37	4.42	9.25	5.04	10.07	5.04	9.55	4.78	9.63	4.95
TUA RG512	2.13	3.43	2.57	3.86	3.90	5.38	9.25	7.12	6.13	4.25	8.77	4.64	9.45	4.80	8.21	4.78	8.97	4.82	2.18	2.47	2.85	3.09	8.29	6.27	6.33	4.19	9.11	5.35	9.88	5.42	9.10	5.07	10.02	5.26
TUA RG513	2.11	3.25	2.60	4.06	3.74	5.23	7.94	7.14	5.56	3.95	7.54	4.60	8.20	4.50	8.07	4.70	7.86	4.79	1.87	2.05	2.39	2.45	7.78	6.28	5.23	4.16	7.79	5.09	8.99	4.98	8.26	4.75	9.21	4.98
TUA RG516	2.07	2.81	2.54	3.17	3.69	4.83	8.19	7.03	6.09	4.25	7.61	3.97	8.14	4.40	6.32	4.27	6.82	4.51	1.90	2.20	2.50	2.64	7.84	6.25	5.23	4.05	8.01	4.35	8.72	4.56	8.15	4.31	8.65	4.92
TUA RG517	2.09	3.67	2.40	4.41	3.72	5.44	9.70	7.65	6.11	4.27	8.32	4.64	9.08	4.71	8.41	4.92	8.48	4.68	1.89	2.51	2.23	2.77	8.23	6.82	5.10	3.91	8.72	5.17	9.89	5.30	9.69	4.52	10.52	4.62
TUA RG520	1.74	3.02	2.37	3.41	3.29	4.73	7.29	6.47	5.76	4.09	8.16	4.63	7.85	4.76	7.06	4.63	7.99	4.75	1.82	2.03	2.35	2.43	7.27	5.44	5.40	3.77	7.83	5.16	7.79	4.97	7.53	4.79	8.18	4.66
TUA RG522	2.08	3.26	2.56	3.73	3.83	4.93	7.65	6.17	5.96	4.12	8.27	4.28	8.72	4.40	8.32	4.72	7.82	4.46	1.90	2.17	2.89	2.70	7.77	5.72	5.61	3.84	8.32	4.84	9.51	4.78	8.53	4.73	10.14	5.00
TUA RG526	2.09	2.92	2.72	3.38	4.22	4.85	8.15	7.52	6.10	4.51	8.71	5.41	8.45	4.90	8.00	4.61	8.30	4.66	2.22	2.33	2.79	2.69	7.30	6.17	6.15	4.45	8.67	5.19	10.13	5.18	9.13	5.06	9.96	4.41
TUA RG530	2.08	3.04	2.37	3.78	4.14	5.73	8.89	7.14	5.81	3.66	8.19	3.56	7.98	4.62	8.32	4.61	7.51	4.20	1.95	2.22	2.75	2.62	7.54	6.22	5.91	3.74	8.06	4.86	8.56	4.90	8.93	4.66	9.42	4.29
TUA RG708	2.10	2.91	2.46	3.67	3.88	5.10	8.39	7.13	6.22	3.81	7.77	4.02	9.16	4.56	8.07	4.86	8.14	4.15	1.84	2.10	2.50	2.44	7.74	6.46	5.58	3.79	8.08	4.38	8.90	4.61	8.05	4.45	9.86	4.82
TUA RG710	2.05	2.89	2.48	3.33	3.60	4.74	7.56	6.31	5.44	4.03	7.42	4.19	7.97	4.37	7.32	4.47	7.47	4.59	1.88	2.33	2.41	2.55	7.46	5.92	5.12	3.80	7.16	4.50	8.39	4.84	8.00	4.49	9.53	4.48
TUA RG804	1.84	2.93	2.58	3.38	4.03	5.11	7.52	6.86	5.44	3.78	8.06	4.21	8.36	4.69	8.32	4.84	7.67	4.44	1.91	2.13	2.62	2.90	7.46	6.14	5.56	3.87	8.04	4.73	8.79	4.97	7.97	4.77	9.57	4.88
TUA RG805	2.01	3.03	2.60	3.45	3.89	5.39	8.78	7.38	6.33	3.80	8.53	4.37	9.17	4.46	8.97	4.63	8.05	4.38	1.86	2.14	2.77	2.75	8.11	6.39	5.63	3.99	8.69	4.86	9.24	4.92	8.71	4.68	9.42	4.87
TUA RG913	2.39	2.99	2.82	3.67	4.09	5.09	8.89	7.59	5.86	4.25	7.88	4.60	9.11	5.25	8.74	5.24	9.10	5.11	2.06	2.30	2.85	2.68	7.78	6.95	5.87	4.35	8.77	5.22	10.06	5.28	9.12	5.23	10.13	5.22
TUA RG918	2.20	3.51	2.68	4.22	4.29	5.93	8.12	6.95	6.16	4.46	9.03	5.01	9.29	5.28	9.01	5.06	8.05	4.59	2.16	2.33	2.82	2.75	7.98	6.48	6.62	4.67	8.86	5.17	9.87	5.49	9.48	5.14	9.64	4.91
TUA RG919	2.15	3.02	2.51	3.58	3.85	5.57	8.34	6.89	5.64	3.98	7.73	4.04	8.42	4.43	7.95	4.72	7.56	4.46	1.92	2.02	2.45	2.38	7.76	6.11	5.07	3.99	7.56	4.76	9.31	4.86	8.15	4.57	9.52	4.60
TUA RG920	2.04	3.05	2.60	3.94	3.94	5.48	8.96	7.55	5.57	4.06	8.63	4.41	9.48	4.73	8.97	4.83	8.02	4.20	1.83	2.22	2.49	2.76	8.17	6.75	5.16	3.69	8.97	4.88	9.60	4.94	9.11	4.75	9.93	4.57
TUA RG925	1.88	2.74	2.34	3.24	3.71	4.57	6.82	5.86	5.46	3.68	7.34	4.20	7.50	4.21	7.75	4.53	7.50	3.98	1.71	2.42	2.07	2.58	5.84	5.45	4.82	3.69	7.62	4.20	7.99	4.24	7.64	4.06	8.09	4.00
TUA YG401	1.88	2.65	2.22	2.78	3.32	4.57	7.13	6.11	5.23	3.48	7.90	4.05	8.33	4.10	7.71	4.30	7.50	4.21	1.73	1.79	2.22	2.22	6.96	5.48	5.15	3.68	7.83	4.42	8.66	4.44	8.60	4.22	8.33	4.18
TUA YG402	1.97	3.20	2.50	3.55	3.81	5.00	7.92	6.81	5.49	3.81	8.18	4.37	8.81	4.41	8.06	4.66	7.98	4.62	1.94	2.20	2.54	2.55	7.62	6.13	5.74	4.05	8.23	4.85	9.02	4.88	8.65	4.74	9.68	4.82
TUA YG403	2.09	3.37	2.28	3.36	3.71	5.57	8.57	7.18	5.85	3.73	7.51	4.75	7.65	4.89	8.04	5.01	7.27	4.55	1.99	2.16	2.70	2.72	7.98	6.53	5.38	4.15	7.89	5.18	8.70	5.23	8.73	5.09	9.44	4.61
TUA YG404	2.03	2.52	2.31	3.07	3.33	4.38	7.54	6.55	5.87	4.05	7.26	4.03	7.63	4.23	7.19	4.60	7.19	4.06	1.78	1.90	2.32	2.30	6.88	5.77	5.46	3.69	7.44	4.46	8.03	4.62	7.77	4.24	8.70	4.17
TUA YG502	2.09	3.12	2.31	3.70	3.44	5.28	8.17	6.79	4.64	3.49	7.08	4.40	7.50	4.56	7.41	4.69	7.85	4.45	1.79	2.03	2.45	2.49	7.18	5.65	5.48	3.54	8.80	4.52	9.13	4.54	8.29	4.44	8.66	4.42
TUA YG503	2.04	3.05	2.85	3.75	4.51	5.96	9.05	7.58	6.17	4.13	9.10	4.86	9.64	5.08	8.04	5.36	8.63	4.73	1.73	1.93	2.40	2.36	8.16	6.62	6.10	4.52	9.27	5.44	10.13	5.26	9.17	5.23	9.35	4.86
TUA YG601	2.07	2.92	2.41	3.75	4.12	5.57	8.93	7.53	5.97	4.77	9.89	4.47	9.98	4.59	8.53	4.99	9.01	4.85	1.91	2.23	2.68	2.53	8.13	6.88	5.92	4.50	9.55	4.97	10.28	5.10	9.68	4.94	10.96	5.13
TUA YG701	2.10	3.19	2.62	3.98	3.97	5.46	7.45	6.81	5.57	4.46	7.77	4.62	8.62	4.83	8.72	4.96	8.43	5.00	2.12	2.39	2.57	2.85	7.45	6.00	5.86	4.01	8.11	5.01	8.99	5.18	8.92	4.94	9.61	5.28
TUA YG704	1.90	2.92	2.42	3.62	3.85	5.01	8.52	7.00	5.35	3.74	8.30	4.14	8.49	4.54	7.93	4.78	7.54	4.66	1.78	2.01	2.54	2.36	7.71	6.28	5.08	3.79	8.13	4.51	8.92	4.78	8.14	4.42	8.08	4.85
TUA YG906	2.05	3.63	2.54	4.13	4.19	5.80	9.35	7.69	5.74	4.13	9.26	4.69	10.30	4.66	9.59	4.82	7.32	4.44	1.79	1.98	2.78	2.68	8.40	6.23	5.45	3.93	9.38	4.66	9.84	5.12	9.74	4.89	10.10	4.58

continued

Table S3. *Continued*

Specimen reference No.	Measurements																																
	LI^1	WI^1	LI^2	WI^2	LI^3	WI^3	LC^1	WC^1	LP^1	WP^1	LP^2	WP^2	LP^3	WP^3	LP^4	WP^4	LM^1	WM^1	LI_2	WI_2	LI_3	WI_3	LC_1	WC_1	LP_1	WP_1	LP_2	WP_2	LP_3	WP_3	LP_4	WP_4	LM_1	WM_1
**Females**																																		
KUZ M9264	1.78	2.72	2.51	3.14	3.78	4.60	7.29	5.68	5.51	3.53	6.82	3.99	7.96	4.48	7.74	4.42	7.76	4.17	2.11	2.09	2.77	2.53	7.40	5.77	4.93	3.46	7.86	4.31	8.61	4.44	8.59	4.32	8.69	4.30
KUZ M9348	1.95	2.99	2.27	3.02	3.49	4.17	6.85	5.68	5.55	3.59	6.82	3.75	7.44	3.96	7.38	4.24	8.35	4.25	1.65	1.91	2.51	2.26	6.22	5.16	4.58	3.27	7.66	3.99	8.05	4.17	7.78	4.02	9.22	4.44
KUZ M9465	2.09	2.73	2.36	3.27	3.54	4.67	7.54	6.45	5.56	3.67	8.13	4.08	7.90	4.13	7.31	4.22	7.71	4.24	1.95	2.03	2.62	2.56	7.09	5.76	5.05	3.72	8.17	4.35	8.58	4.38	8.31	4.24	8.78	4.58
KUZ M9499	1.96	2.79	2.15	3.14	3.51	4.17	7.40	6.38	5.93	3.66	7.46	4.24	7.78	4.29	7.61	4.68	7.10	4.64	1.90	2.15	2.63	2.43	6.39	5.22	5.15	3.87	7.76	4.30	8.35	4.68	8.05	4.38	8.58	4.42
KUZ M9502	1.96	2.89	2.81	3.69	3.79	4.50	7.15	5.92	5.31	3.28	7.12	3.66	7.96	4.29	7.84	4.20	7.25	4.07	2.05	1.98	2.57	2.35	6.90	5.47	5.10	3.57	7.65	4.28	8.32	4.43	7.92	4.15	8.28	4.35
KUZ M9505	1.96	2.91	2.44	3.57	3.80	4.90	8.38	6.88	5.73	3.94	7.63	4.29	8.36	4.27	8.49	4.48	7.64	4.38	1.92	2.09	2.61	2.48	6.88	6.05	5.30	3.96	8.16	4.53	8.79	4.47	8.44	4.40	9.74	4.52
KUZ M9507	1.94	2.88	2.14	2.98	3.69	4.68	7.58	6.25	5.47	3.71	7.40	3.93	8.33	3.95	8.00	4.37	7.81	4.33	1.59	2.06	2.78	2.56	7.12	5.75	5.05	3.72	8.09	4.25	8.88	4.46	8.51	4.25	8.88	4.38
KUZ M9537	2.08	2.70	2.65	3.67	3.96	4.98	7.73	6.10	5.28	3.89	7.22	3.97	8.35	4.66	8.01	4.65	7.49	4.48	1.99	1.92	2.28	2.41	7.13	5.45	4.95	3.92	7.97	4.69	9.06	4.91	8.54	4.46	8.99	4.57
KUZ M9749	2.04	2.72	2.47	3.07	3.49	4.70	6.83	6.00	5.39	3.83	7.64	4.50	8.17	4.67	8.70	4.85	7.02	3.95	1.80	1.86	2.58	2.40	6.59	5.42	4.93	3.55	8.02	4.50	8.48	4.69	8.81	4.50	9.79	4.71
KUZ M9784	1.99	2.87	2.24	3.08	3.83	4.77	7.84	6.45	5.85	3.81	7.44	4.32	8.25	4.27	7.68	4.35	7.68	4.28	1.76	2.19	2.29	2.32	7.09	5.90	5.04	3.75	8.26	4.43	8.87	4.39	8.49	4.24	9.61	4.56
KUZ M9868	1.61	2.61	2.03	2.98	2.77	4.76	6.74	6.15	5.11	4.10	7.27	4.17	7.26	4.17	6.77	4.37	7.69	4.13	1.76	1.91	1.85	2.24	6.28	5.28	4.68	3.46	7.36	4.20	7.97	4.29	7.31	4.16	8.33	3.92
NSMT M28385	2.08	3.36	2.65	3.60	4.05	5.06	8.07	6.64	6.48	4.12	7.69	4.50	8.85	4.52	8.22	4.48	8.46	4.23	2.06	2.16	2.66	2.69	7.11	5.74	5.40	3.77	7.97	4.59	8.94	4.80	8.72	4.30	9.69	4.26
TUA AbG702	2.09	2.85	2.50	3.32	4.02	4.77	7.35	6.12	6.03	4.22	8.57	4.28	8.67	4.67	8.10	4.85	8.11	4.60	1.85	1.94	2.53	2.26	6.75	5.56	5.67	3.96	8.05	4.63	8.70	4.89	8.03	4.68	8.98	4.70
TUA AbG703	2.05	2.69	2.21	3.17	3.32	4.53	7.37	6.50	5.40	3.67	6.82	4.18	7.39	4.48	7.29	4.84	8.33	4.70	1.99	2.01	2.57	2.59	6.86	5.54	4.84	3.65	7.74	4.63	8.53	4.65	8.40	4.66	9.35	4.72
TUA AbG902	1.81	2.52	2.28	2.90	3.47	4.44	6.99	6.39	5.31	3.51	7.78	3.91	8.39	4.05	8.42	4.44	7.60	4.20	1.77	1.93	2.33	2.27	6.98	5.48	5.02	3.63	7.81	4.29	8.31	4.38	8.00	4.30	8.72	4.14
TUA AG2	2.11	2.53	2.60	3.36	3.67	4.99	8.30	6.77	6.04	4.34	7.99	4.40	8.51	4.97	8.80	4.76	8.16	4.77	1.95	2.13	2.56	2.65	7.52	6.31	5.41	4.24	8.81	4.95	9.19	5.20	8.66	4.65	9.38	5.03
TUA BG903	1.83	2.25	2.15	2.55	3.28	4.42	6.45	5.44	5.26	3.52	6.92	4.03	7.61	3.75	7.74	4.03	7.17	3.60	1.74	1.86	2.34	2.01	5.72	4.98	4.56	3.55	7.20	3.99	7.81	4.20	7.40	3.94	7.88	3.65
TUA BG904	2.06	2.88	2.56	3.41	3.98	5.20	8.33	6.86	6.22	3.82	7.85	4.31	8.53	4.48	8.76	4.82	8.22	4.36	1.90	1.96	2.58	2.57	7.44	5.82	5.43	3.80	8.24	4.89	8.76	4.74	9.09	4.76	9.74	4.50
TUA BG906	2.02	2.78	2.43	3.24	3.57	4.65	7.72	6.39	5.94	3.73	7.32	4.07	8.62	4.26	7.80	4.38	8.13	4.39	1.69	1.89	2.41	2.15	6.78	5.80	5.34	3.73	7.75	4.76	8.33	4.81	7.94	4.38	9.06	4.24
TUA G2	1.89	2.85	2.33	3.35	3.60	4.90	7.62	6.49	5.40	3.76	8.10	4.09	8.52	4.41	8.23	4.50	8.18	4.21	1.73	1.94	2.31	2.49	7.02	5.82	4.98	3.78	8.16	4.42	8.89	4.61	8.34	4.39	9.21	4.33
TUA G4	2.00	2.67	2.15	3.13	3.50	4.48	7.36	5.94	6.01	3.56	7.25	3.84	7.92	4.12	8.14	4.37	7.59	3.95	1.75	1.97	2.28	2.23	6.88	5.37	5.65	3.44	7.91	4.31	8.49	4.34	7.81	4.06	8.55	3.80
TUA G23	1.79	2.34	2.12	2.70	3.25	3.94	7.15	5.92	6.02	3.40	7.65	3.60	8.39	3.56	7.85	3.72	7.51	3.72	1.76	1.76	2.29	2.07	6.16	5.40	4.75	3.38	7.70	3.88	8.63	3.79	8.23	4.02	8.41	3.91
TUA HAG601	2.02	2.70	2.47	3.43	3.94	5.02	8.28	6.88	5.23	3.58	7.56	4.24	8.34	4.62	7.82	4.57	7.85	4.08	2.01	2.10	2.52	2.29	7.55	5.72	5.13	3.41	7.78	4.72	8.81	4.89	8.14	4.45	8.80	4.33
TUA HAG602	1.81	2.58	2.27	3.11	3.38	4.21	7.44	6.10	5.66	3.70	6.89	3.86	8.18	3.92	7.41	4.02	7.86	4.07	1.78	1.81	2.36	2.37	6.98	5.32	5.49	3.94	7.45	4.44	8.40	4.45	7.96	4.08	8.77	3.92
TUA HAG605	1.79	2.80	2.57	3.20	3.36	4.40	7.70	6.28	5.80	3.94	7.31	4.07	7.12	4.34	7.29	4.10	6.86	3.75	1.85	1.93	2.40	2.34	6.75	5.30	5.39	3.54	7.21	4.48	7.54	4.48	7.61	4.03	8.25	3.90
TUA HAG606	2.00	2.46	2.38	3.05	3.55	4.12	7.80	6.50	5.30	3.59	6.92	3.86	7.50	4.13	6.62	4.04	6.78	4.00	2.12	2.09	2.51	2.34	6.79	5.28	4.71	3.37	7.05	4.03	7.85	4.36	7.56	4.17	8.81	4.08
TUA HAG609	1.79	2.48	2.13	3.04	3.45	4.27	6.94	6.10	4.92	3.27	7.62	3.87	7.95	3.93	7.79	3.96	6.83	3.69	1.65	1.89	2.25	2.27	6.67	5.40	5.13	3.69	7.33	4.27	7.95	4.34	8.09	4.32	8.92	4.33
TUA HAG611	1.97	2.78	2.59	3.65	4.04	5.00	7.41	6.40	5.72	3.88	7.25	4.12	7.62	4.27	7.29	4.30	7.78	4.52	1.83	2.16	2.67	2.38	6.86	5.74	5.19	4.02	7.51	4.78	8.14	4.82	8.29	4.69	9.62	4.72
TUA NG302	1.95	2.72	2.34	3.66	3.55	4.70	7.76	6.34	5.42	3.56	7.59	3.88	8.44	4.44	8.07	4.15	7.42	4.04	1.84	1.76	2.42	2.53	6.40	5.65	4.85	3.64	7.83	4.08	8.32	4.20	7.71	4.10	8.98	4.10
TUA NG303	2.00	2.57	2.37	2.69	3.55	4.42	7.06	6.39	5.67	3.55	7.68	3.82	8.12	4.28	8.10	4.33	8.04	4.70	2.31	2.61	2.43	2.40	6.74	5.67	5.38	4.05	8.39	4.42	8.31	4.57	8.17	4.26	8.98	4.60
TUA NG306	1.91	2.85	2.25	3.31	3.79	4.44	7.81	6.45	5.18	3.77	7.67	3.87	8.66	4.18	8.17	4.19	7.95	4.16	1.91	1.94	2.28	2.47	7.01	5.48	5.47	3.97	7.91	4.31	8.64	4.37	7.92	4.30	8.39	4.24
TUA NG504	1.88	2.78	2.40	3.38	3.33	4.92	8.11	6.76	5.24	3.80	7.38	4.01	8.26	4.10	8.05	4.50	8.33	4.42	1.71	2.02	2.48	2.48	7.22	6.19	4.78	3.72	7.70	4.83	8.84	4.77	8.43	4.60	9.82	4.55
TUA ReG908	2.02	3.11	2.68	3.72	3.62	4.65	7.54	6.75	5.68	4.21	7.55	4.02	7.77	4.32	8.09	4.36	6.80	3.96	2.06	2.29	2.73	2.63	7.38	5.90	5.13	3.85	6.99	4.87	8.72	4.69	8.23	4.56	9.24	4.15
TUA ReG912	1.96	2.97	2.51	3.53	3.90	4.85	7.38	5.91	5.71	4.02	7.15	4.41	8.36	4.61	8.19	4.92	7.98	4.74	1.98	2.16	2.57	2.64	7.39	5.82	5.62	4.08	8.14	5.14	8.51	5.13	8.88	4.98	9.06	4.91
TUA ReG1001	1.95	2.97	2.62	3.72	3.84	5.41	8.66	7.17	5.83	4.09	7.47	4.30	8.52	4.64	8.46	5.01	8.53	4.83	1.88	2.10	2.50	2.61	7.61	6.35	5.88	3.92	8.14	4.85	9.26	5.10	8.40	4.87	9.68	4.90
TUA ReG1002	2.11	2.60	2.53	3.18	3.28	4.59	7.41	6.09	5.95	4.28	7.10	3.95	8.61	4.02	7.87	4.24	7.64	4.33	1.82	2.06	1.92	2.28	6.08	5.44	4.55	3.45	7.46	4.30	8.20	4.27	7.86	4.21	9.00	4.34
TUA ReG1006	2.06	2.76	2.59	3.14	3.58	4.68	8.02	6.61	5.39	3.56	7.84	4.03	8.15	4.23	7.99	4.39	6.84	4.21	2.09	2.19	2.61	2.51	7.31	5.81	5.49	3.64	7.88	4.27	8.32	4.43	8.42	4.28	9.00	4.38
TUA ReG1009	2.03	2.84	2.88	3.90	3.68	5.39	7.77	6.80	5.52	4.14	7.68	4.47	7.82	4.76	7.61	4.74	7.20	4.57	1.99	2.14	2.58	2.55	7.56	6.04	5.65	4.14	7.70	4.91	8.31	5.00	8.35	4.59	9.46	4.76
TUA ReG1011	1.91	3.04	2.34	3.54	3.67	4.81	7.17	6.14	5.56	3.92	7.54	4.07	7.75	4.44	7.33	4.62	7.67	4.30	1.68	1.94	2.54	2.46	6.87	5.61	5.50	3.79	7.77	4.78	8.47	4.95	7.87	4.59	8.91	4.48
TUA RG523	1.98	2.77	2.54	3.24	3.81	4.28	7.26	6.02	5.77	3.66	7.63	3.57	7.81	3.87	7.36	4.11	7.10	4.23	1.92	2.10	2.49	2.44	6.25	5.44	4.93	3.68	7.61	4.25	8.36	4.13	7.54	3.97	8.28	4.07
TUA RG524	1.94	2.58	2.50	3.57	3.55	4.54	7.62	6.79	5.29	3.76	7.45	4.04	7.96	4.36	7.85	4.33	7.46	4.09	1.96	2.03	2.47	2.33	7.41	6.00	5.39	3.66	7.67	4.40	8.21	4.47	7.52	4.18	8.90	4.22
TUA RG525	1.95	2.58	2.54	3.55	3.61	4.85	6.85	6.20	5.27	3.50	6.83	4.20	7.34	4.22	7.41	4.25	6.60	3.75	1.72	1.85	2.18	2.34	6.58	5.42	5.18	3.61	7.06	4.45	8.13	4.35	7.90	4.06	8.35	4.22
TUA RG528	2.05	2.93	2.38	3.17	3.58	4.45	7.04	5.97	6.12	3.80	8.28	4.04	8.42	4.26	8.27	4.41	7.44	4.11	1.69	1.88	2.54	2.33	6.67	5.34	5.56	3.63	7.76	4.39	8.11	4.61	7.77	4.02	8.32	4.07

continued

Table S3. *Continued*

Specimen reference No.	Measurements																																	
	LI^1	WI^1	LI^2	WI^2	LI^3	WI^3	LC^1	WC^1	LP^1	WP^1	LP^2	WP^2	LP^3	WP^3	LP^4	WP^4	LM^1	WM^1	LI_2	WI_2	LI_3	WI_3	LC_1	WC_1	LP_1	WP_1	LP_2	WP_2	LP_3	WP_3	LP_4	WP_4	LM_1	WM_1
**Females**																																		
TUA RG529	1.76	2.58	2.22	2.90	3.48	4.27	7.34	6.08	5.61	3.65	7.55	3.89	7.93	4.35	7.50	4.44	7.52	4.42	1.93	2.02	2.37	2.45	6.35	5.50	4.44	3.45	7.45	4.25	8.18	4.18	7.83	4.14	9.08	4.32
TUA RG533	2.04	2.77	2.48	3.37	3.80	4.53	8.15	6.40	5.54	3.73	6.92	4.03	8.07	4.29	7.93	4.49	7.37	4.20	1.71	1.90	2.45	2.41	7.44	6.04	5.58	3.80	6.74	4.88	8.78	4.98	8.46	4.64	8.94	4.21
TUA RG534	2.11	3.05	2.71	3.67	3.78	5.23	7.65	6.75	5.52	3.34	6.93	3.98	7.73	4.13	7.67	4.26	7.42	4.06	1.75	2.06	2.31	2.36	7.16	6.22	5.04	3.59	7.37	4.50	8.09	4.44	8.00	4.15	8.86	4.15
TUA RG712	1.82	2.60	2.27	2.92	3.56	4.77	7.38	6.52	5.13	3.76	7.85	3.82	7.92	4.09	7.42	4.27	7.25	3.72	2.10	2.19	2.45	2.43	7.38	5.85	4.77	2.98	7.78	4.25	8.33	4.19	7.98	4.10	8.65	4.11
TUA RG801	1.78	2.56	2.16	3.13	3.19	4.07	6.69	5.80	5.52	3.61	6.92	3.90	7.76	4.01	7.21	4.10	7.53	4.09	1.82	2.03	2.44	2.32	6.31	5.34	4.72	3.66	6.78	4.10	8.08	4.48	7.60	4.47	7.68	4.21
TUA RG802	1.89	2.51	2.48	2.78	3.55	4.49	6.98	5.95	5.62	3.39	6.98	3.68	8.29	3.82	7.76	4.10	8.09	4.26	1.76	1.88	2.58	2.21	6.85	5.22	4.76	2.88	7.72	4.16	8.55	4.34	8.22	4.25	9.05	4.28
TUA RG901	1.86	2.46	2.21	2.57	3.22	4.20	7.35	6.16	5.00	3.35	6.80	3.76	7.64	3.97	7.17	4.28	6.88	4.05	1.83	1.92	2.36	2.20	6.89	5.30	4.57	3.32	6.80	4.17	7.74	4.21	7.59	3.98	8.65	3.98
TUA RG903	2.03	2.48	2.55	2.99	3.78	4.63	7.15	5.95	4.76	3.47	6.74	3.96	7.98	4.16	7.26	4.38	7.18	3.99	1.78	1.75	2.56	2.27	6.34	5.28	4.88	3.63	7.27	4.40	8.59	4.40	8.13	4.32	8.65	4.25
TUA RG922	2.13	3.16	2.44	3.70	3.75	5.01	7.58	6.67	5.47	3.81	7.45	4.24	7.78	4.46	8.26	4.58	8.06	4.20	1.98	2.25	2.55	2.70	7.08	5.68	5.03	4.04	7.45	4.96	8.15	4.93	7.44	4.94	8.98	4.46
TUA RK510	2.02	2.39	2.50	2.94	3.66	4.67	7.18	6.28	5.56	3.37	7.22	3.91	7.78	4.07	7.14	4.22	6.62	3.98	1.79	1.78	2.43	2.35	6.60	5.75	5.52	3.59	7.62	4.37	8.05	4.52	7.77	4.31	8.11	4.11
TUA YG405	1.84	2.77	2.56	3.57	4.04	5.47	7.66	6.59	5.64	3.87	8.55	4.44	9.00	4.79	8.30	4.78	7.65	4.53	1.82	2.11	2.60	2.52	7.60	6.06	5.68	3.92	8.81	4.65	9.77	4.75	9.25	4.67	9.51	4.86
TUA YG501	2.16	2.90	2.72	3.54	4.36	5.46	8.61	6.74	5.95	4.23	8.24	4.54	8.74	4.91	7.94	4.83	8.73	5.01	2.11	2.20	3.10	2.86	7.43	6.24	5.49	3.96	8.43	4.94	9.85	5.11	8.90	4.61	8.71	4.73
TUA YG507	2.26	2.99	2.38	3.35	3.72	4.86	8.15	6.67	7.10	4.05	8.53	4.51	8.73	4.72	8.59	4.82	8.39	4.76	2.05	2.14	2.54	2.57	7.38	5.78	6.00	4.05	8.97	4.90	9.69	4.83	8.69	4.55	9.31	4.73
TUA YG602	2.27	2.85	2.73	3.33	3.86	4.91	7.53	6.83	5.33	4.05	8.06	4.22	8.96	4.43	8.23	4.78	7.54	4.50	1.87	2.10	2.85	2.58	7.35	5.87	5.32	3.90	7.69	4.58	9.00	4.80	8.37	4.92	9.37	4.67
TUA YG603	1.81	2.33	2.22	2.88	3.41	3.97	6.38	5.56	5.40	3.44	6.56	3.50	7.61	3.73	7.24	3.88	6.86	3.88	1.75	1.87	2.26	1.99	5.84	5.10	4.67	3.15	7.04	3.80	7.40	3.90	7.18	3.69	8.05	3.60
TUA YG702	2.01	2.73	2.18	3.18	3.59	4.45	7.06	6.00	5.46	3.68	7.78	4.00	8.36	3.68	7.41	3.89	7.53	4.06	1.71	1.87	2.32	2.27	6.30	5.06	4.81	3.55	7.85	4.36	8.31	4.28	7.98	4.08	8.50	4.01
TUA YG703	1.90	2.70	2.37	3.36	3.44	4.99	7.38	6.08	6.09	3.86	7.52	4.22	8.31	4.44	8.17	4.66	7.34	4.20	1.79	1.82	2.34	2.35	6.74	5.75	5.44	4.12	7.99	4.59	8.82	4.74	8.58	4.55	8.64	4.41
**Undetermined gender**																																		
HUNHM 9898	2.01	2.99	2.63	3.65	4.03	5.63	8.61	7.17	6.13	4.16	8.62	4.75	9.32	5.20	8.96	5.53	7.91	4.90	1.95	2.34	2.49	2.70	8.09	6.45	5.62	4.11	8.81	5.08	9.35	5.33	9.28	5.00	9.45	5.00
HUNHM 17231	2.03	2.62	2.39	2.94	3.85	4.62	7.72	6.86	5.90	3.82	7.94	4.12	8.61	4.37	8.14	4.63	7.05	4.36	1.86	2.06	2.85	2.29	6.86	5.82	5.38	3.70	8.27	4.50	8.91	4.79	8.02	4.46	7.85	4.46
HUNHM 46031	2.01	2.82	2.39	2.86	3.62	4.58	7.00	5.88	4.72	3.02	7.05	4.10	7.54	4.11	7.80	4.39	6.07	3.46	1.75	1.76	2.55	2.16	6.32	5.29	4.25	2.84	6.64	4.21	7.84	4.28	7.81	4.07	7.54	4.00
HUNHM 47760	1.90	2.68	2.46	3.38	3.65	4.99	8.20	6.72	5.73	3.90	7.68	4.40	8.45	4.40	7.28	4.39	8.13	4.41	1.92	2.08	2.49	2.24	6.95	5.72	5.38	3.77	7.92	4.62	8.76	4.83	8.75	4.80	9.59	4.83
KUZ M9257	2.03	3.06	2.37	3.46	3.78	5.14	7.83	6.80	5.20	3.92	7.75	4.24	8.36	4.50	8.02	4.69	8.41	4.52	2.10	2.17	2.99	2.51	6.83	5.85	5.14	3.85	8.43	4.81	8.86	4.97	8.73	4.58	9.80	4.67
KUZ M9258	2.22	3.17	2.75	3.78	3.97	5.18	8.52	6.08	6.09	3.95	8.08	4.29	8.67	4.25	8.68	4.71	7.77	4.43	2.12	2.32	2.66	2.70	7.82	6.15	5.67	4.34	8.30	4.82	9.07	4.74	8.85	4.78	10.48	4.81
KUZ M9356	1.87	2.96	2.81	3.52	3.82	4.84	7.72	6.07	5.35	3.67	7.20	4.23	7.48	4.54	7.62	4.70	7.72	4.13	2.13	2.12	2.79	2.41	6.85	5.78	5.30	3.57	7.76	4.63	8.38	4.65	8.38	4.53	8.43	4.17
KUZ M9369	2.16	3.16	2.47	4.24	4.74	5.63	8.98	7.41	6.47	4.12	8.13	4.53	8.69	4.91	7.94	4.90	7.25	4.77	2.10	2.18	2.64	2.78	7.88	7.34	5.51	3.90	8.63	5.02	9.53	5.14	8.33	4.94	9.21	4.82
KUZ M9374	2.10	3.45	2.51	4.14	3.80	4.80	7.51	6.65	5.86	3.76	7.38	4.16	8.44	4.27	8.34	4.67	8.22	4.83	2.00	2.19	2.58	2.70	6.59	5.71	5.34	3.83	7.95	4.34	8.57	4.53	8.69	4.67	8.70	4.39
KUZ M9395	2.19	2.93	2.30	3.11	3.56	4.55	9.29	7.11	5.88	3.54	8.18	4.18	8.50	4.50	7.93	4.69	8.31	4.80	1.65	1.97	2.42	2.39	7.83	6.29	5.61	3.79	8.24	4.79	8.96	4.93	8.45	4.67	9.52	4.92
KUZ M9396	1.91	2.68	2.21	3.17	3.79	4.79	7.71	6.41	5.79	4.17	7.49	4.38	7.69	4.77	7.06	4.65	7.48	4.31	1.91	2.06	2.62	2.49	6.85	5.64	5.20	4.00	7.66	4.79	8.47	5.01	7.98	4.60	8.72	4.62
KUZ M9426	1.94	3.11	2.43	3.58	3.98	4.93	7.78	6.46	6.21	3.93	8.28	4.50	8.38	4.80	8.06	4.69	8.54	4.70	1.76	2.03	2.40	2.23	7.41	5.92	5.44	4.06	8.64	4.62	9.38	4.60	8.61	4.47	10.08	4.80
KUZ M9596	1.92	3.15	2.30	3.35	3.80	5.20	7.51	5.63	5.30	3.49	7.90	3.94	8.62	4.22	8.28	4.40	6.97	4.24	2.09	2.10	2.56	2.62	7.04	5.15	5.41	3.67	8.20	4.43	8.73	4.52	8.12	4.39	8.95	4.35
KUZ M10298	1.80	2.68	2.36	3.18	3.48	4.79	6.72	6.03	4.75	3.42	7.06	3.71	7.42	4.02	7.23	4.08	6.63	4.05	1.82	1.88	2.42	2.14	6.55	5.13	4.84	3.44	7.38	4.25	7.82	4.15	7.56	4.01	7.96	4.14
KUZ M10300	2.06	2.90	2.58	3.48	3.34	4.94	7.67	6.26	6.17	3.88	7.75	4.19	8.08	4.60	7.71	4.75	8.64	4.75	2.02	2.20	2.58	2.70	7.19	5.43	5.32	4.02	8.01	4.77	8.96	4.73	8.25	4.66	10.03	4.79
KUZ M10305	2.16	3.07	2.78	3.65	3.66	4.53	7.46	6.30	4.87	3.61	7.20	4.24	8.29	4.32	7.93	4.56	7.60	3.86	1.94	2.11	2.48	2.45	7.00	5.68	5.10	3.60	7.49	4.68	8.61	4.55	8.65	4.50	9.76	4.49
KUZ M10306	1.90	2.86	2.40	3.68	3.45	4.78	7.37	6.32	5.34	3.36	7.84	3.62	8.11	3.88	7.63	3.99	6.97	3.75	1.85	2.07	2.31	2.54	6.27	5.50	4.74	3.52	7.55	4.18	8.04	4.07	7.60	3.82	8.43	3.84
KUZ M10307	1.96	2.51	2.32	2.94	3.21	4.12	6.96	5.64	5.27	3.52	7.00	3.78	7.35	4.06	7.18	4.15	6.83	3.73	1.55	1.58	2.26	2.23	6.15	5.03	4.87	3.40	7.33	4.02	8.00	3.79	7.93	3.98	8.14	3.85
KUZ M10308	1.97	2.50	2.15	3.04	3.38	4.90	7.44	6.92	5.02	3.42	7.24	3.91	7.93	4.14	7.48	4.06	6.50	4.08	1.65	1.86	2.30	2.25	7.00	5.74	4.75	3.47	7.39	4.25	8.14	4.34	7.37	4.17	8.20	4.10
KUZ M10309	1.75	2.54	2.44	3.12	3.74	4.00	6.84	5.75	5.35	3.77	7.48	3.62	7.65	3.93	7.10	4.05	7.25	3.93	1.71	1.83	2.46	2.26	6.41	5.17	5.04	3.68	7.40	4.27	7.97	4.22	7.29	4.09	7.78	4.09
KUZ M10311	2.05	3.09	2.64	3.78	3.91	5.10	8.28	7.00	5.81	3.90	7.80	4.28	8.26	4.41	8.00	4.59	7.91	4.60	2.16	2.43	2.77	2.75	7.09	6.26	5.94	4.11	8.69	4.69	8.90	4.88	8.27	4.73	9.04	4.59
KUZ M10312	1.84	3.13	2.43	3.60	3.78	4.93	7.94	6.43	5.62	3.80	8.21	4.36	8.51	4.36	8.59	4.45	7.49	4.38	1.93	2.25	2.58	2.49	7.77	6.15	5.10	3.72	7.98	4.22	8.94	4.55	8.85	4.46	9.17	4.37
KUZ M10313	1.72	2.86	2.33	3.49	3.39	4.39	7.62	6.18	5.04	3.30	7.66	3.83	8.10	3.86	8.02	4.31	7.62	4.01	1.98	2.01	2.42	2.40	6.90	5.59	5.16	3.33	7.47	4.39	8.27	4.50	8.01	4.31	8.72	4.27
KUZ M10314	2.16	3.45	2.52	3.66	3.86	5.38	8.84	7.05	6.36	4.07	7.87	4.17	8.72	4.39	9.02	4.64	8.32	4.87	1.89	2.21	2.66	2.83	8.30	6.55	5.67	4.04	8.61	4.56	10.04	4.82	9.12	4.50	10.52	4.86
KUZ M10315	1.99	3.02	2.42	3.51	3.58	5.04	7.71	6.19	5.80	3.81	8.50	3.94	8.80	3.99	7.87	4.09	7.53	4.03	2.06	2.15	2.58	2.46	6.81	5.63	4.89	3.91	7.69	4.60	8.80	4.67	8.73	4.31	8.91	3.98

continued

Table S3. *Continued*

Specimen reference No.	Measurements																																	
	LI^1	WI^1	LI^2	WI^2	LI^3	WI^3	LC^1	WC^1	LP^1	WP^1	LP^2	WP^2	LP^3	WP^3	LP^4	WP^4	LM^1	WM^1	LI_2	WI_2	LI_3	WI_3	LC_1	WC_1	LP_1	WP_1	LP_2	WP_2	LP_3	WP_3	LP_4	WP_4	LM_1	WM_1
**Undetermined gender**																																		
KUZ M10317	1.88	3.22	2.33	4.07	3.44	4.94	7.81	6.68	5.01	3.69	6.86	4.42	7.91	4.56	8.48	4.72	7.22	3.86	1.71	2.25	2.39	2.58	7.08	6.32	4.65	3.37	7.90	4.68	8.69	4.81	8.58	4.73	8.74	4.50
KUZ M10318	2.08	2.78	2.58	3.33	3.58	4.50	7.73	6.08	5.37	3.73	7.32	3.94	8.54	4.16	8.26	4.43	7.34	4.33	1.84	2.08	2.57	2.50	6.98	5.78	5.19	3.74	7.43	4.65	8.81	4.80	8.62	4.60	9.22	4.54
KUZ M10319	1.79	2.58	2.44	3.07	3.90	4.68	7.77	6.36	5.59	3.90	6.95	4.46	8.30	4.82	7.17	4.49	7.57	4.41	1.75	1.94	2.59	2.38	6.78	5.49	5.14	3.96	7.63	4.70	8.01	4.80	7.60	4.61	8.44	4.46
KUZ M10321	1.95	2.87	2.58	3.78	3.87	4.92	8.03	6.27	6.23	3.60	8.14	3.79	8.70	4.13	8.78	4.28	6.86	3.83	2.01	2.14	2.46	2.38	7.17	6.15	5.24	3.82	8.48	4.14	9.54	4.27	9.07	4.32	9.21	4.09
KUZ M10322	1.89	2.85	2.15	3.05	3.21	4.11	7.11	6.43	5.44	3.13	7.93	3.66	8.80	4.05	8.22	4.25	6.48	3.83	1.75	1.99	2.22	2.25	6.95	5.87	4.85	3.58	8.28	4.38	8.58	4.54	8.38	4.33	8.96	4.36
KUZ M10323	2.14	3.38	2.28	3.34	3.45	4.77	7.41	6.35	5.46	3.50	7.97	4.30	8.42	4.61	8.64	4.57	6.67	3.98	1.64	1.99	2.21	2.28	6.75	5.65	5.26	3.72	7.97	4.41	8.67	4.83	8.35	4.51	9.23	4.73
KUZ M10324	2.37	2.93	2.42	3.70	3.70	4.49	8.14	6.72	5.02	3.52	7.10	4.19	8.16	4.28	7.73	4.38	7.16	4.03	1.74	1.94	2.47	2.41	7.08	5.98	5.01	3.65	7.64	4.56	8.54	4.56	7.79	4.40	8.38	4.20
KUZ M10325	2.03	3.10	2.41	3.58	3.35	5.16	7.41	6.10	5.67	3.62	6.81	3.62	7.58	3.92	7.30	4.15	7.22	4.12	1.80	2.08	2.41	2.38	6.95	5.37	5.02	3.35	7.27	4.20	7.93	4.33	7.55	4.11	8.16	4.12
KUZ M10327	2.12	3.15	2.74	3.70	3.89	5.12	8.17	6.66	5.84	3.88	7.46	4.32	8.58	4.52	7.92	4.73	7.41	4.59	1.82	1.88	2.30	2.45	7.77	6.30	5.24	3.97	7.75	4.81	8.79	4.70	8.50	4.67	9.56	4.57
KUZ M10328	2.09	3.10	2.52	3.44	3.95	5.19	8.88	6.87	5.95	4.45	8.80	4.30	9.29	4.54	8.58	4.63	7.86	4.29	1.70	1.98	2.30	2.38	7.53	6.37	5.12	4.01	8.48	4.49	9.24	4.47	8.44	4.55	9.24	4.38
KUZ M10329	1.98	3.03	2.55	3.75	3.63	4.83	8.26	6.70	5.12	3.84	7.44	4.31	8.23	4.51	8.15	4.60	6.84	4.28	1.83	2.02	2.52	2.69	7.14	6.00	5.23	3.79	7.67	4.73	8.80	4.84	8.56	4.61	9.15	4.65
KUZ M10331	1.91	2.76	2.23	3.11	3.28	4.43	7.32	6.04	5.83	3.55	8.28	3.76	8.86	3.80	7.58	3.99	7.68	4.03	1.64	1.78	2.28	2.19	6.54	5.16	4.83	3.50	6.78	4.42	8.37	4.48	7.78	4.25	8.82	4.11
KUZ M10332	2.06	3.18	2.46	3.67	3.34	4.89	8.00	6.81	5.33	3.53	8.32	3.99	9.27	4.20	7.78	4.30	7.52	4.32	1.88	2.09	2.49	2.64	7.58	6.08	5.16	3.56	8.75	4.43	9.40	4.55	8.78	4.25	9.15	4.36
KUZ M10333	1.79	2.88	2.21	3.18	3.43	4.91	7.57	5.84	5.49	3.60	8.27	3.94	9.32	4.12	7.51	4.16	7.52	4.39	1.74	1.90	2.33	2.28	6.84	5.06	5.08	3.55	8.52	4.35	9.15	4.07	8.32	4.12	9.10	4.25
KUZ M10334	2.01	2.79	2.45	3.26	3.76	4.77	7.76	6.59	5.40	3.98	7.68	4.18	8.28	4.54	7.26	4.74	7.29	4.55	1.90	2.05	2.58	2.38	6.60	5.77	5.73	4.28	7.57	4.76	8.01	4.81	7.68	4.81	7.86	4.89
KUZ M10335	2.08	3.09	2.25	3.65	3.37	5.50	7.47	5.96	5.44	4.20	8.01	3.85	8.12	4.14	8.47	4.58	8.32	4.06	1.72	1.90	2.16	2.34	7.46	5.81	5.03	3.84	8.14	4.12	8.68	4.25	8.60	4.35	9.25	4.04
KUZ M10336	2.18	3.33	2.66	3.41	3.86	5.00	7.64	6.78	6.09	4.16	7.63	4.71	8.74	4.62	7.91	4.77	8.04	4.25	1.94	1.98	2.55	2.42	6.85	5.98	5.61	4.41	8.27	4.87	9.16	4.90	8.32	4.61	9.54	4.73
NSMT KK27	1.83	2.99	2.79	3.82	4.10	4.86	7.80	6.66	6.00	3.82	8.60	4.55	9.65	4.53	8.31	5.15	8.08	4.97	1.92	2.20	2.72	2.65	6.93	6.16	5.91	4.24	8.27	4.74	10.22	4.78	9.95	4.43	8.98	4.60
NSMT KK96	2.32	2.58	2.68	3.02	3.58	4.28	7.89	6.47	5.42	3.64	8.20	4.04	8.92	4.24	8.54	4.40	7.40	4.01	1.84	1.87	2.60	2.33	7.16	5.90	5.73	4.38	8.66	4.27	9.17	4.37	8.69	4.11	9.20	4.05
NSMT KK144	1.89	2.51	2.48	3.06	3.54	4.82	7.12	5.99	6.11	3.77	7.89	3.64	8.56	4.02	8.40	4.43	7.66	4.13	1.78	2.04	2.67	2.44	6.45	5.36	5.02	3.67	7.64	4.34	8.48	4.46	7.90	4.30	8.53	4.28
NSMT M19758	2.04	3.00	2.26	3.76	3.50	5.07	8.42	6.76	6.28	4.40	7.51	4.41	8.47	4.28	8.35	4.61	9.00	4.77	1.78	2.33	2.44	2.50	7.02	6.37	5.46	3.82	8.61	4.60	9.00	4.65	8.12	4.34	9.12	4.61
NSMT M19759	2.01	3.17	2.32	3.57	3.53	4.86	8.08	6.61	6.41	3.96	7.22	4.19	8.08	4.41	8.61	4.59	7.80	4.21	1.90	2.30	2.56	2.81	7.08	6.24	5.68	4.00	7.95	4.62	8.76	4.66	7.86	4.32	8.90	4.44
NSMT M19761	1.81	2.87	2.48	3.30	3.19	4.65	7.72	6.25	5.67	3.81	7.54	4.11	8.03	4.31	7.53	4.40	8.04	4.52	1.86	2.09	2.38	2.53	6.85	5.58	5.40	4.18	8.08	4.44	8.65	4.67	8.53	4.45	8.64	4.61
NSMT M19763	2.12	3.09	2.25	3.36	3.69	4.99	8.00	6.46	6.46	4.26	8.21	4.43	8.71	4.61	8.19	4.67	9.07	4.56	1.79	2.03	2.66	2.46	7.27	6.00	6.22	4.51	8.63	5.25	9.81	4.76	8.76	4.71	9.61	4.55
TUA BG901	1.77	3.10	2.32	3.33	3.38	4.97	7.11	5.99	5.44	3.79	7.07	4.09	7.15	4.10	7.18	4.04	6.77	3.68	1.96	2.00	2.28	2.38	6.65	5.67	5.08	3.49	7.13	4.16	7.83	4.13	7.73	4.01	8.56	3.94
TUA YG505	1.92	2.75	2.42	3.26	3.62	5.47	8.85	6.34	5.63	3.95	8.45	4.15	9.49	4.42	8.37	4.48	7.91	4.38	1.95	2.18	2.47	2.56	7.39	6.14	4.93	3.46	7.89	4.99	8.57	5.09	8.00	4.94	8.91	4.95
TUA YG506	1.88	3.12	2.33	3.52	4.07	4.90	8.15	6.61	6.45	3.92	8.10	4.32	9.06	4.64	8.78	5.14	8.72	5.02	2.01	2.16	2.60	2.66	7.64	5.91	5.63	4.10	8.32	4.64	8.90	5.05	8.56	4.67	9.78	5.13

Table S4. Measurement values for *Histriophoca fasciata*. All values are in millimeters. Measurement abbreviations: L, length of the tooth crown (maximum linear mesiodistal distance across the tooth crown); W, width of the tooth crown (maximum linear vestibulolingual distance across the tooth crown perpendicular to the length); I, incisor; C, canine; P, premolar; M, molar; superscript and subscript numbers indicate positions of upper and lower teeth, respectively. Institutional abbreviations: HUM, Hokkaido University Museum, Hokkaido University, Sapporo, Japan; HUNHM, Botanic Garden, Hokkaido University, Sapporo, Japan; KUZ, Kyoto University Museum, Kyoto University, Kyoto, Japan; NSMT, National Museum of Nature and Science, Tokyo, Japan; TUA, Laboratory of Aquatic Management, Department of Aqua Bioscience and Industry, Faculty of Bioindustry, Tokyo University of Agriculture, Abashiri, Japan.

Specimen reference No.	Measurements																																
	LI^1	WI^1	LI^2	WI^2	LI^3	WI^3	LC^1	WC^1	LP^1	WP^1	LP^2	WP^2	LP^3	WP^3	LP^4	WP^4	LM^1	WM^1	LI_2	WI_2	LI_3	WI_3	LC_1	WC_1	LP_1	WP_1	LP_2	WP_2	LP_3	WP_3	LP_4	WP_4	LM_1	WM_1
**Males**																																		
HUNHM 17216	1.88	2.19	2.14	2.84	3.16	3.64	5.81	5.11	4.36	3.63	5.06	3.28	5.32	3.45	5.19	3.35	3.81	2.22	1.40	1.44	1.65	1.72	5.00	4.00	4.14	3.28	5.62	3.50	5.94	3.65	6.19	3.53	5.49	3.12
HUNHM 17217	2.04	2.32	2.33	3.21	3.55	3.91	6.54	5.53	4.65	4.04	5.16	3.62	6.46	3.99	6.01	3.89	5.57	3.95	1.22	1.67	1.72	1.97	5.91	5.13	4.09	3.34	5.89	3.88	6.44	4.28	6.60	4.24	6.14	4.16
HUNHM 17246	1.86	2.19	2.12	2.30	3.18	3.40	6.62	4.98	4.82	3.72	5.74	3.28	6.83	3.93	6.94	4.11	5.85	3.13	1.36	1.95	1.78	2.00	6.08	4.42	4.34	3.32	6.29	4.09	7.76	4.29	7.28	4.38	7.64	3.82
HUNHM 17249	1.69	1.89	1.92	2.24	2.74	3.40	5.94	4.90	4.00	2.92	4.37	2.72	4.88	3.17	4.80	3.16	4.74	2.80	1.21	1.38	2.10	2.00	4.96	4.05	3.11	2.62	4.77	3.29	5.86	3.56	5.31	3.30	5.60	3.23
KUZ M9397	1.59	1.88	1.67	2.01	2.66	3.01	5.02	4.13	3.78	2.74	4.25	2.85	5.05	3.07	4.19	2.91	4.12	2.46	1.32	1.12	1.57	1.52	4.43	3.68	3.12	2.60	4.43	3.05	4.73	3.09	4.71	3.13	4.57	2.55
KUZ M9398	1.71	2.60	1.89	2.74	3.19	4.01	5.30	4.49	3.92	3.17	5.03	3.19	5.28	3.39	5.41	3.26	5.23	2.84	0.89	1.48	1.43	1.74	4.75	3.78	3.46	2.77	4.64	3.45	5.53	3.71	5.95	3.71	5.56	3.12
KUZ M9401	1.75	1.96	2.11	2.61	3.38	3.67	5.07	4.48	3.60	3.02	3.67	3.34	5.26	3.50	5.00	3.44	4.62	3.36	1.26	1.28	1.71	1.53	5.18	4.23	3.55	2.94	4.58	3.40	5.33	3.50	5.13	3.63	5.58	3.28
KUZ M9407	1.76	2.45	1.84	3.11	2.84	4.19	6.01	4.82	3.58	3.16	4.94	3.90	5.24	3.85	5.53	3.83	4.60	2.80	1.00	1.33	1.47	1.65	5.32	3.98	3.75	3.02	5.04	4.00	5.99	4.04	5.67	3.63	6.24	3.42
KUZ M9409	1.65	1.91	2.01	2.68	2.83	3.32	6.26	4.38	3.58	2.86	4.57	3.40	5.38	3.47	5.20	3.50	4.82	2.75	1.22	1.48	1.76	1.76	5.43	3.47	3.02	2.53	4.31	3.31	5.36	3.27	5.63	3.26	5.73	3.04
KUZ M9425	1.86	2.06	1.87	2.28	2.51	2.74	4.94	4.03	4.10	2.98	4.55	3.04	5.37	3.09	4.94	3.22	4.41	2.50	1.14	1.26	1.74	1.87	4.45	3.48	3.01	2.44	4.51	3.44	5.26	3.44	5.28	3.54	5.57	2.89
KUZ M9454	1.81	2.47	2.02	2.66	3.10	3.86	6.54	5.62	4.67	3.91	5.33	3.75	5.59	3.80	5.76	3.72	5.30	3.11	0.88	1.42	1.69	1.95	5.43	4.73	3.77	3.32	5.23	3.72	5.99	3.80	6.18	3.91	6.14	3.47
KUZ M9460	1.66	2.23	1.69	2.50	2.96	3.88	5.85	5.15	4.50	3.75	5.09	3.28	6.09	3.62	5.96	3.25	4.99	2.64	1.14	1.39	1.75	1.98	5.33	4.42	3.90	3.28	5.08	4.01	6.39	4.10	6.14	4.02	4.93	3.40
KUZ M9466	1.43	1.86	1.87	2.09	2.35	2.97	4.54	3.80	3.65	2.95	3.83	2.50	4.75	2.97	4.60	2.98	4.04	2.70	0.93	1.33	1.20	1.54	4.34	3.57	3.12	2.62	4.19	2.98	5.05	2.98	5.03	3.13	3.62	2.88
KUZ M9493	1.43	1.98	1.70	2.36	2.80	3.49	6.24	5.00	3.46	2.78	4.50	3.04	5.29	3.42	5.22	3.18	4.92	2.66	1.01	1.46	1.39	1.79	5.47	4.28	3.24	2.41	4.90	3.54	5.57	3.69	5.58	3.52	5.29	2.78
KUZ M9559	2.06	2.61	2.12	3.40	3.18	3.72	6.17	4.96	4.92	4.21	5.76	4.47	5.95	4.41	6.39	4.28	5.38	2.91	1.18	1.44	1.75	1.81	6.13	4.28	4.36	3.69	6.15	4.27	6.73	4.57	6.59	4.54	6.48	3.89
KUZ M9572	1.98	2.60	2.36	2.79	3.00	3.92	5.66	5.03	4.49	3.99	5.55	3.67	6.53	3.77	6.01	3.69	4.70	2.79	1.20	1.23	1.38	1.35	5.31	4.10	3.94	3.17	5.72	3.78	6.77	4.16	6.84	4.08	6.13	3.42
KUZ M9575	2.19	2.25	2.37	2.61	3.32	3.78	5.45	4.88	4.77	3.80	4.87	3.27	5.19	3.91	5.39	3.86	5.18	3.25	1.10	1.26	2.17	1.91	4.70	4.45	4.22	3.46	5.27	4.06	5.51	4.13	6.06	3.76	5.47	3.66
KUZ M9577	1.76	2.00	1.82	2.17	3.27	4.21	5.64	5.15	4.36	4.11	4.65	3.93	5.34	4.09	5.47	4.05	4.96	3.38	1.19	1.46	1.83	1.85	5.45	4.76	3.95	3.38	4.90	4.16	5.25	4.21	5.25	3.91	4.90	3.61
KUZ M9605	1.91	2.52	2.21	3.01	3.11	3.36	5.25	4.37	4.51	3.41	5.39	3.41	5.24	3.30	5.24	3.23	4.52	2.43	1.25	1.41	1.55	2.06	5.83	3.69	3.45	2.44	5.62	3.35	6.44	3.64	5.93	3.49	5.88	2.57
KUZ M9608	1.84	2.25	1.99	2.37	2.66	3.08	4.97	4.36	3.44	3.15	4.93	2.99	6.10	3.28	5.19	3.08	4.61	2.93	1.27	1.58	1.69	1.73	4.67	3.92	3.29	2.61	5.25	3.26	6.23	3.34	5.73	3.23	5.97	3.35
KUZ M9619	1.84	1.88	1.98	2.28	2.65	2.89	4.56	4.13	3.30	2.65	4.06	2.70	4.55	2.75	4.43	2.91	4.11	2.18	0.75	1.24	1.11	1.51	3.97	3.51	2.78	2.22	4.05	2.87	4.93	3.26	5.30	3.23	5.42	2.75
KUZ M9639	1.75	1.89	1.92	2.34	2.57	3.05	4.85	4.31	4.26	3.10	4.63	2.90	4.96	2.90	4.46	2.86	4.83	2.74	1.12	1.10	1.30	1.22	4.14	3.85	3.55	2.72	4.40	3.06	5.37	3.21	5.11	2.91	4.92	2.53
KUZ M9640	1.76	2.03	1.81	2.23	2.86	3.83	6.19	4.57	4.60	3.38	5.28	3.67	5.61	3.57	5.07	3.33	4.50	2.59	1.25	1.64	1.42	1.80	5.41	4.14	3.84	2.77	5.26	3.46	6.45	3.84	6.56	3.96	5.68	3.13
KUZ M9645	1.75	1.96	1.93	2.41	2.86	3.48	4.94	4.66	4.15	3.43	5.12	3.31	5.18	3.31	5.02	3.19	4.36	2.71	1.10	1.46	1.71	1.83	4.74	3.73	3.12	2.60	4.86	3.54	5.58	3.56	5.63	3.48	5.00	3.10
KUZ M9647	1.91	2.12	1.96	2.45	2.73	2.89	5.49	4.48	4.30	3.04	4.16	2.89	5.52	3.07	4.78	3.21	4.21	3.06	1.17	1.62	1.64	1.73	4.92	3.88	3.49	2.79	4.10	3.26	5.18	3.72	5.20	3.21	4.68	3.18
KUZ M9657	1.97	2.53	2.28	3.39	3.31	4.02	5.42	4.87	3.60	2.83	4.60	2.94	5.08	3.36	5.16	3.24	4.73	2.74	1.24	1.56	1.79	1.86	4.99	4.51	3.49	2.83	4.93	3.17	5.54	3.08	6.20	3.03	5.87	2.89
KUZ M9659	1.28	1.74	1.50	2.00	2.23	3.10	6.04	4.50	3.48	2.89	4.78	2.80	5.62	3.11	5.75	2.63	5.34	2.67	0.90	1.26	1.05	1.46	5.13	3.94	3.28	2.59	5.09	3.15	5.86	3.31	5.53	3.20	4.85	3.07
KUZ M9665	1.76	1.85	1.92	2.25	2.93	3.27	5.37	4.69	4.66	3.64	5.00	2.96	5.05	3.08	5.10	3.26	4.99	2.72	1.15	1.39	1.45	1.50	4.64	4.14	4.42	3.09	5.61	3.54	6.19	3.36	6.54	3.33	5.59	3.06
KUZ M9671	1.98	2.49	2.30	2.91	3.17	3.60	5.86	4.27	5.34	3.17	6.12	3.42	5.52	3.05	5.60	3.41	5.25	3.18	1.53	1.88	2.32	2.94	5.58	3.76	3.81	3.03	5.37	3.26	5.96	3.28	6.25	3.42	6.46	3.32
KUZ M9680	1.92	2.00	2.34	2.54	3.08	3.74	5.44	4.86	4.41	3.48	5.37	3.59	5.33	3.47	5.36	3.53	4.41	2.56	1.15	1.39	1.97	1.96	5.39	4.25	3.50	3.08	4.95	3.65	5.33	3.77	6.00	3.79	5.48	3.21
KUZ M9682	2.10	2.30	2.28	2.82	3.21	3.96	5.58	4.82	4.21	3.40	3.98	2.55	5.87	3.12	4.84	2.99	4.85	2.88	0.93	1.50	1.31	1.56	4.94	4.07	3.94	3.11	5.07	3.40	6.26	3.42	5.80	3.16	6.07	3.03
KUZ M9685	1.85	2.16	2.04	2.70	3.06	3.72	5.93	4.97	4.12	3.15	5.01	3.25	5.06	3.22	5.15	3.37	5.35	3.26	1.32	1.48	1.81	1.81	5.09	4.16	3.47	2.82	5.15	3.56	5.64	3.75	5.58	3.45	6.09	3.33
KUZ M9688	1.79	2.02	2.00	2.28	2.92	3.10	5.40	4.30	3.64	2.92	4.07	3.42	4.09	3.13	4.74	3.77	4.58	2.84	1.15	1.32	1.89	1.64	4.88	3.77	3.78	2.96	4.33	3.22	5.14	3.40	4.83	3.33	5.17	3.03

continued

Table S4. *Continued*

Specimen reference No.	Measurements																																	
	LI^1	WI^1	LI^2	WI^2	LI^3	WI^3	LC^1	WC^1	LP^1	WP^1	LP^2	WP^2	LP^3	WP^3	LP^4	WP^4	LM^1	WM^1	LI_2	WI_2	LI_3	WI_3	LC_1	WC_1	LP_1	WP_1	LP_2	WP_2	LP_3	WP_3	LP_4	WP_4	LM_1	WM_1
**Males**																																		
KUZ M9711	1.70	2.25	2.30	3.05	3.06	3.62	5.36	4.17	3.92	3.14	5.21	3.27	5.75	3.40	5.47	3.35	5.04	2.72	1.19	1.50	1.58	2.08	5.05	3.51	3.77	2.62	5.49	3.36	5.95	3.56	5.42	3.30	5.77	3.00
KUZ M9720	1.76	2.08	1.82	2.32	2.87	3.30	5.29	4.62	3.75	3.11	4.36	3.20	5.01	3.32	5.18	3.30	4.85	2.67	1.09	1.34	1.43	1.88	4.81	4.08	3.38	2.90	4.12	3.26	5.31	3.80	5.39	3.49	4.87	2.89
KUZ M9724	1.70	1.76	2.06	2.02	2.86	3.03	4.81	4.42	4.32	2.99	4.44	3.38	5.14	3.54	4.89	3.56	4.24	2.75	1.06	1.20	1.41	1.50	4.05	3.69	3.76	3.05	4.78	3.65	5.25	3.80	5.29	3.70	5.40	3.01
KUZ M9770	2.28	2.39	2.59	2.71	3.82	4.07	6.15	5.46	4.61	3.70	5.44	3.52	6.18	3.55	6.00	3.66	5.28	3.28	1.04	1.36	2.10	2.10	5.28	4.78	3.84	3.45	5.85	4.15	6.67	3.98	6.80	3.90	6.14	3.20
KUZ M9771	1.61	1.78	1.83	2.22	2.75	3.10	5.14	4.23	3.60	2.65	4.31	2.80	4.97	2.88	5.27	2.89	4.13	2.29	0.95	1.32	1.60	1.67	4.71	3.71	3.35	2.53	5.05	3.32	5.50	3.42	5.29	3.34	4.95	2.68
KUZ M9801	1.73	1.80	1.95	2.45	2.67	3.32	5.33	4.21	3.81	2.83	3.79	2.44	4.87	3.10	4.47	2.95	3.88	2.44	0.62	1.04	1.24	1.47	5.05	3.63	3.33	2.46	4.73	2.79	4.98	3.10	4.68	3.05	4.58	2.72
KUZ M9808	1.76	1.96	2.07	2.54	2.86	3.92	5.18	4.43	3.96	3.20	4.98	3.20	5.18	3.48	5.66	3.34	4.59	2.48	1.08	1.33	1.80	1.78	4.81	3.75	3.77	3.05	5.55	3.53	5.88	3.80	6.06	3.66	5.94	2.94
KUZ M9813	1.78	2.02	1.91	2.26	2.97	3.39	5.68	4.74	4.10	3.28	4.70	3.35	5.19	3.74	4.56	3.37	4.15	2.94	0.95	1.56	1.55	1.70	5.29	4.06	3.63	2.88	5.14	3.71	5.50	3.66	5.16	3.71	5.45	3.15
KUZ M9823	1.92	2.88	2.71	3.10	3.77	3.96	5.94	5.02	4.51	3.60	4.89	3.49	5.72	3.95	5.83	3.54	5.05	2.98	1.34	1.70	2.34	2.17	5.51	4.37	3.77	2.92	5.36	3.77	5.89	3.96	5.90	3.75	5.60	3.30
KUZ M9856	1.86	2.18	2.10	2.59	2.90	2.96	5.22	4.46	3.73	3.23	3.84	2.93	4.70	3.20	4.80	3.18	4.35	2.65	1.16	1.37	1.68	1.65	4.48	3.76	3.02	2.32	4.23	3.35	4.81	3.24	4.84	3.51	5.33	3.15
KUZ M9858	1.92	2.29	2.26	2.65	3.13	3.61	5.28	4.80	3.97	3.32	4.90	3.04	5.75	3.24	5.69	3.04	5.52	3.12	1.07	1.86	1.36	2.34	4.63	3.81	3.71	2.68	5.08	3.23	6.06	3.58	5.88	3.53	5.51	3.30
KUZ M9861	1.29	1.60	1.68	2.21	2.89	3.03	5.40	4.38	4.04	3.30	4.26	3.12	5.04	3.38	5.55	3.35	4.28	2.61	0.72	0.90	1.31	1.26	5.09	3.91	3.46	2.65	4.92	3.17	5.24	3.21	5.52	3.29	5.11	2.63
KUZ M9875	1.61	1.96	1.75	2.21	2.42	3.43	4.87	4.16	3.78	3.02	4.21	2.51	4.82	2.85	4.34	2.76	3.76	2.59	1.13	1.28	1.42	1.47	4.36	3.85	3.46	2.67	4.74	3.03	4.84	3.22	4.82	3.02	4.71	2.83
KUZ M9876	1.30	1.70	1.62	2.07	2.27	3.22	4.78	4.28	3.79	3.06	3.67	2.90	4.95	3.20	5.10	3.33	4.25	2.96	0.67	1.11	1.19	1.26	4.52	3.91	3.58	2.70	4.78	3.04	4.94	3.67	5.31	3.65	5.27	3.05
KUZ M9884	2.01	2.17	2.30	2.40	3.46	4.03	6.09	4.86	4.23	3.34	5.69	3.54	6.06	4.00	5.67	3.82	4.45	2.49	1.50	1.52	2.04	1.82	5.33	4.29	3.80	2.84	5.34	3.91	5.32	4.06	6.14	3.69	5.81	3.12
KUZ M9976	1.99	2.76	2.51	3.38	3.56	4.18	6.44	5.35	4.88	4.00	5.72	3.67	5.37	3.54	5.96	3.65	5.13	3.13	1.34	1.94	1.74	2.07	5.47	4.42	4.28	3.75	5.92	3.95	6.30	4.08	6.38	3.76	5.97	3.56
KUZ M10370	1.61	1.84	1.91	2.36	2.64	3.35	5.07	4.20	3.97	3.44	4.18	2.96	4.90	2.93	4.92	3.02	4.92	2.94	0.82	1.12	1.38	1.46	4.51	3.60	3.18	2.60	4.69	3.05	5.05	3.19	5.43	3.28	4.90	2.93
TUA 349	2.02	2.11	2.23	2.38	3.01	3.10	5.47	4.82	4.17	3.13	4.88	3.56	5.64	3.94	5.46	3.67	4.78	3.29	1.28	1.61	1.70	1.80	5.06	4.14	3.41	2.84	5.16	3.72	5.33	3.78	5.42	3.70	5.76	3.27
TUA NK502	1.82	2.11	2.18	2.73	3.20	3.70	5.39	4.77	3.73	2.92	4.62	3.02	5.11	3.50	5.32	3.16	4.90	2.95	1.34	1.60	1.73	1.92	4.83	4.25	3.36	2.64	5.09	3.64	5.78	3.63	5.50	3.65	5.87	3.10
TUA P265	1.98	2.33	2.18	2.55	3.11	3.59	5.38	4.14	3.98	3.00	4.70	3.24	5.60	3.13	5.12	3.03	4.31	2.58	1.25	1.51	1.60	1.56	4.73	3.72	3.47	2.94	4.91	3.46	5.32	3.49	5.31	3.41	5.16	3.10
TUA P274	2.06	2.29	2.25	2.48	3.01	3.17	5.91	4.99	4.30	3.41	4.88	3.53	5.13	3.74	5.04	3.60	4.93	3.52	1.01	1.25	1.69	1.69	5.01	4.40	3.46	2.96	5.55	3.77	5.75	4.06	5.33	3.80	4.95	3.65
TUA P275	1.99	2.50	2.04	2.86	3.00	3.29	5.75	4.52	4.28	3.31	4.70	2.92	5.52	3.29	5.40	3.49	4.30	3.08	1.15	1.37	1.65	1.72	4.89	4.17	3.39	2.86	4.93	3.58	5.67	3.63	6.23	3.68	5.21	3.63
TUA P291	1.60	2.02	1.86	2.16	3.24	3.91	5.82	4.76	3.73	3.00	4.85	3.38	5.21	3.36	5.66	3.62	4.78	3.14	1.14	1.41	1.40	1.65	5.16	3.97	3.28	2.84	5.11	3.61	5.35	3.54	5.52	3.65	5.37	3.29
TUA P297	1.96	1.92	2.27	2.59	2.94	3.14	5.00	4.40	3.79	2.85	4.26	2.86	4.26	3.01	4.47	2.94	4.71	2.57	1.14	1.35	1.69	1.44	4.30	3.73	3.26	2.53	4.36	3.28	4.77	3.27	4.62	3.18	4.03	3.13
TUA P300	1.82	1.92	2.05	2.58	3.01	3.21	6.00	4.93	3.98	3.01	4.96	3.48	4.95	3.25	4.69	3.31	4.39	3.04	1.19	1.23	1.84	1.82	5.13	4.54	3.30	2.74	4.77	3.65	5.11	3.47	5.20	3.44	5.08	2.93
TUA RK501	1.74	1.92	1.82	2.19	2.74	3.32	5.30	4.57	4.41	3.40	4.85	3.29	5.68	3.41	5.43	3.46	4.89	2.73	0.99	1.49	1.74	1.76	5.06	3.85	4.06	3.04	5.28	3.90	5.75	3.73	5.99	3.71	5.61	3.11
TUA RK502	1.69	1.81	1.73	1.89	2.26	2.42	4.55	4.03	3.17	2.51	4.09	2.46	4.80	3.14	4.74	3.00	4.04	2.69	1.01	1.28	1.39	1.42	4.27	3.45	2.85	2.33	4.28	3.13	5.09	3.02	4.84	2.95	4.78	2.73
TUA RK505	2.04	2.29	2.16	2.73	3.46	3.82	5.46	4.59	3.58	2.90	4.28	3.04	5.33	3.49	5.02	3.29	4.54	3.01	1.37	1.62	1.79	1.65	5.02	3.87	3.70	2.97	4.93	3.60	5.43	3.63	5.52	3.42	5.52	3.24
TUA RK507	1.27	1.66	1.96	2.01	2.68	3.11	5.12	4.08	3.89	2.89	4.17	2.63	5.25	2.70	5.35	2.92	4.52	2.47	1.37	1.53	1.51	1.83	4.37	3.26	3.56	2.58	4.66	2.81	5.87	2.87	6.10	2.88	5.04	2.83

continued

Table S4. *Continued*

Specimen reference No.	Measurements																																
	LI^1	WI^1	LI^2	WI^2	LI^3	WI^3	LC^1	WC^1	LP^1	WP^1	LP^2	WP^2	LP^3	WP^3	LP^4	WP^4	LM^1	WM^1	LI_2	WI_2	LI_3	WI_3	LC_1	WC_1	LP_1	WP_1	LP_2	WP_2	LP_3	WP_3	LP_4	WP_4	LM_1	WM_1
**Females**																																		
HUM 10	2.00	2.01	2.11	2.47	2.93	3.15	5.24	4.56	4.63	3.64	5.00	3.03	5.78	3.42	4.78	3.37	3.95	2.66	1.02	1.55	2.16	2.18	4.91	4.15	3.26	2.92	4.69	3.58	5.79	3.76	5.88	3.88	5.17	2.90
HUM 44	1.78	2.17	2.04	2.34	2.74	3.62	5.30	4.16	4.18	3.21	5.39	3.21	5.94	3.40	5.38	3.69	5.21	3.45	1.40	1.46	1.75	1.72	4.85	4.01	4.11	3.03	5.15	3.66	6.00	3.79	6.01	3.51	5.82	3.08
HUNHM 17222	1.98	2.20	2.20	2.51	3.50	3.73	5.70	4.80	3.99	3.18	4.84	3.21	4.90	3.38	4.92	3.36	3.66	2.57	1.08	1.39	1.73	1.68	5.70	4.22	3.53	3.01	4.30	3.53	5.16	3.55	4.91	3.71	4.88	3.28
HUNHM 47748	1.75	1.80	2.06	2.22	2.79	2.89	5.34	4.52	3.60	3.03	3.85	2.86	4.57	3.29	4.61	2.88	4.28	2.32	1.05	1.17	1.29	1.33	4.56	4.21	3.32	2.83	4.44	3.13	5.16	3.27	5.05	3.11	5.62	2.77
HUNHM 47749	1.98	2.55	2.31	3.33	3.34	3.86	5.74	4.65	4.05	3.26	4.91	3.34	5.12	3.50	5.23	3.46	4.33	2.78	1.21	1.45	1.81	2.20	5.00	4.10	3.51	2.64	5.05	3.59	5.72	3.87	6.08	3.46	6.32	3.27
KUZ M9312	1.54	1.65	1.62	1.88	2.64	3.28	5.08	3.95	3.28	2.45	3.81	2.58	3.76	2.43	3.75	2.47	3.22	2.13	1.01	1.43	1.42	1.55	4.88	4.08	2.70	2.17	3.87	2.78	4.32	2.84	3.81	2.66	3.36	2.40
KUZ M9314	1.67	2.11	1.96	2.70	3.08	3.74	5.60	4.76	4.07	3.30	4.75	3.40	5.49	3.32	5.39	3.46	4.72	2.68	0.93	1.49	1.52	1.62	5.08	4.06	2.85	2.41	4.76	3.68	5.68	3.69	5.96	3.81	5.35	3.35
KUZ M9325	1.58	2.08	1.78	2.41	2.90	3.33	5.07	4.45	4.13	3.69	4.88	3.50	5.67	3.64	5.78	3.80	4.52	2.61	0.90	1.38	1.48	1.75	4.51	3.92	3.51	3.09	5.10	3.95	6.14	3.93	5.99	3.97	5.41	3.19
KUZ M9334	1.55	2.13	2.13	2.80	3.12	3.55	4.97	4.46	3.83	3.03	4.26	2.76	5.13	2.99	5.23	3.12	4.87	2.58	1.08	1.56	1.64	1.78	4.35	3.73	3.16	2.75	4.90	3.35	5.20	3.45	5.46	3.22	5.50	2.87
KUZ M9338	1.87	2.17	2.40	2.70	3.20	3.68	5.28	4.68	4.14	3.66	5.26	3.84	5.96	3.68	5.44	3.49	4.92	2.86	0.87	1.40	1.99	2.01	5.26	4.41	3.76	3.15	5.68	3.93	5.92	4.02	6.00	3.67	5.83	3.43
KUZ M9341	1.94	2.14	2.17	2.97	3.08	3.47	5.92	4.82	3.49	3.15	4.67	3.04	5.11	3.13	5.58	3.34	4.58	2.93	1.13	1.41	1.70	1.83	5.63	4.29	3.00	2.48	4.59	3.22	5.16	3.37	5.73	3.43	5.58	3.09
KUZ M9342	1.68	2.14	1.93	2.34	2.91	3.47	4.88	3.75	3.46	2.57	4.68	3.23	4.76	3.18	4.39	3.17	3.28	1.88	0.89	1.39	1.57	1.72	5.10	3.60	3.31	2.71	4.46	3.49	5.20	3.52	5.33	3.27	4.16	2.83
KUZ M9411	1.89	2.14	2.25	2.60	2.68	3.54	5.59	4.81	3.98	3.27	3.96	2.81	5.09	3.54	4.76	3.32	4.60	3.00	1.49	1.75	1.82	1.96	5.40	3.98	3.66	2.94	4.77	3.46	5.01	3.50	4.92	3.25	5.15	3.33
KUZ M9427	1.86	2.24	2.03	2.42	2.97	4.04	5.60	4.50	4.83	3.57	5.78	3.11	5.99	3.41	5.84	3.43	4.61	2.67	1.30	1.46	1.89	1.88	5.06	3.88	3.91	3.09	5.48	3.58	5.75	3.34	5.92	3.51	5.52	2.86
KUZ M9453	1.85	2.25	1.95	2.57	2.84	3.32	5.89	4.79	4.00	3.27	4.73	3.01	5.36	3.49	5.65	3.49	5.12	3.06	1.02	1.38	1.95	2.04	5.43	4.18	3.48	2.99	5.12	3.70	5.78	3.84	5.97	3.77	5.23	3.25
KUZ M9469	2.13	2.70	2.49	3.69	3.06	4.11	6.04	4.96	4.36	3.41	4.71	3.19	6.15	3.23	5.74	3.19	4.78	2.60	1.30	1.87	2.01	2.37	5.79	4.18	4.16	3.11	5.40	3.54	5.96	3.68	6.13	3.47	6.25	2.97
KUZ M9473	1.44	1.63	1.69	1.94	2.24	2.98	4.18	3.96	3.38	3.02	4.33	3.16	4.57	3.05	4.56	3.13	3.91	2.24	0.96	1.12	1.16	1.40	3.48	3.08	2.74	2.35	4.51	3.20	4.60	3.12	4.90	3.14	5.21	2.72
KUZ M9482	2.27	2.52	2.80	2.92	3.46	3.51	6.06	5.32	4.53	3.66	4.65	3.57	5.68	3.88	5.60	3.77	5.36	3.66	1.82	2.06	2.35	2.33	5.39	4.63	4.04	3.47	4.87	3.86	5.67	4.04	6.22	3.80	6.06	3.52
KUZ M9483	1.83	2.20	2.21	2.60	2.98	3.28	4.97	4.30	4.37	3.23	4.13	2.77	5.66	3.18	5.66	3.02	4.67	2.61	1.13	1.34	1.82	1.82	4.64	3.68	3.65	2.86	4.77	3.34	6.05	3.35	5.88	3.37	5.57	2.80
KUZ M9500	1.67	1.95	2.03	2.33	2.86	3.00	4.48	3.85	3.83	3.43	3.97	3.21	4.45	3.51	4.92	3.24	3.80	2.20	1.09	1.32	1.38	1.68	4.00	3.34	3.15	2.67	3.99	3.29	4.41	3.40	4.78	3.33	4.75	2.55
KUZ M9501	1.68	1.83	1.88	2.07	2.92	3.23	5.99	5.13	4.36	3.59	5.37	3.70	6.33	3.94	6.40	3.80	5.71	2.97	1.34	1.59	1.35	1.72	4.91	4.27	3.87	3.32	5.66	4.07	6.28	4.09	6.59	3.89	6.28	3.40
KUZ M9550	1.84	1.81	2.01	2.31	2.71	2.92	5.40	4.60	3.76	3.00	4.61	3.08	5.46	3.27	5.20	3.50	5.10	3.16	1.14	1.57	1.49	1.94	4.73	4.14	3.34	2.56	4.69	3.26	5.26	3.49	5.45	3.48	6.20	3.16
KUZ M9556	1.42	2.14	2.00	2.47	3.08	3.24	5.10	4.37	4.14	2.89	4.69	2.85	5.95	3.60	5.80	3.69	4.57	3.10	0.87	1.58	1.36	1.73	4.40	3.78	3.65	2.75	5.02	3.59	6.18	3.81	5.86	3.76	4.87	3.55
KUZ M9569	1.53	2.03	1.73	2.81	2.60	3.14	5.26	4.63	3.49	3.01	4.74	3.47	5.56	3.46	4.72	3.43	4.06	2.57	0.81	1.16	1.45	1.58	5.01	3.66	3.38	2.79	4.93	3.44	5.40	3.58	5.60	3.57	4.81	3.05
KUZ M9571	1.73	1.70	1.87	2.00	2.62	2.63	4.78	3.88	4.29	3.02	4.78	2.69	5.46	3.12	5.03	2.98	4.14	2.53	1.02	1.42	1.40	1.52	4.14	3.38	3.45	2.72	5.05	3.13	6.11	3.15	5.49	3.18	5.62	2.89
KUZ M9574	1.76	2.06	1.94	2.35	2.31	3.62	5.31	4.86	4.09	3.35	4.38	3.38	4.75	3.50	5.29	3.53	4.85	2.79	1.04	1.52	1.48	1.86	5.10	4.12	3.50	2.96	4.14	3.20	5.00	3.49	5.23	3.53	4.12	3.11
KUZ M9607	1.71	1.89	2.15	2.60	3.24	4.11	5.45	4.75	3.93	2.95	4.55	3.27	5.97	3.57	5.73	3.62	5.30	2.48	1.26	1.67	1.59	1.95	5.24	4.06	3.44	2.76	5.20	3.73	6.21	3.97	6.13	3.76	5.78	3.11
KUZ M9617	1.87	2.11	2.08	2.49	2.89	3.77	4.80	4.66	3.38	2.96	3.60	2.79	4.76	3.15	5.01	3.12	4.08	2.77	0.96	1.18	1.47	2.06	4.39	3.84	2.72	2.38	3.92	3.16	4.87	3.44	5.06	3.49	5.60	3.11
KUZ M9621	1.63	1.92	1.86	2.15	2.86	3.47	4.95	4.36	3.51	2.95	3.96	2.69	4.55	3.13	4.37	2.87	3.75	2.61	1.14	1.24	1.18	1.36	4.08	3.54	2.79	2.61	4.07	3.09	4.40	3.14	4.02	3.07	4.35	2.49
KUZ M9646	1.88	2.03	2.28	2.52	2.92	3.08	5.09	4.68	3.93	3.51	4.43	3.28	4.96	3.85	4.57	3.33	4.11	2.94	1.13	1.34	1.66	1.68	4.64	3.97	3.48	3.07	4.52	3.64	4.90	3.79	5.11	3.62	4.73	3.33
KUZ M9653	1.64	1.88	1.69	2.12	2.46	3.34	5.12	4.58	3.73	2.98	5.39	3.13	5.50	3.02	5.11	3.01	4.47	2.65	1.11	1.33	1.56	1.66	4.48	3.81	3.05	2.76	4.48	3.13	5.33	3.11	5.60	3.11	5.17	3.03
KUZ M9654	1.79	2.16	2.10	2.67	2.84	3.81	5.71	4.70	4.28	3.29	4.81	3.28	5.95	3.83	5.53	4.09	4.66	3.09	1.11	1.34	1.74	1.76	5.15	4.28	3.78	3.21	5.52	3.87	6.22	3.96	6.65	4.15	6.24	3.44
KUZ M9660	1.46	1.81	1.71	2.25	2.63	3.11	5.11	4.28	3.62	3.13	3.90	2.50	5.38	2.82	5.48	2.93	4.33	2.53	0.88	1.25	1.25	1.58	4.40	3.56	3.52	2.83	4.72	3.32	5.46	3.35	5.36	3.24	5.11	2.77
KUZ M9663	1.69	1.88	1.86	2.44	3.00	3.30	5.52	4.72	3.94	2.65	4.83	3.24	5.20	3.19	5.02	3.20	4.42	2.80	0.73	1.23	1.42	1.50	4.82	3.48	3.45	2.48	5.10	3.17	5.75	3.50	5.91	3.22	4.29	2.90
KUZ M9667	1.76	1.98	2.05	2.14	2.85	3.17	4.80	4.30	4.33	3.14	4.65	2.75	5.39	2.94	5.00	2.97	4.34	2.64	1.08	1.54	1.67	1.78	4.15	3.74	3.90	2.75	5.05	3.10	5.43	3.22	5.85	3.16	6.05	3.06
KUZ M9668	1.74	2.23	2.10	2.79	3.03	3.65	5.09	4.26	3.82	2.86	4.46	3.11	5.22	3.09	5.25	3.19	4.63	2.49	0.92	1.57	1.37	1.68	4.87	3.76	3.21	2.55	4.98	3.46	5.59	3.50	5.84	3.52	5.27	2.71
KUZ M9670	1.76	2.06	1.82	2.45	2.86	3.88	5.01	4.38	4.05	3.33	4.85	3.02	5.61	3.37	5.41	3.24	4.03	2.85	1.16	1.43	1.54	1.76	4.45	3.87	3.41	2.84	5.17	3.68	5.61	3.81	5.92	3.69	5.22	3.34
KUZ M9674	1.87	2.17	2.45	2.91	3.30	3.86	5.94	4.93	3.90	3.13	5.24	3.33	5.94	3.91	5.88	3.84	5.10	2.96	1.00	1.29	1.64	1.76	5.55	4.24	3.35	2.73	5.23	3.66	5.70	3.85	5.94	4.03	5.86	3.19
KUZ M9677	1.98	2.76	2.24	2.93	2.99	3.66	5.39	4.42	4.02	2.93	4.50	2.83	6.12	3.29	5.86	3.25	5.18	3.18	1.27	1.56	1.57	1.83	4.83	3.73	3.39	2.85	5.21	3.11	6.28	3.12	6.45	3.12	6.12	3.43
KUZ M9678	1.67	1.95	2.05	2.17	2.88	3.63	5.70	4.72	4.57	3.42	5.02	3.15	5.63	3.37	5.71	3.48	5.18	2.86	1.28	1.44	1.29	1.69	5.46	4.27	4.14	3.01	5.30	3.60	5.61	3.71	5.86	3.57	6.49	3.28
KUZ M9679	1.73	1.83	2.03	2.20	3.08	3.92	5.81	5.13	4.35	3.56	4.69	3.32	5.20	3.50	4.94	3.61	4.46	3.05	1.24	1.94	1.64	1.97	5.00	4.43	3.04	2.71	4.72	3.76	5.19	3.91	5.69	3.90	5.63	3.60
KUZ M9684	1.80	2.18	1.98	2.35	2.80	2.97	5.15	4.61	3.65	3.09	3.87	2.66	5.29	3.08	5.17	3.09	5.12	2.95	0.78	1.27	1.46	1.54	4.65	3.99	3.57	2.88	5.00	3.12	5.89	3.11	6.19	3.31	6.10	2.90
KUZ M9687	1.70	1.71	1.71	2.00	2.38	2.51	5.10	4.25	4.38	3.16	4.61	3.02	5.30	3.39	5.12	3.54	4.97	3.20	1.28	1.49	1.95	2.04	4.33	3.77	3.76	2.79	4.65	3.32	5.34	3.33	6.02	3.67	5.58	3.35

continued

Table S4. *Continued*

Specimen reference No.	Measurements																																			
	LI^1	WI^1	LI^2	WI^2	LI^3	WI^3	LC^1	WC^1	LP^1	WP^1	LP^2	WP^2	LP^3	WP^3	LP^4	WP^4	LM^1	WM^1	LI_1	WI_1	LI_2	WI_2	LI_3	WI_3	LC_1	WC_1	LP_1	WP_1	LP_2	WP_2	LP_3	WP_3	LP_4	WP_4	LM_1	WM_1
**Females**																																				
KUZ M9691	1.64	1.80	2.05	2.48	3.09	3.34	5.79	4.69	4.38	3.23	4.55	2.99	5.78	3.39	5.76	3.40	4.83	2.70	1.08	1.47	1.65	1.74	5.19	4.33	3.55	2.79	4.98	3.35	6.21	3.53	5.88	3.35	6.41	3.19		
KUZ M9692	1.49	1.67	1.90	2.19	2.88	2.98	5.23	4.50	3.78	3.52	4.31	3.09	4.95	3.37	4.76	3.66	4.23	2.79	1.10	1.36	1.35	1.95	5.11	4.54	4.11	3.40	4.85	3.41	5.58	3.52	5.15	3.22	4.95	3.06		
KUZ M9708	1.55	1.76	1.63	2.06	2.84	2.95	5.25	4.56	3.54	3.37	4.33	3.35	4.77	3.33	5.13	3.11	4.56	2.86	0.86	1.29	1.17	1.82	4.64	3.78	3.15	2.38	4.82	3.50	5.42	3.66	5.81	3.76	5.64	2.97		
KUZ M9712	1.49	1.86	1.95	2.35	3.05	3.53	5.28	4.70	3.84	3.20	4.66	2.72	5.42	3.17	5.48	3.48	4.64	2.87	0.88	1.48	1.18	1.52	4.65	4.07	3.65	2.84	4.73	3.18	5.45	3.51	6.32	3.32	5.72	2.86		
KUZ M9713	1.14	1.28	1.42	1.79	2.54	2.83	4.67	3.94	3.62	2.81	3.86	2.51	4.03	2.78	4.19	2.44	3.19	2.08	0.81	1.11	1.09	1.21	4.24	3.54	2.60	2.10	4.21	2.92	4.36	3.00	4.22	2.61	3.28	2.17		
KUZ M9714	1.73	1.88	2.00	2.44	3.43	3.55	5.95	4.40	3.82	3.33	4.75	3.03	5.52	3.18	5.44	3.40	5.12	3.25	0.82	1.02	1.45	1.58	5.59	3.74	3.32	2.68	4.65	3.20	5.20	3.48	6.33	3.51	5.98	3.30		
KUZ M9715	1.60	1.85	1.85	2.39	2.80	3.64	5.42	4.43	3.88	3.29	4.22	3.17	4.84	3.24	5.51	3.38	4.53	2.37	0.79	1.20	1.24	1.65	4.69	3.83	3.40	2.76	4.54	3.19	4.83	3.62	5.02	3.07	4.89	2.64		
KUZ M9718	1.76	2.08	2.08	2.58	2.98	3.53	5.12	4.57	3.73	3.17	4.26	3.11	5.03	3.30	5.19	3.30	4.84	3.32	1.18	1.56	1.54	1.74	4.47	3.74	3.43	2.67	4.40	3.25	5.10	3.46	5.23	3.33	4.29	3.17		
KUZ M9755	1.53	2.03	1.61	2.49	3.03	3.44	5.23	4.56	3.58	2.97	5.29	3.33	5.29	3.16	5.50	3.42	5.08	2.97	1.08	1.43	1.60	1.89	5.01	3.91	3.18	2.58	5.14	3.36	5.23	3.53	5.73	3.50	5.54	2.73		
KUZ M9756	1.55	1.95	2.07	2.58	3.02	3.39	5.70	5.03	3.80	3.21	4.68	3.27	5.22	3.10	5.15	3.34	4.30	2.51	1.36	1.57	1.77	2.27	5.06	4.56	3.25	2.92	5.07	3.55	5.49	3.42	5.71	3.37	5.83	2.77		
KUZ M9763	1.72	2.13	1.82	2.48	3.18	3.67	5.37	4.58	4.14	3.37	3.97	2.63	5.37	3.32	5.22	3.16	5.19	3.18	1.06	1.80	1.52	2.13	4.63	3.89	3.65	2.88	5.13	3.42	5.50	3.58	5.88	3.62	5.71	3.31		
KUZ M9766	1.69	1.96	1.89	2.46	2.58	3.08	5.04	4.18	4.00	3.58	4.50	2.87	4.93	2.99	4.58	3.19	4.12	2.66	1.25	1.48	1.63	1.69	4.53	3.62	3.59	2.63	4.90	2.91	5.25	3.16	5.02	3.14	4.58	2.88		
KUZ M9768	1.55	1.78	2.03	2.33	3.03	3.51	5.12	4.39	4.06	3.12	4.55	3.19	5.35	3.32	5.35	3.42	4.77	2.81	1.10	1.37	1.28	1.78	4.54	3.75	3.11	2.63	4.34	3.32	5.15	3.44	5.50	3.55	5.65	3.11		
KUZ M9769	1.78	2.12	1.87	2.39	2.85	3.16	5.08	4.24	4.30	3.04	4.87	3.19	5.79	3.26	5.35	3.67	4.70	3.12	1.22	1.88	1.79	1.92	4.88	3.85	3.72	2.82	5.08	3.61	5.84	3.58	5.45	3.83	5.42	3.44		
KUZ M9772	1.64	2.12	1.72	2.66	2.84	3.67	5.10	3.99	4.24	3.23	5.15	3.06	5.96	3.34	5.78	3.39	4.26	2.29	0.93	1.56	1.30	1.87	4.82	3.65	3.95	2.96	6.11	3.56	6.57	3.52	6.68	3.44	5.53	2.91		
KUZ M9809	1.77	2.20	2.06	2.54	3.05	3.69	5.38	4.72	3.89	3.28	4.56	3.35	4.92	3.07	4.90	3.17	3.98	2.69	1.21	1.64	1.70	1.96	4.92	4.30	3.20	2.99	4.28	3.66	5.16	3.77	5.14	3.72	4.85	3.12		
KUZ M9811	1.53	1.58	2.01	2.21	2.87	3.10	5.42	4.72	4.08	2.90	4.16	2.88	5.12	2.89	5.12	2.93	4.04	2.26	0.74	1.60	1.09	1.66	5.12	4.07	3.42	2.62	4.96	3.21	5.34	3.36	5.52	3.13	5.00	2.66		
KUZ M9826	1.87	1.88	2.01	2.20	3.00	3.10	5.55	4.68	3.72	2.95	4.84	3.06	5.09	3.26	5.04	3.31	3.96	2.52	1.36	1.93	1.67	2.12	5.08	3.84	3.41	2.75	5.01	3.44	5.44	3.30	5.71	3.34	4.19	2.90		
KUZ M9827	1.71	2.01	1.90	2.51	2.61	3.04	4.82	3.80	4.13	3.02	4.60	2.97	5.03	3.00	4.98	2.97	3.93	2.39	1.06	1.28	1.44	1.77	4.11	3.26	3.48	2.79	4.68	2.85	5.49	3.05	5.52	3.25	5.02	2.97		
KUZ M9828	1.71	2.22	1.96	2.38	2.62	3.46	5.40	4.40	3.79	2.91	3.85	2.82	5.08	3.25	4.72	3.16	4.08	2.78	0.99	1.40	1.67	1.87	4.85	3.70	3.18	2.48	4.53	3.30	5.32	3.09	4.88	3.03	4.04	2.67		
KUZ M9829	1.71	1.78	2.01	2.38	3.17	3.59	5.68	4.76	3.28	2.88	4.50	2.99	4.95	3.28	5.42	3.30	4.49	2.63	1.00	1.56	1.48	1.85	4.72	3.72	3.01	2.40	4.69	3.10	5.60	3.34	5.03	2.95	4.44	2.94		
KUZ M9831	1.90	2.27	2.05	2.70	3.00	3.96	5.51	4.64	4.08	3.24	4.98	3.14	5.82	3.08	5.25	3.10	5.14	2.94	1.10	1.59	1.65	1.90	4.98	4.09	3.70	3.09	4.76	3.12	5.20	3.38	5.40	3.20	5.69	2.80		
KUZ M9832	1.82	1.90	2.28	2.43	2.57	2.97	5.15	4.10	4.44	3.25	4.68	3.07	5.48	3.26	5.04	3.16	4.24	2.74	1.26	1.51	1.79	1.77	4.21	3.27	3.89	2.93	4.45	3.39	5.12	3.56	5.58	3.42	4.36	3.12		
KUZ M9835	1.62	2.12	1.86	2.46	2.78	3.66	6.39	4.71	3.56	2.79	4.48	2.74	5.57	2.95	4.84	3.09	4.64	2.72	0.87	1.30	1.18	1.80	5.42	4.09	3.26	2.48	4.95	3.36	5.77	3.73	5.92	3.88	5.97	3.51		
KUZ M9836	1.91	2.63	2.25	2.86	3.39	4.09	5.52	4.83	5.11	3.70	5.10	3.48	6.26	3.92	5.88	3.72	5.24	2.86	1.05	1.44	1.82	1.96	5.15	4.15	4.25	3.35	5.57	3.94	5.91	4.16	6.56	4.06	6.26	3.33		
KUZ M9857	1.68	1.90	1.90	2.33	2.77	3.34	5.00	4.12	3.76	3.01	4.16	2.84	5.06	3.09	5.28	3.35	4.68	2.59	0.98	1.70	1.61	1.78	4.96	3.87	3.23	2.44	4.94	3.06	5.34	3.41	5.80	3.44	5.05	2.93		
KUZ M9859	1.75	2.04	1.89	2.43	2.68	2.87	4.79	4.23	3.50	3.31	3.33	2.79	5.15	3.29	5.16	3.46	4.59	2.81	1.04	1.65	1.75	1.76	4.68	3.74	3.58	2.78	4.65	3.59	5.48	3.43	5.38	3.48	5.37	3.22		
KUZ M9863	1.58	1.80	2.01	2.25	2.55	3.07	5.28	4.51	3.39	2.99	4.24	3.13	4.80	3.42	4.70	3.10	4.37	2.60	0.79	1.19	1.28	1.90	5.00	4.05	3.06	2.59	3.82	3.29	4.72	3.29	4.90	3.16	4.79	2.64		
KUZ M9870	1.69	2.01	1.76	2.42	2.65	3.13	5.02	4.49	3.76	2.82	4.19	2.84	5.50	3.25	5.23	3.30	4.51	2.59	0.74	1.23	1.34	1.40	4.51	3.73	3.25	2.60	3.98	3.13	5.20	3.57	5.30	3.63	6.08	2.86		
KUZ M9871	1.72	2.26	2.24	3.36	2.70	3.98	5.83	4.97	4.09	3.58	4.75	3.51	5.54	3.62	5.24	3.49	4.66	2.85	1.29	1.56	1.73	1.78	5.01	4.20	3.55	3.07	4.65	3.98	5.34	3.90	5.81	3.76	5.57	3.25		
KUZ M9872	1.43	1.75	1.62	2.46	2.39	3.14	4.87	4.06	3.33	2.80	3.97	3.20	4.46	3.25	3.96	2.97	4.06	2.27	0.87	1.09	1.14	1.23	4.11	3.12	3.47	2.55	4.00	3.17	4.36	3.33	4.37	3.21	4.69	2.85		
KUZ M9877	2.10	2.29	2.25	2.96	3.28	3.33	5.21	4.45	4.13	3.30	4.67	3.06	4.64	3.56	5.52	3.26	3.90	2.53	1.09	1.34	1.75	1.50	4.76	3.99	3.41	2.81	4.95	3.51	5.63	3.68	5.94	3.70	5.29	3.12		
KUZ M9878	1.36	1.71	1.79	2.18	2.76	3.11	5.26	4.32	3.93	3.16	4.34	2.86	4.76	3.10	4.36	2.98	3.93	2.33	0.91	1.56	1.51	1.86	4.50	3.62	3.42	2.62	4.70	3.08	5.10	3.22	5.18	3.34	4.86	2.87		
KUZ M9879	1.12	1.29	1.42	1.67	2.69	2.92	4.42	3.69	3.50	2.78	3.94	2.61	4.00	2.54	3.98	2.50	3.31	2.13	0.81	0.97	1.06	1.79	4.08	3.25	3.12	2.25	3.84	2.61	4.34	2.70	4.54	2.71	4.46	2.59		
TUA 348b	1.56	1.72	1.66	2.20	2.71	3.01	5.46	4.00	3.92	2.69	4.44	2.80	5.20	3.29	5.08	3.47	4.00	2.72	1.21	1.34	1.51	1.70	4.76	3.58	3.24	2.49	4.74	3.26	5.45	3.38	5.26	3.33	4.78	2.96		
TUA P269	1.58	1.79	1.91	2.21	2.97	3.25	5.59	4.55	4.34	3.38	5.04	3.33	5.89	3.36	5.70	3.43	5.33	2.83	1.33	1.63	1.79	1.96	5.01	3.96	3.62	2.97	5.55	3.62	5.83	3.56	5.94	3.64	6.42	3.39		
TUA P279	1.84	1.89	2.24	2.19	3.02	3.16	4.86	4.56	4.11	3.29	4.71	2.63	5.32	2.96	5.55	3.09	4.84	2.63	1.00	1.30	1.49	1.54	4.82	3.85	4.01	2.95	5.02	3.06	6.08	3.29	5.76	3.07	5.88	2.89		
TUA P294	1.71	1.98	1.97	2.33	2.56	2.92	4.66	4.17	2.77	2.49	4.63	3.06	5.82	3.45	5.84	3.63	4.51	2.73	1.25	1.43	1.82	1.83	4.36	3.81	1.66	1.48	5.03	3.31	5.76	3.72	5.72	3.63	5.29	3.35		
TUA P295	1.67	1.88	1.95	2.92	2.64	3.07	5.14	4.56	3.16	2.57	4.64	3.38	4.68	3.45	4.65	3.24	4.29	2.69	1.17	1.35	1.69	1.68	4.49	3.88	2.97	2.54	5.30	3.54	5.41	3.48	5.18	3.47	5.12	3.09		
TUA P301	1.64	1.91	1.87	2.30	3.16	3.31	5.39	4.33	4.04	2.91	4.70	3.04	5.77	3.44	5.98	3.12	4.76	2.64	0.68	0.89	1.12	1.36	4.83	3.90	3.55	2.84	4.35	3.39	5.69	3.63	6.18	3.63	5.71	3.31		
TUA RK509	1.73	1.71	2.06	2.25	3.06	3.39	5.42	4.79	4.70	4.08	4.94	3.48	5.61	3.70	5.35	3.60	5.24	3.30	1.23	1.50	1.81	1.67	5.20	4.22	3.36	2.84	4.75	3.66	5.36	3.65	5.64	3.74	5.77	3.53		
TUA RK511	1.71	1.98	2.06	2.39	3.27	3.50	5.11	4.38	4.73	3.34	5.09	3.26	6.14	3.48	5.75	3.76	5.56	3.52	1.26	1.62	1.57	1.76	4.97	3.87	3.92	3.17	5.21	3.96	6.08	4.29	6.28	4.29	6.01	3.68		
TUA RK512	1.79	2.20	2.01	2.30	2.95	3.26	4.73	4.22	3.96	2.95	4.82	2.66	5.34	3.01	4.90	2.83	4.41	2.82	1.30	1.67	1.88	1.80	4.29	3.62	2.97	2.65	4.24	3.14	5.02	3.24	4.58	2.97	5.94	2.70		

continued

Table S4. *Continued*

Specimen reference No.	Measurements																																	
	LI^1	WI^1	LI^2	WI^2	LI^3	WI^3	LC^1	WC^1	LP^1	WP^1	LP^2	WP^2	LP^3	WP^3	LP^4	WP^4	LM^1	WM^1	LI_2	WI_2	LI_3	WI_3	LC_1	WC_1	LP_1	WP_1	LP_2	WP_2	LP_3	WP_3	LP_4	WP_4	LM_1	WM_1
**Undetermined gender**																																		
HUM 4	1.74	2.22	2.31	2.57	3.20	3.33	5.32	4.32	3.73	3.02	4.40	2.92	4.81	3.33	4.81	3.44	4.26	2.89	1.39	1.43	1.60	1.61	4.63	3.65	2.90	2.54	4.56	3.49	5.19	3.66	5.51	3.53	5.72	2.94
HUM 30	1.54	1.86	1.79	2.23	2.63	3.12	4.47	3.88	3.18	2.57	3.51	2.35	4.67	2.92	4.76	2.76	3.92	2.45	1.24	1.15	1.74	1.50	4.26	3.38	2.66	2.30	4.20	3.03	4.68	3.01	4.75	2.75	4.46	2.62
HUM 43	1.64	1.81	2.04	1.97	2.77	2.92	5.11	4.80	3.95	3.55	4.25	3.15	5.36	3.43	5.16	3.33	5.40	2.98	1.09	1.29	1.32	1.60	4.84	4.18	3.51	3.03	5.00	3.44	5.64	3.64	5.41	3.44	5.71	3.25
HUNHM 17236	1.77	1.95	1.99	2.24	3.01	3.33	5.39	4.69	4.51	3.22	4.52	3.23	5.27	3.58	4.89	3.39	4.02	2.80	1.32	1.44	1.80	1.96	5.28	4.05	3.63	2.98	4.86	3.56	5.70	3.65	5.53	3.51	5.06	2.83
HUNHM 17239	1.83	2.34	2.36	3.07	3.77	4.03	6.19	5.38	4.01	2.98	5.16	3.52	5.85	3.34	5.68	3.59	5.15	2.77	1.38	1.69	2.04	1.77	5.36	4.41	3.56	2.71	5.63	4.04	6.23	3.59	5.91	3.54	5.55	3.03
KUZ M9352	1.76	1.97	1.99	2.52	2.91	3.73	5.52	4.72	3.80	2.99	4.68	3.39	5.71	3.39	5.26	3.30	4.13	2.36	1.09	1.23	1.92	1.95	5.29	4.11	3.70	2.90	5.21	3.51	5.54	3.44	6.06	3.31	5.82	3.00
KUZ M9353	1.58	1.92	2.02	2.54	2.97	3.70	5.43	4.70	3.81	3.18	4.97	3.38	5.74	3.58	5.98	3.50	4.89	2.81	1.13	1.31	1.45	1.40	4.80	3.90	3.98	3.00	4.86	3.72	5.51	3.81	5.79	3.85	5.96	3.48
KUZ M9358	1.93	1.90	2.10	2.05	3.10	3.02	4.79	3.88	4.25	3.26	5.08	2.99	5.25	2.90	5.53	3.27	4.67	2.57	0.97	1.50	1.74	1.83	4.56	3.50	3.96	3.10	4.91	3.11	5.08	3.22	5.23	3.07	3.91	3.01
KUZ M9359	1.61	1.90	1.95	2.24	3.06	3.28	5.28	4.48	4.20	3.13	5.68	3.05	5.51	3.01	5.11	3.16	4.64	2.76	1.15	1.45	1.62	1.63	4.82	3.83	3.43	2.91	4.87	3.47	5.64	3.37	5.72	3.64	5.16	3.16
KUZ M9360	2.19	2.47	2.54	3.08	3.50	4.11	5.48	5.01	4.97	3.96	5.83	3.95	5.46	3.83	5.07	3.52	4.81	2.89	1.56	1.62	2.14	2.21	5.20	4.49	4.91	3.59	5.85	4.02	5.98	4.15	6.08	3.85	5.94	3.41
KUZ M9361	1.66	1.93	2.05	2.36	2.96	4.11	5.62	4.96	3.56	2.96	3.91	3.01	4.79	3.45	4.79	3.41	4.53	2.99	0.89	1.20	1.49	1.74	4.97	4.32	3.10	2.71	4.72	3.52	5.21	3.68	5.17	3.65	4.94	3.06
KUZ M9380	1.78	2.15	2.22	2.76	2.95	3.62	5.43	4.45	3.84	3.00	4.64	2.59	5.41	3.12	5.57	3.21	4.80	2.68	1.11	1.24	1.83	1.62	5.19	3.88	3.33	2.62	4.98	3.25	5.54	3.35	5.58	3.04	5.82	2.59
KUZ M9389	1.56	1.78	1.74	2.18	2.55	2.89	4.47	3.87	3.87	3.15	4.29	3.08	5.17	3.52	4.87	3.35	4.07	2.41	1.54	1.72	1.59	1.97	4.01	3.52	3.21	2.77	4.75	3.54	5.18	3.60	5.16	3.47	5.02	2.84
KUZ M9391	1.76	2.29	2.30	2.55	3.25	3.43	5.11	4.71	3.61	3.04	3.68	3.06	5.06	3.22	4.96	3.45	3.67	2.70	1.52	1.84	1.86	1.93	4.70	4.13	3.20	2.56	4.55	3.69	5.36	3.82	5.53	3.61	5.08	3.38
KUZ M9392	2.05	2.24	2.09	2.27	3.11	3.30	5.66	5.20	4.54	3.61	5.51	3.39	6.04	3.98	5.68	3.61	5.34	3.35	1.42	1.31	1.86	1.82	5.04	4.50	3.97	3.02	5.28	3.95	6.14	4.16	6.22	3.85	6.26	3.33
KUZ M9393	1.77	2.10	1.84	2.83	3.22	3.51	5.58	4.83	4.48	3.54	5.57	3.87	6.52	4.12	6.21	3.90	5.85	3.32	1.07	1.53	1.70	1.84	5.01	4.27	4.15	3.52	5.70	4.06	6.42	4.16	6.66	3.94	6.61	3.31
KUZ M9439	1.55	1.95	2.00	2.34	3.04	3.50	4.40	4.09	3.64	2.56	4.27	2.65	5.26	3.04	5.08	2.99	4.46	2.55	1.12	1.27	1.51	1.50	4.08	3.52	3.09	2.39	4.48	3.13	5.29	3.21	5.26	2.95	4.94	2.74
KUZ M9452	1.69	1.83	1.92	2.29	2.70	3.31	4.63	4.21	4.01	3.16	4.60	3.02	5.19	3.53	4.90	3.29	4.66	2.58	0.71	1.27	1.41	1.39	4.28	3.80	3.12	2.81	4.73	3.67	5.55	3.65	5.62	3.43	5.08	3.16
KUZ M9681	1.46	1.75	1.65	1.93	2.31	3.28	4.60	3.91	3.20	2.71	4.08	2.69	4.66	2.90	4.33	2.81	3.49	2.07	0.68	1.23	1.12	1.87	4.04	3.12	2.69	2.35	4.46	2.90	5.53	3.41	5.05	3.27	5.65	2.79
KUZ M10330	2.05	2.53	2.12	2.72	3.07	3.59	6.40	5.33	4.65	4.09	5.22	4.09	5.78	4.14	5.47	4.18	5.59	3.75	1.40	1.88	2.13	2.35	5.88	4.75	4.34	3.71	5.71	4.18	6.64	4.59	6.73	4.73	7.14	4.53
KUZ M10344	1.96	2.24	2.36	2.55	3.30	3.31	5.79	5.05	4.44	3.33	5.49	3.53	5.64	3.61	5.36	3.64	4.90	2.66	0.99	1.62	1.81	1.70	5.20	4.22	3.98	3.03	5.58	3.54	5.92	3.58	6.18	3.54	5.55	3.24
KUZ M10346	1.79	2.12	1.92	2.64	3.13	3.58	5.25	5.05	4.02	3.09	4.58	3.17	4.72	3.37	5.42	3.44	4.40	2.62	1.13	1.52	1.82	1.74	5.11	4.59	3.22	2.86	5.21	3.51	5.50	3.57	5.45	3.13	5.83	2.81
KUZ M10347	2.25	2.46	2.31	2.64	3.45	4.11	6.23	5.50	5.23	3.89	6.23	3.95	6.72	3.96	5.88	3.99	6.59	4.34	1.50	1.77	1.83	1.84	5.80	4.51	6.43	4.56	6.93	4.55	6.92	4.58	6.89	4.27	6.65	4.32
KUZ M10348	1.75	2.14	2.38	2.76	2.63	2.87	4.92	4.12	3.61	3.06	3.59	2.89	4.93	3.21	4.56	3.29	4.35	2.52	1.11	1.57	1.50	1.87	4.59	3.58	3.52	2.97	5.08	3.32	5.31	3.26	5.18	3.42	5.61	3.29
KUZ M10350	1.74	1.72	2.06	2.24	2.65	3.41	4.74	4.09	3.68	2.77	4.33	2.60	4.96	3.18	4.82	2.96	4.24	2.68	0.89	1.30	1.48	1.74	4.58	3.56	2.76	2.45	4.47	2.97	5.23	2.95	5.43	2.99	4.68	2.62
KUZ M10351	1.40	1.71	1.74	2.01	2.95	3.13	5.59	4.26	4.54	3.22	5.09	3.26	5.91	3.37	4.99	3.24	5.09	2.72	0.87	1.42	1.20	1.64	4.60	3.47	3.56	2.67	4.82	3.30	5.37	3.54	4.95	3.23	5.54	2.81
KUZ M10352	1.65	1.81	1.96	2.17	2.66	3.03	4.68	4.17	3.97	2.86	4.32	2.85	5.29	3.08	4.88	3.04	3.63	2.26	1.29	1.60	1.86	1.88	3.95	3.66	3.74	2.69	4.88	3.22	5.53	3.20	5.44	3.09	4.61	2.79
KUZ M10354	1.83	2.11	1.97	2.22	2.50	2.81	4.66	4.38	3.52	3.05	4.49	2.99	5.01	2.99	5.10	3.11	4.05	2.52	0.86	1.32	1.34	1.52	4.23	3.72	3.54	2.80	4.48	3.23	5.01	3.31	5.13	3.12	4.74	2.70
KUZ M10355	1.69	2.24	1.73	2.56	2.85	3.94	5.33	4.49	3.92	3.08	5.07	2.97	5.97	3.04	5.80	3.02	5.39	2.75	1.15	1.44	1.54	1.75	4.86	3.71	2.96	2.82	4.98	3.19	5.93	3.17	5.91	2.95	5.97	2.91
KUZ M10356	1.84	2.02	2.40	2.58	3.15	3.64	6.30	5.41	4.27	3.13	4.77	3.17	5.41	3.45	5.54	3.29	4.17	2.56	1.23	1.51	1.83	1.97	5.84	4.77	3.76	2.95	5.14	3.47	6.06	3.71	6.04	3.56	5.68	3.33
KUZ M10357	1.95	2.24	1.97	2.47	2.87	3.83	5.97	5.00	4.43	3.42	5.28	3.01	5.65	3.16	5.94	3.35	5.68	2.74	0.97	1.51	1.68	1.70	5.30	4.30	3.79	2.98	5.26	3.44	5.73	3.49	6.20	3.55	5.97	3.11
KUZ M10358	1.69	1.79	1.86	2.24	2.76	3.09	5.42	4.43	3.71	2.65	4.55	2.98	5.50	3.34	4.76	2.97	4.17	2.32	0.79	1.07	1.38	1.37	4.93	3.74	3.42	2.71	4.54	3.18	5.48	3.13	5.80	3.23	5.15	2.69
KUZ M10359	1.61	2.18	2.01	3.10	2.59	3.38	5.26	5.03	3.72	3.33	3.97	2.76	4.99	3.33	5.11	3.33	4.16	2.60	1.06	1.66	1.59	1.90	5.18	4.32	3.66	3.08	4.81	3.37	5.49	3.37	5.18	3.33	4.77	2.76
KUZ M10360	1.67	1.99	2.10	2.68	2.80	3.80	4.78	4.26	3.44	3.10	4.45	3.19	5.14	3.12	5.28	3.12	3.21	1.80	0.94	1.49	1.37	1.68	4.39	3.70	3.09	2.66	4.91	3.18	5.48	3.29	5.42	2.70	4.83	2.67
KUZ M10368	1.44	1.99	1.89	2.49	2.95	3.15	5.30	4.29	3.86	2.88	4.05	2.64	4.97	3.16	5.40	3.39	5.38	2.89	1.14	1.36	1.34	1.45	5.03	3.78	3.41	2.70	4.45	3.12	5.83	3.36	6.12	3.41	6.23	3.02
NSMT KK85	1.70	2.00	2.03	2.45	3.21	3.56	5.24	4.57	3.88	3.41	5.33	3.70	5.87	3.93	6.09	4.25	6.01	3.65	1.19	1.40	1.73	1.92	5.24	4.35	3.67	3.16	4.99	3.93	5.99	4.07	6.26	4.06	6.51	3.59
NSMT M19749	1.68	2.16	2.05	2.51	2.99	3.73	6.13	5.12	3.99	3.11	4.20	3.00	5.17	3.58	5.09	3.85	4.61	2.77	1.32	1.59	1.71	2.02	5.60	4.35	3.48	2.82	4.70	3.46	5.27	3.90	5.61	3.83	5.47	3.62
NSMT M29081	1.79	2.18	2.19	2.58	2.72	3.11	5.22	4.82	3.32	3.15	4.16	3.21	4.62	3.08	4.65	3.07	3.86	2.43	1.12	1.64	1.49	1.75	4.65	4.15	2.90	2.62	4.37	3.54	4.95	3.52	4.95	3.20	4.56	2.62
NSMT M29082	1.85	2.46	2.16	2.48	2.93	3.33	5.09	4.95	3.30	2.99	5.10	3.58	5.21	3.54	4.97	3.56	5.10	3.50	1.06	1.50	1.56	1.89	4.80	4.07	3.95	3.52	4.65	3.28	4.84	3.56	4.71	3.22	4.13	2.74

Table S5. Descriptive statistics for measurements in *Eumetopias jubatus*. Males and females are combined (61 specimens in total). The observed range, arithmetic mean and standard deviation are in millimeters; the coefficient of variation is in per cent. Measurement abbreviations: L, mesiodistal length of the tooth crown; W, vestibulolingual width of the tooth crown; I, incisor; C, canine; P, premolar; M, molar; superscript and subscript numbers indicate positions of upper and lower teeth, respectively. Note that tooth size is sexually dimorphic in this species, and therefore values of the coefficient of variation (which are here for males and females jointly) are in most cases higher (sometimes considerably) than those for males and females separately (compare with table 7 in Wolsan *et al.* 2015, *PLoS One* 10, e0137100).

Measurement	Observed range	Arithmetic mean	Standard deviation	Coefficient of variation
**Lengths of upper tooth crowns**				
LI^1	3.05–4.19	3.56	0.29	8.19
LI^2	3.59–5.50	4.43	0.44	9.92
LI^3	7.35–13.14	10.07	1.80	17.84
LC^1	10.94–27.26	17.62	4.54	25.78
LP^1	8.47–12.66	10.37	0.89	8.61
LP^2	10.66–15.16	12.45	1.08	8.71
LP^3	10.20–14.49	12.15	1.02	8.40
LP^4	10.15–14.20	11.97	0.92	7.71
LM^1	7.30–13.55	10.74	0.97	9.03
**Widths of upper tooth crowns**				
WI^1	4.54–6.77	5.55	0.61	11.06
WI^2	5.55–8.54	6.76	0.86	12.68
WI^3	9.52–16.26	12.44	2.04	16.41
WC^1	10.09–22.89	14.97	4.09	27.32
WP^1	6.58–9.68	8.18	0.71	8.72
WP^2	8.17–11.10	9.47	0.81	8.54
WP^3	7.74–11.49	9.54	0.82	8.59
WP^4	6.64–10.48	8.27	0.73	8.80
WM^1	5.32–7.67	6.45	0.53	8.26
**Lengths of lower tooth crowns**				
LI_2	3.99–6.04	4.90	0.42	8.62
LI_3	7.17–10.76	8.58	0.73	8.47
LC_1	11.71–28.98	18.40	5.56	30.22
LP_1	6.03–11.57	9.53	0.90	9.49
LP_2	10.09–13.03	11.58	0.83	7.13
LP_3	11.14–15.53	13.27	1.09	8.21
LP_4	9.14–14.60	12.20	1.10	9.01
LM_1	7.81–11.55	9.98	0.86	8.61
**Widths of lower tooth crowns**				
WI_2	4.20–6.81	5.46	0.61	11.12
WI_3	4.74–8.76	6.05	0.78	12.95
WC_1	8.18–18.93	12.53	3.78	30.17
WP_1	5.76–8.70	7.17	0.69	9.69
WP_2	6.45–9.30	7.76	0.72	9.34
WP_3	7.46–10.60	8.74	0.86	9.80
WP_4	6.25–10.10	8.17	0.83	10.21
WM_1	5.40–7.95	6.44	0.58	9.02

Table S6. Descriptive statistics for measurements in *Callorhinus ursinus*. Males and females are combined (102 specimens in total). The observed range, arithmetic mean and standard deviation are in millimeters; the coefficient of variation is in per cent. Measurement abbreviations: L, mesiodistal length of the tooth crown; W, vestibulolingual width of the tooth crown; I, incisor; C, canine; P, premolar; M, molar; superscript and subscript numbers indicate positions of upper and lower teeth, respectively. Note that tooth size is sexually dimorphic in this species, and therefore values of the coefficient of variation (which are here for males and females jointly) are in most cases higher (sometimes considerably) than those for males and females separately (compare with table 6 in Wolsan *et al.* 2015, *PLoS One* 10, e0137100).

Measurement	Observed range	Arithmetic mean	Standard deviation	Coefficient of variation
**Lengths of upper tooth crowns**				
LI^1	1.88–2.71	2.31	0.17	7.29
LI^2	2.18–3.06	2.60	0.18	6.85
LI^3	2.86–5.62	4.04	0.63	15.62
LC^1	6.87–15.51	9.62	2.24	23.27
LP^1	4.86–7.11	5.81	0.44	7.58
LP^2	5.02–6.83	5.87	0.44	7.57
LP^3	5.06–7.07	5.89	0.46	7.82
LP^4	5.08–7.14	6.07	0.48	7.91
LM^1	5.36–7.56	6.46	0.47	7.32
LM^2	2.73–6.45	4.97	0.78	15.70
**Widths of upper tooth crowns**				
WI^1	3.25–5.19	4.03	0.42	10.44
WI^2	3.50–5.48	4.35	0.41	9.52
WI^3	4.28–7.95	5.85	0.91	15.59
WC^1	4.69–11.38	7.24	1.78	24.60
WP^1	3.71–5.45	4.51	0.39	8.56
WP^2	3.68–5.43	4.39	0.38	8.71
WP^3	3.64–5.41	4.39	0.35	7.88
WP^4	3.68–5.09	4.35	0.33	7.51
WM^1	2.82–4.18	3.46	0.28	8.01
WM^2	2.28–3.93	3.10	0.34	10.81
**Lengths of lower tooth crowns**				
LI_2	2.41–4.20	3.06	0.26	8.32
LI_3	4.35–6.93	5.27	0.57	10.72
LC_1	6.08–15.54	9.17	2.72	29.65
LP_1	4.49–6.35	5.26	0.36	6.88
LP_2	5.00–6.86	5.81	0.43	7.32
LP_3	5.39–7.46	6.33	0.49	7.79
LP_4	5.32–7.62	6.44	0.49	7.62
LM_1	5.89–8.33	6.90	0.49	7.10
**Widths of lower tooth crowns**				
WI_2	2.23–3.84	2.92	0.33	11.24
WI_3	2.25–4.53	3.06	0.45	14.76
WC_1	4.04–8.99	5.90	1.43	24.30
WP_1	2.90–4.43	3.76	0.30	8.05
WP_2	3.08–4.99	4.01	0.38	9.37
WP_3	3.39–5.30	4.26	0.38	8.99
WP_4	3.39–5.43	4.39	0.38	8.58
WM_1	3.32–4.81	3.94	0.31	7.75

Table S7. Descriptive statistics for measurements in *Phoca largha*. Males and females are combined (192 specimens in total). The observed range, arithmetic mean and standard deviation are in millimeters; the coefficient of variation is in per cent. Measurement abbreviations: L, mesiodistal length of the tooth crown; W, vestibulolingual width of the tooth crown; I, incisor; C, canine; P, premolar; M, molar; superscript and subscript numbers indicate positions of upper and lower teeth, respectively. Note that tooth size is sexually dimorphic in this species, and therefore values of the coefficient of variation (which are here for males and females jointly) are in most cases higher than those for males and females separately (compare with table 9 in Wolsan *et al.* 2015, *PLoS One* 10, e0137100).

Measurement	Observed range	Arithmetic mean	Standard deviation	Coefficient of variation
**Lengths of upper tooth crowns**				
LI^1	1.61–2.43	2.01	0.14	6.93
LI^2	2.03–2.88	2.47	0.18	7.37
LI^3	2.77–4.74	3.73	0.29	7.73
LC^1	6.38–10.66	7.90	0.69	8.76
LP^1	4.64–7.54	5.71	0.48	8.35
LP^2	6.56–10.29	7.84	0.62	7.94
LP^3	7.12–10.30	8.39	0.59	7.01
LP^4	6.32–9.59	8.03	0.56	7.01
LM^1	6.07–9.34	7.73	0.60	7.76
**Widths of upper tooth crowns**				
WI^1	2.25–3.77	2.94	0.29	10.02
WI^2	2.55–4.59	3.46	0.36	10.51
WI^3	3.58–5.96	4.92	0.42	8.55
WC^1	5.07–8.10	6.61	0.53	7.96
WP^1	3.02–4.77	3.86	0.30	7.84
WP^2	3.50–5.41	4.22	0.33	7.89
WP^3	3.56–5.28	4.44	0.33	7.49
WP^4	3.72–5.53	4.57	0.32	7.05
WM^1	3.46–5.22	4.36	0.35	7.99
**Lengths of lower tooth crowns**				
LI_2	1.55–2.31	1.89	0.15	7.91
LI_3	1.85–3.10	2.52	0.20	7.87
LC_1	5.72–9.19	7.20	0.58	7.99
LP_1	4.25–6.62	5.32	0.40	7.55
LP_2	6.64–9.76	8.00	0.57	7.09
LP_3	7.40–10.42	8.81	0.62	6.98
LP_4	7.16–10.02	8.39	0.57	6.78
LM_1	7.54–10.96	9.11	0.64	7.06
**Widths of lower tooth crowns**				
WI_2	1.58–2.68	2.09	0.18	8.52
WI_3	1.99–3.09	2.50	0.20	7.87
WC_1	4.98–7.60	5.93	0.48	8.10
WP_1	2.84–4.67	3.83	0.31	8.01
WP_2	3.80–5.55	4.62	0.33	7.25
WP_3	3.79–5.61	4.71	0.34	7.32
WP_4	3.69–5.46	4.51	0.32	7.08
WM_1	3.60–5.29	4.51	0.35	7.74

Table S8. Descriptive statistics for measurements in *Histriophoca fasciata*. Males and females are combined (187 specimens in total). The observed range, arithmetic mean and standard deviation are in millimeters; the coefficient of variation is in per cent. Measurement abbreviations: L, mesiodistal length of the tooth crown; W, vestibulolingual width of the tooth crown; I, incisor; C, canine; P, premolar; M, molar; superscript and subscript numbers indicate positions of upper and lower teeth, respectively. For comparable data from males and females separately, see table 8 in Wolsan *et al.* 2015, *PLoS One* 10, e0137100.

Measurement	Observed range	Arithmetic mean	Standard deviation	Coefficient of variation
**Lengths of upper tooth crowns**				
LI^1	1.12–2.28	1.75	0.20	11.35
LI^2	1.42–2.80	2.02	0.23	11.41
LI^3	2.23–3.82	2.93	0.30	10.13
LC^1	4.18–6.62	5.37	0.48	8.96
LP^1	2.77–5.34	4.00	0.44	10.88
LP^2	3.33–6.23	4.65	0.53	11.51
LP^3	3.76–6.83	5.32	0.51	9.56
LP^4	3.75–6.94	5.22	0.49	9.46
LM^1	3.19–6.59	4.60	0.57	12.31
**Widths of upper tooth crowns**				
WI^1	1.28–2.88	2.05	0.26	12.90
WI^2	1.67–3.69	2.47	0.33	13.41
WI^3	2.42–4.21	3.42	0.37	10.92
WC^1	3.69–5.62	4.56	0.39	8.52
WP^1	2.45–4.21	3.19	0.34	10.73
WP^2	2.35–4.47	3.13	0.35	11.27
WP^3	2.43–4.41	3.34	0.32	9.61
WP^4	2.44–4.28	3.33	0.32	9.70
WM^1	1.80–4.34	2.78	0.36	13.03
**Lengths of lower tooth crowns**				
LI_2	0.62–1.82	1.10	0.20	18.33
LI_3	1.05–2.35	1.60	0.26	16.32
LC_1	3.48–6.13	4.88	0.47	9.58
LP_1	1.66–6.43	3.50	0.47	13.32
LP_2	3.82–6.93	4.90	0.49	10.09
LP_3	4.32–7.76	5.54	0.53	9.50
LP_4	3.81–7.28	5.62	0.57	10.16
LM_1	3.28–7.64	5.39	0.67	12.49
**Widths of lower tooth crowns**				
WI_2	0.89–2.06	1.44	0.21	14.36
WI_3	1.21–2.94	1.76	0.24	13.69
WC_1	3.08–5.13	3.95	0.36	9.12
WP_1	1.48–4.56	2.83	0.34	12.16
WP_2	2.61–4.55	3.44	0.33	9.55
WP_3	2.70–4.59	3.56	0.34	9.55
WP_4	2.61–4.73	3.48	0.35	10.08
WM_1	2.17–4.53	3.09	0.34	11.01
